# Discordant detection of avian influenza virus subtypes in time and space between poultry and wild birds; Towards improvement of surveillance programs

**DOI:** 10.1371/journal.pone.0173470

**Published:** 2017-03-09

**Authors:** Josanne H. Verhagen, Pascal Lexmond, Oanh Vuong, Martin Schutten, Judith Guldemeester, Albert D. M. E. Osterhaus, Armin R. W. Elbers, Roy Slaterus, Menno Hornman, Guus Koch, Ron A. M. Fouchier

**Affiliations:** 1 Department of Viroscience, Erasmus MC, Rotterdam, the Netherlands; 2 Central Veterinary Institute part of Wageningen UR, Lelystad, the Netherlands; 3 Sovon, Dutch Centre for Field Ornithology, Nijmegen, the Netherlands; Justus-Liebeig University Giessen, GERMANY

## Abstract

Avian influenza viruses from wild birds can cause outbreaks in poultry, and occasionally infect humans upon exposure to infected poultry. Identification and characterization of viral reservoirs and transmission routes is important to develop strategies that prevent infection of poultry, and subsequently virus transmission between poultry holdings and to humans. Based on spatial, temporal and phylogenetic analyses of data generated as part of intense and large-scale influenza surveillance programs in wild birds and poultry in the Netherlands from 2006 to 2011, we demonstrate that LPAIV subtype distribution differed between wild birds and poultry, suggestive of host-range restrictions. LPAIV isolated from Dutch poultry were genetically most closely related to LPAIV isolated from wild birds in the Netherlands or occasionally elsewhere in Western Europe. However, a relatively long time interval was observed between the isolations of related viruses from wild birds and poultry. Spatial analyses provided evidence for mallards (*Anas platyrhynchos*) being more abundant near primary infected poultry farms. Detailed year-round investigation of virus prevalence and wild bird species distribution and behavior near poultry farms should be used to improve risk assessment in relation to avian influenza virus introduction and retarget avian influenza surveillance programs.

## Introduction

Avian influenza A virus (AIV) outbreaks may have a high impact on animal health and welfare. Moreover, influenza virus subtypes A(H5N1), A(H7N2), A(H7N3), A(H7N7), A(H7N9), A(H9N2), A(H10N7) and A(H10N8) can form a human health risk as they have been isolated from humans upon exposure to poultry [1–9]. Infection with these subtypes was associated with mild to severe disease in humans. To prevent infection of poultry, virus spread in poultry holdings and transmission to humans, viral reservoirs and transmission routes into poultry holdings need to be identified and characterized.

Wild birds are the reservoir of AIV subtypes H1-H16 (hemagglutinin, HA) and N1-N9 (neuraminidase, NA) [10, 11]. More recently, influenza A virus subtypes H17N10 and H18N11 have been identified in fruit bats [12, 13]. It has been suggested that wild birds, especially waterfowl, are the source of avian influenza outbreaks in poultry [11, 14, 15] as a close genetic relationship of AIV in wild and domestic birds has been documented for several outbreaks [6, 14, 16–18].

Most of the studies that link AIV in poultry and wild birds are based on single highly pathogenic avian influenza (HPAI) H5 or H7 virus outbreaks and limited attention has been paid to the species or temporal and spatial aspects of detection of closely related wild bird viruses. Similarly, most studies that define physical and anthropogenic environmental risk factors associated with poultry have been based on H5 HPAIV outbreaks with no or limited attention paid to wild bird distribution [19, 20]. Wild birds are frequently infected with low pathogenic avian influenza viruses (LPAIV), and LPAIV infections in poultry may often go unnoticed and probably occur more frequently than previously assumed [21–23]. In addition, LPAIV of diverse origin may be ancestral to HPAIV causing outbreaks in poultry [18]. Wild bird species of importance to poultry with respect to AIV infection can be identified based on genetic analyses of their LPAIV, and information on temporal and spatial variation of LPAIV in wild birds can be useful for disease management purposes and for development of targeted surveillance programs.

From 2005 onwards, many countries have implemented or intensified AIV surveillance programs in wild birds and poultry after inter-regional spread of H5N1 HPAIV. These programs aimed at the real-time detection of H5 and H7 viruses as an early warning system for outbreaks in poultry and to provide definitive proof for the role of wild birds in spreading the disease [24, 25]. The AIV surveillance programs in the Netherlands are among the most intensive surveillance programs in the world, encompassing a relatively small surface area with high numbers of water birds and poultry farms.

Here we describe the host species, temporal and spatial aspects of LPAIV detected in poultry and wild birds in the Netherlands between 2006 and 2011. Genetic analyses were performed on LPAIV isolated from poultry and wild birds. In addition, we made an attempt to define wild bird related environmental risk factors of LPAIV introductions into poultry holdings.

## Materials and methods

### Study population poultry

Chicken is the dominant species on poultry farms in the Netherlands (in 2010, 1806/2161, 84%), followed by duck (59/2161, 3%) and turkey (53/2161, 2%). The majority of chicken farms are commercial egg layer farms (1126/2161, 52%) that predominantly keep layers indoors (840/1126, 75%) and to a lesser extend outdoors (286/1126, 25%) [26]. Farms with less than 250 birds were excluded from the analyses. Poultry farms were located throughout the Netherlands with highest poultry farm densities (predominantly chicken layer farms) located in the center and southeastern part of the Netherlands (S1 Fig).

### Study population wild birds

The Netherlands forms an important region for breeding, staging and wintering of wild birds. Over 500 species have been sighted, of which 213 breed in the Netherlands with mallard (*Anas platyrhynchos*) as most common breeding aquatic bird species [27]. Annually at least 130 aquatic bird species winter regularly in the Netherlands [28]. Mallards are distributed more diffuse year round, while Eurasian wigeon (*Anas penelope*) and greater white-fronted geese (*Anser albifrons*) winter in dense groups more locally. Birds were captured manually or using duck decoys, duck traps, clap nets, cannon nets, mist nets or wader funnel traps. The capturing of wild birds was approved by the Dutch Ministry of Economic Affairs based on the Flora and Fauna Act (permit number FF/75A/2009/067). The handling and sampling of wild birds was approved by the Animal Experiment Committee of the Erasmus MC (permit number 122-07-09, 122-08-12, 122-09-20, 122-10-20 and 122-11-31). Sites of wild bird sampling were mainly located in water-rich areas or along main rivers (S1 Fig).

### Influenza A virus surveillance programs poultry

In the Netherlands a serological surveillance program has been initiated in 2003, based on Council directive 2005/94/EC [29], but which is much more intensive compared to the basic program in other European countries: all farms are sampled once a year, but layer farms with outdoor facilities are sampled 4 times per year, and on turkey farms, every production cycle is sampled [25]. This program focuses on the detection of subclinical infection of H5 and H7 LPAIV in poultry, while serving the detection of LPAIV of other subtypes. Clinical surveillance targets the early detection of diseases like avian influenza, supported by the Early Warning System (EWS) based on recommended clinical thresholds [29]. Samples for virus detection were collected if farms tested positive for H5- or H7-specific antibodies within the serological surveillance program, or if AIV infection was suspected based on clinical signs. These samples consisted of oropharyngeal and cloacal swab specimens and/or trachea or lung tissues in case of increased mortality. In this study, farms were considered AIV positive if AIV-specific antibodies were detected in more than one bird per farm and/or if the HA subtype was characterized based on antibodies detected or viruses isolated within the study period (2006–2011).

### Categorization of poultry farms into primary or secondary AIV infected farms

AIV-positive farms of known HA subtype were categorized into most likely infected by wild birds directly (i.e. primary infected farm) or most likely infected as the result of virus spread between farms (i.e. secondary infected farm). Categorization of primary and secondary farms builds on the study of Gonzales and colleagues [23]. In addition, for the purpose of this study a more conservative approach was used based on HA subtype, date of virus or antibody detection and genetic analyses. Genetic analyses suggested—irrespective of farm location—that if the time interval between detections of identical AIV subtypes was more than one year, a new AIV introduction was more likely (this study). Thus, a farm was categorized as primary infected farm (n = 18), if the time interval between current and previous poultry AIV detection of the same subtype was at least one year. A farm was categorized as secondary infected farm (n = 47), if the time interval between current and previous AIV detection of the same subtype was less than one year. If a farm was infected multiple times with different HA subtypes and was listed at least once as primary case, this farm was categorized as primary infected farm. Poultry farms categorized as AIV negative farms consisted of farms that tested AIV negative before and during the course of the study period (2006–2011).

### Antibody detection

Routinely, poultry serum samples collected for AIV-specific antibody detection were analyzed at the Dutch Animal Health Service. Before January 1^st^ 2009, chicken and turkey sera were tested using an indirect AIV-specific ELISA (FlockChek AIV Antibody Test Kit, IDEXX, Hoofddorp, the Netherlands) and duck sera were tested using an in-house developed NP blocking ELISA [30] or directly with the hemagglutination inhibition (HI) assay using H5 and H7 antigens [15]. After January 1^st^ 2009, chicken, turkey and duck sera were tested using a nucleoprotein (NP)-specific multispecies blocking ELISA (bELISA, FlockChek AI MultiS-Screen Antibody Test Kit, IDEXX). If AIV-specific antibodies were detected, AIV subtype was determined using an HI assay and neuraminidase inhibition (NI) assay at the Central Veterinary Institute [15, 31]. AIV subtype could not be determined for some of the AIV NP positive sera due to bad quality and/or insufficient amount of sera.

### Influenza A virus surveillance programs wild birds

In the Netherlands a surveillance program has been initiated in 1998 in which live wild birds were sampled for virus detection. The aim of this program was to detect H5 and H7 HPAIV and LPAIV in wild birds, and to study the epidemiology and evolution of LPAIV of all subtypes. To detect viruses, swab samples were collected from cloaca and from 2006 onwards from both cloaca and oropharynx. Samples were stored in virus transport medium [32] at 4°C for less than a week or at -80°C or -20°C if more than a week until analysis in the laboratory. Birds were considered AIV positive if cloaca and/or oropharynx tested virus positive.

In addition to the sampling of live birds, wild birds found dead were sampled for virus detection since 2006. Data on LPAIV prevalence in dead wild birds was not included in this study.

### Virus detection

Wild bird samples collected for virus detection were analyzed at the Erasmus MC as described previously [32]. In short, RNA was isolated, and analyzed using a reverse transcriptase—polymerase chain reaction assay targeting the matrix gene (M-RT-PCR) on an ABI 7500 machine. Next, M-RT-PCR positive samples (i.e. cycle threshold value <40) were analyzed using a RT-PCR targeting the H5 and H7 gene [6, 32]. Poultry samples collected for virus detection were analyzed at the Central Veterinary Institute in accordance with the Diagnostic Manual of the Council Directive 2005/94/EC [31].

### Virus isolation and characterization

Wild bird M-RT-PCR positive samples were used for virus isolation and characterization as described previously [32]. Briefly, M-RT-PCR positive samples were inoculated in the allantoic cavity of 11-day old embryonated chicken eggs. The allantoic fluid was harvested after two days and AIV was detected using hemagglutination assays with turkey erythrocytes. The HA subtype of the virus isolates was characterized using an HI assay with turkey erythrocytes and hyper-immune rabbit- and ferret antisera raised against 16 HA subtypes (H1-H16). The NA subtype of virus isolates was characterized by PCR and sequencing [33] and identified with the basic local alignment search tool (BLAST) available from GenBank (www.ncbi.nlm.nih.gov). Poultry viruses were isolated and characterized at the Central Veterinary Institute in accordance with the Diagnostic Manual of the Council Directive 2005/94/EC [31].

### Sequence analyses and genetic analyses

Nucleotide sequences of the HA and NA segments of poultry and wild bird LPAIV were obtained. Upon RNA isolation, cDNA was synthesized using the oligonucleotide (5’-AGCAAAAGCAGG-3’). PCR was performed using the AmpliTaq Gold mix (Applied Biosystems, Bleiswijk, the Netherlands). PCR products separated by gel electrophoresis were purified with the QIAquick gel extraction kit (Qiagen, Leusden, the Netherlands). Sequencing was performed on an ABI Prism 3100 using the Big Dye Terminator sequencing kit version 3.1 (Applied Biosystems). Primers specific for the noncoding regions of HA and NA segments were used as described previously (i.e. HA forward primer [5’-AGCAAAAGCAGGGG-3’] and HA reverse primer [5’-AGTAGAAACAAGGGTGGTTT-3’]; NA forward primer [5’-GTTGAAGATGAATCCAAATC-3’] and NA reverse primer [5’-AGTAGAAACAAGGAGTTTTTT-3’]) [33] and additional HA-specific primers that are available on request.

Poultry nucleotide sequences were supplemented with sequences that displayed high sequence identity, selected using BLAST available from GenBank and GISAID EpiFlu (http://www.gisaid.com). For each poultry HA or NA sequence, a maximum of 100 sequences with the highest percentage sequence identity were selected. For each HA and NA subtype, BLAST results were merged and duplicates removed. Identical sequences (100% nucleotide identity) were removed if isolated from the same host species, country and year. Full length and partial sequences were included and the alignments were adjusted manually to include the highest number of sequences in the analysis. Sequences were aligned using MAFFT version 7 (http://mafft.cbrc.jp/alignment/software/). The best-fit model of nucleotide substitution was determined with jModelTest [34]. Phylogenetic maximum likelihood (ML) trees were generated with the PhyML package version 3.1 [35] using the General Time Reversible model of nucleotide substitution with accounting for estimates of invariable sites and the gamma distribution parameter (GTR+I+G) and subtree pruning and regrafting (SPR) searches. The reliability of the phylogenetic groupings of each tree was assessed with a nonparametric bootstrap re-sampling analysis using PhyML. Trees were visualized using the Figtree program, version 1.4.0 (http://tree.bio.ed.ac.uk/software/figtree). Nucleotide sequences generated within this study are online available under the numbers as listed in S1 and S2 Tables.

### Landscape analyses of poultry farms in relation to wild birds

Primary infected, secondary infected and AIV-negative poultry farms were compared with respect to numbers of wild birds sighted near farms and wild bird related landscape characteristics. Number of birds counted was based on systematic annual mid-winter counts in bird count units near farms from 2006 to 2010 and was part of a long-term national bird breeding and wintering monitoring program carried out by Sovon since 1975 [27]. The selected bird species reside in the Netherlands year round (i.e. mallard) or stage during fall/winter only (i.e. Eurasian wigeons and greater white-fronted geese), and have been shown to host AIV [11]. For each farm included in the analysis, the number of birds per species was based on bird counts in one or more counting unit(s) located within 1000-meter radius around the farm. The number of birds per species per farm was extrapolated to the total surface of the circle with radius 1000 meter around the farm from (the bird density per hectare of counting unit)*(surface counting unit within 1000-meter radius around farm). Poultry farms were included in the analysis if at least 10% of the circle with radius 1000 meter was located within bird counting units (i.e. 703 of 2,064 farms, 34%). The numbers of farms included in the analysis were 6 primary infected, 19 secondary infected and 678 AIV-negative farms (total 703 farms).

Landscape characteristics presumably associated with wild bird distribution (i.e. water, forest and farmland) were investigated. The total surface of water (with at least 6 meter in length or width as determined by the topographic basemap), forest and farmland within 100 and within 1000 meter around each farm (n = 2,064) was derived from a Dutch topographic basemap (TOP10NL, www.kadaster.nl) in the program ArcGIS version 10.2.2. The numbers of farms included in the analysis were 18 primary infected, 47 secondary infected and 1999 AIV-negative farms.

### Statistics

Differences in LPAIV subtype distribution between poultry and wild birds were investigated using the Fisher’s exact test using GraphPad Prism 5. Differences in presence or absence of the different wild bird species near poultry farms were compared using the Fisher’s exact test. Wild bird counts and surface of water, forest and farmland near primary infected farms were compared with wild bird counts and surface of water, forest and farmland near secondary infected and AIV-negative poultry farms using the Mann-Whitney test.

## Results

### Avian influenza virus surveillance in wild birds

From 2006 to 2011, 68,637 live birds belonging to 139 species, 40 families and 18 orders were sampled for AIV detection in the Netherlands. Most birds sampled belong to the order *Anseriformes* (mainly ducks, geese and swans; 50,993 birds; 74%) and *Charadriiformes* (mainly gulls and waders; 16,017 birds; 23%). Sampling intensity varied in time and space with the annual cycle of the wild bird species, with general high sampling intensity in water rich areas and during fall migration and winter staging, and low sampling intensity in areas with less surface water and during spring migration and the breeding season (Table 1).

**Table 1:** Avian influenza virus prevalence in wild birds in the Netherlands, 2006–2011. Total number of wild birds sampled for virus detection in time presented per month (A) and per year (B).

A																
**Month**	**Anseriformes**								**Charadriiformes**				**Non-Anseriformes or Charadriiformes**		**Total**	
	**Duck**				**Goose**		**Swan**		**Gull**		**Wader**					
	**Mallard**		**Other duck species**													
	**Sampled**	**Virus (%)**	**Sampled**	**Virus (%)**	**Sampled**	**Virus (%)**	**Sampled**	**Virus (%)**	**Sampled**	**Virus (%)**	**Sampled**	**Virus (%)**	**Sampled**	**Virus (%)**	**Sampled**	**Virus (%)**
**January**	3210	96 (3)	1101	10 (1)	5088	227 (4)	661	12 (2)	2693	3 (0)	2	0 (0)	117	0 (0)	12872	348 (3)
**February**	1363	58 (4)	862	4 (0)	1549	88 (6)	473	1 (0)	1182	7 (1)	1	0 (0)	132	0 (0)	5562	158 (3)
**March**	952	49 (5)	1291	5 (0)	451	6 (1)	66	3 (5)	576	7 (1)	62	0 (0)	129	0 (0)	3527	70 (2)
**April**	614	11 (2)	152	0 (0)	143	1 (1)	66	0 (0)	634	0 (0)	668	2 (0)	88	0 (0)	2365	14 (1)
**May**	557	11 (2)	142	0 (0)	401	0 (0)	2	0 (0)	368	0 (0)	297	0 (0)	61	0 (0)	1828	11 (1)
**June**	742	71 (10)	128	0 (0)	564	0 (0)	0	0 (0)	3075	106 (3)	192	0 (0)	60	0 (0)	4761	177 (4)
**July**	728	26 (4)	139	1 (1)	73	1 (1)	2	0 (0)	2442	270 (11)	57	0 (0)	146	0 (0)	3587	298 (8)
**August**	1161	184 (16)	164	6 (4)	18	0 (0)	1226	3 (0)	83	1 (1)	152	2 (1)	211	0 (0)	3015	196 (7)
**September**	4203	530 (13)	994	72 (7)	29	1 (3)	264	0 (0)	10	0 (0)	160	1 (1)	194	0 (0)	5854	604 (10)
**October**	4375	601 (14)	1325	201 (15)	778	0 (0)	104	0 (0)	60	1 (2)	207	14 (7)	258	0 (0)	7107	817 (11)
**November**	3377	473 (14)	1058	108 (10)	1353	32 (2)	474	1 (0)	882	19 (2)	29	4 (14)	130	1 (1)	7303	638 (9)
**December**	2910	356 (12)	902	71 (8)	4302	292 (7)	456	11 (2)	2185	9 (0)	0	0 (0)	101	0 (0)	10856	739 (7)
**Total**	24192	2466 (10)	8258	478 (6)	14749	648 (4)	3794	31 (1)	14190	423 (3)	1827	23 (1)	1627	1 (0)	68637	4070 (6)
B																
**Year**	**Anseriformes**								**Charadriiformes**				**Non-Anseriformes or Charadriiformes**		**Total**	
	**Duck**				**Goose**		**Swan**		**Gull**		**Wader**					
	**Mallard**		**Other duck species**													
	**Sampled**	**Virus (%)**	**Sampled**	**Virus (%)**	**Sampled**	**Virus (%)**	**Sampled**	**Virus (%)**	**Sampled**	**Virus (%)**	**Sampled**	**Virus (%)**	**Sampled**	**Virus (%)**	**Sampled**	**Virus (%)**
**2006**	4865	347 (7)	2431	52 (2)	4892	71 (1)	1033	10 (1)	2881	16 (1)	531	1 (0)	1124	0 (0)	17757	497 (3)
**2007**	2589	237 (9)	2523	138 (5)	3051	135 (4)	1386	8 (1)	1950	27 (1)	395	10 (3)	200	0 (0)	12094	555 (5)
**2008**	4066	395 (10)	962	67 (7)	1440	44 (3)	1287	0 (0)	1909	199 (10)	298	8 (3)	201	0 (0)	10163	713 (7)
**2009**	3370	231 (7)	775	15 (2)	2334	132 (6)	25	0 (0)	3189	59 (2)	603	4 (1)	59	1 (2)	10355	442 (4)
**2010**	4654	511 (11)	597	111 (19)	1873	149 (8)	25	3 (12)	3633	40 (1)	0	0 (0)	35	0 (0)	10817	814 (8)
**2011**	4648	745 (16)	970	95 (10)	1159	117 (10)	38	10 (26)	628	82 (13)	0	0 (0)	8	0 (0)	7451	1049 (14)
**Total**	24192	2466 (10)	8258	478 (6)	14749	648 (4)	3794	31 (1)	14190	423 (3)	1827	23 (1)	1627	1 (0)	68637	4070 (6)

Influenza A virus prevalence varied in time and space among species. In birds of the order *Anseriformes*, most viruses were detected by M-RT-PCR in mallards (2,466 of 24,192 birds; 10%) and other ducks (478 of 8,258; 6%), and fewer viruses were detected in geese (648 of 14,749 birds; 4%) and swans (31 of 3,794 birds; 1%). In birds of the order *Charadriiformes*, most viruses were detected in gulls (423 of 14,190 birds; 3%), and fewer viruses were detected in waders (23 of 1,827 birds; 1%). In ducks, highest LPAIV prevalence was detected at aggregation sites in fall (August to December, with a maximum of 14% M-RT-PCR positives in October). In geese, highest LPAIV prevalence was detected at staging areas in winter (December to February, with a maximum of 7% M-RT-PCR positives in December). Lowest LPAIV prevalence was detected in spring, when viruses were detected almost exclusively in ducks (April and May, with a minimum of 1% birds M-RT-PCR positive in April). In gull colonies, highest LPAIV prevalence was detected at their breeding sites in summer (June and July, with 11% birds M-RT-PCR positive in July) (Table 1). Of the total of 4,070 M-RT-PCR positive birds, 542 virus isolates were recovered and characterized, yielding an overall recovery rate of 13%. Within the order *Anseriformes*, most viruses were isolated from mallards (n = 250 of 542; 46%), and fewer viruses were isolated from geese (n = 40; 7%), other ducks (n = 20; 4%) and swans (n = 16; 3%). Within the order *Charadriiformes*, most viruses were isolated from gulls (n = 201; 37%), and fewer viruses were isolated from waders (n = 15; 3%).

### Avian influenza virus surveillance in poultry

From 2006 to 2011, all poultry farms in the Netherlands were sampled for AIV-specific antibody detection. Farm sampling frequency varied among poultry types as described previously, with turkeys and outdoor layers sampled more frequently than ducks, indoor layers and broilers [23]. For the different poultry types, timing of sampling was more or less consistent during the year (Table 2, timing of sampling shown for period 2007–2009).

**Table 2:** Avian influenza virus seroprevalence in poultry in the Netherlands, 2007–2009. Total number of poultry farms sampled for antibody detection in time presented per month (A) and per year (B).

A														
**Month**	**Chicken**						**Turkey**		**Duck**		**Mixed**		**Total**	
	**Layer-indoor**		**Layer-outdoor**		**Broiler**									
	**Sampled**	**Antibody (%)**	**Sampled**	**Antibody (%)**	**Sampled**	**Antibody (%)**	**Sampled**	**Antibody (%)**	**Sampled**	**Antibody (%)**	**Sampled**	**Antibody (%)**	**Sampled**	**Antibody (%)**
**January**	217	0 (0)	181	1 (0.6)	277	0 (0)	56	2 (3.6)	36	0 (0)	25	0 (0)	792	3 (0.4)
**February**	201	1 (0.5)	160	0 (0)	225	0 (0)	52	1 (1.9)	30	0 (0)	27	0 (0)	695	2 (0.3)
**March**	261	0 (0)	226	0 (0)	180	0 (0)	65	0 (0)	15	0 (0)	26	0 (0)	773	0 (0)
**April**	230	1 (0.4)	170	0 (0)	195	0 (0)	49	0 (0)	8	0 (0)	18	0 (0)	670	1 (0.1)
**May**	233	1 (0.4)	211	2 (0.9)	299	0 (0)	58	1 (1.7)	6	0 (0)	22	0 (0)	829	4 (0.5)
**June**	232	3 (1.3)	288	2 (0.7)	177	0 (0)	49	0 (0)	4	0 (0)	29	1 (3.4)	779	6 (0.8)
**July**	181	0 (0)	182	0 (0)	292	0 (0)	66	4 (6.1)	3	0 (0)	19	0 (0)	743	4 0.5)
**August**	160	0 (0)	171	3 (1.8)	164	0 (0)	51	2 (3.9)	6	1 (16.7)	15	0 (0)	567	6 (1.1)
**September**	194	0 (0)	209	0 (0)	155	0 (0)	53	2 (3.8)	5	0 (0)	17	0 (0)	633	2 (0.3)
**October**	157	0 (0)	196	0 (0)	165	0 (0)	48	0 (0)	5	1 (20.0)	19	0 (0)	590	1 (0.2)
**November**	203	0 (0)	209	0 (0)	187	0 (0)	49	0 (0)	6	0 (0)	23	2 (8.7)	677	2 (0.3)
**December**	225	1 (0.4)	279	1 (0.4)	159	0 (0)	49	0 (0)	34	1 (2.9)	37	1 (2.7)	783	4 (0.5)
**Total**	2494	7 (0.3)	2482	9 (0.4)	2475	0 (0)	645	12 (1.9)	158	3 (1.9)	277	4 (1.4)	8531	35 (0.4)
B														
**Year**	**Chicken**						**Turkey**		**Duck**		**Mixed**		**Total**	
	**Layer-indoor**		**Layer-outdoor**		**Broiler**									
	**Sampled**	**Antibody (%)**	**Sampled**	**Antibody (%)**	**Sampled**	**Antibody (%)**	**Sampled**	**Antibody (%)**	**Sampled**	**Antibody (%)**	**Sampled**	**Antibody (%)**	**Sampled**	**Antibody (%)**
**2007**	903	3 (0.3)	751	3 (0.4)	761	0 (0.0)	254	8 (3.1)	51	1 (2.0)	100	1 (1.0)	2820	16 (0.6)
**2008**	843	2 (0.2)	889	1 (0.1)	859	0 (0.0)	214	1 (0.5)	51	2 (3.9)	90	2 (2.2)	2946	8 (0.3)
**2009**	748	2 (0.3)	842	5 (0.6)	855	0 (0.0)	177	3 (1.7)	56	0 (0.0)	87	1 (1.1)	2765	11 (0.4)
**Total**	**2494**	**7 (0.3)**	**2482**	**9 (0.4)**	**2475**	**0 (0.0)**	**645**	**12 (1.9)**	**158**	**3 (1.9)**	**277**	**4 (1.4)**	**8531**	**35 (0.4)**

Influenza A virus seroprevalence varied between poultry types [23] and in time. Highest seroprevalence was detected on turkey and duck farms, followed by mixed, outdoor layer farms and indoor layer farms (Table 2). No AIV-specific antibodies were detected on broiler farms. Most AIV-seropositive farms were detected from May until August with 20 of 35 seropositive cases (57%) from 2007 to 2009 (Table 2).

From 2006 to 2011, in total 82 poultry farms (with unique address) tested positive for AIV and/or antibodies. Of the 82 AIV sero- and/or virus positive poultry farms, 16 virus isolates were obtained. Most virus isolates were obtained from chickens (11 of 16), fewer from turkeys (5 of 16) and none from ducks (Table 3). The HA subtype of the viruses that circulated on 65 of 82 AIV positive poultry farms was identified. A single HA subtype was detected on 63 poultry farms, two different HA subtypes were detected on two poultry farms and four different HA subtypes were detected on one single poultry farm, resulting in 70 HA subtypes on 65 poultry farms. The NA subtype of the viruses that circulated on 29 of 82 AIV positive poultry farms was identified. A single NA subtype was detected on 27 poultry farms, two different NA subtypes were detected on one poultry farm and three different NA subtypes were detected on one poultry farm, resulting in 32 NA subtypes on 29 poultry farms.

**Table 3:** The total number of avian influenza viruses isolated from poultry in the Netherlands between 2006 and 2011 with their genetically closest relatives based on genetic analyses of the hemagglutinin and neuraminidase gene segment.

Poultry LPAI virus			Closest relative of poultry LPAI virus				time interval (days)	sequence identity (%)	length sequence (nt)
Name	Location	Date	Segment	Name	Location	Date			
**A/Ty/Netherlands/06001571/06 (H6N5)**^2^	Dinteloord	16-Jan-2006	HA	A/White-Fronted Goose/Netherlands/1/2006 (H6N2)	Oud-Alblas, NL	14-Jan-2006	2	0,996	1576
			NA	A/Mallard/Switzerland/WV4060167/2006 (H3N5)	Switzerland	15-Dec-2006	325	0,987	1310
**A/Ch/Netherlands/06022003/06 (H7N7)** ^2^	Voorthuizen	1-Aug-2006	HA	A/Mallard/Netherlands/60/2008 (H7N1)	Wieringen, NL	15-Oct-2008	806	0,993	1560
			NA	A/Mallard/Sweden/5944/2005 (H7N7)	Ottenby, Sweden	23-Nov-2005	252	0,987	1238
**A/Ty/Netherlands/07016245/07 (H1N5)** ^2^	Weert	22-Jun-2007	HA	A/Bewick's swan/Netherlands/1/2007 (H1N5)	Friesland, NL	5-Jan-2007	168	0,988	1587
			NA	A/Black-backedGull/Netherlands/1/2006 (H4N5)	Schiermonnikoog, NL	14-Feb-2006	493	0,985	1310
**A/Ty/Netherlands/09006938/09 (H10N7)** ^1^	Deurne	16-Apr-2009	HA	A/Mallard/Netherlands/53/2008 (H10N7)	Wieringen, NL	2-Oct-2008	196	0,993	1571
			NA	A/Mallard/Netherlands/82/2008 (H7N7)	Oudeland van Strijen, NL	17-Dec-2008	120	0,997	1238
**A/Ch/Netherlands/10007882/10 (H7N4)** ^2^	Deurne	16-May-2010	HA	A/Mallard/Netherlands/60/2008 (H7N1)	Wieringen, NL	15-Oct-2008	578	0,987	1560
			NA	A/Ch/Netherlands/10009401/10 (H8N4) ^2^	Hiaure, NL	4-Jun-2010	19	0,989	1345
**A/Ch/Netherlands/10008427/10 (H10N7)** ^1^	Drachtstercompagnie	20-May-2010	HA	A/Mallard/Netherlands/67/2008 (H10N7)	Oud-Alblas, NL	13-Dec-2008	523	0,992	1571
			NA	A/Mallard/Netherlands/74/2008 (H10N7)	Oud-Alblas, NL	13-Dec-2008	523	0,991	1238
**A/Ch/Netherlands/10010413/10 (H6N1)** ^1^	Idsegahuizum	21-May-2010	HA	A/Mallard/Netherlands/18/2010 (H6N8)	Oud-Alblas, NL	3-Sep-2010	105	0,99	1576
			NA	A/Mallard/Bavaria/185-26/2008 (H1N1)	Bavaria, Germany	22-Sep-2008	606	0,987	1306
**A/Ch/Netherlands/10009401/10 (H8N4)** ^2^	Hiaure	4-Jun-2010	HA	A/Ch/Netherlands/11004004/11 (H8N4) ^1^	Vreeland, NL	9-Mar-2011	278	0,984	1644
			NA	A/Ch/Netherlands/10007882/10 (H7N4) ^2^	Deurne, NL	16-May-2010	19	0,989	1345
**A/Ch/Netherlands/10020245/10 (H9N2)** ^1^	Pijnacker	7-Dec-2010	HA	A/Duck/Italy/260/2004 (H9N8)	Italy	1-Jan-2004*	2532	0,969	1588
			NA	A/Mallard/Netherlands/7/2007 (H4N2)	Krimpen aan den IJssel, NL	27-Sep-2007	1167	0,977	1284
**A/Ch/Netherlands/11004004/11 (H8N4)** ^1^	Vreeland	10-Mar-2011	HA	A/Mallard/Sweden/99377/2009 (H8N4)	Ottenby, Sweden	3-Sep-2009	553	0,989	1644
			NA	A/Mallard/Sweden/100546/2009	Ottenby, Sweden	22-Oct-2009	503	0,991	1345
**A/Ch/Netherlands/11004875/11 (H7N1)** ^1^	Schore	24-Mar-2011	HA	A/Mallard/Poland/446/09 (H7N7)	Pomeranian Voivodeship, Poland	27-Dec-2009	452	0,996	1560
			NA	A/Mallard/Netherlands/51/2010 (H1N1)	Oud-Alblas, NL	3-Dec-2010	111	0,995	1306
**A/Ch/Netherlands/11008327/11 (H7N7)** ^2^	Kootwijkerbroek	12-May-2011	HA	A/Ty/Netherlands/11011530/2011 (H7N7) ^2^	Creil, NL	26-Jun-2011	45	0,998	1560
			NA	A/Ty/Germany/R1775/2011 (H7N7)	Germany	1-Jan-2011*	131	0,995	1238
				A/Ch/Germany/R1801/2011 (H7N7)	Germany	1-Jan-2011*	131	0,995	1238
**A/Ch/Netherlands/11009919/11 (H1N1)** ^1^	Stolwijk	30-May-2011	HA	A/White-fronted Goose/Netherlands/4/2011 (H1N1)	Lith, NL	17-Jan-2011	133	0,987	1587
			NA	A/White-fronted Goose/Netherlands/4/2011 (H1N1)	Lith, NL	17-Jan-2011	133	0,999	1306
**A/Ch/Netherlands/11011326/11 (H7N7)** ^2^	Creil	22-Jun-2011	HA	A/Ty/11011530/Netherlands/2011 (H7N7) ^2^	Creil, NL	26-Jun-2011	4	0,999	1560
			NA	A/Ty/11011530/Netherlands/2011 (H7N7) ^2^	Creil, NL	26-Jun-2011	4	0,998	1238
**A/Ty/Netherlands/11011530/11 (H7N7)** ^2^	Creil	26-Jun-2011	HA	A/Ch/Netherlands/11011326/2011 (H7N7) ^2^	Creil, NL	22-Jun-2011	4	0,999	1560
			NA	A/Ch/Netherlands/11011326/2011 (H7N7) ^2^	Creil, NL	22-Jun-2011	4	0,998	1238
**A/Ty/Netherlands/11015452/11 (H9N2)** ^1^	Deurne	31-Aug-2011	HA	A/Teal/Finland/10529/2010 (H9N2)	Söörmarkku, Finland	5-Oct-2010	330	0,985	1588
			NA	A/Mallard/Sweden/99820/2009 (H11N2)	Ottenby, Sweden	27-Sep-2009	703	0,991	1284

^1^ = primary infected farm;

^2^ = secondary infected farm, based on categorization of poultry farms as applied in this study.

HA = hemagglutinin, NA = neuraminidase, NL = the Netherlands, Ty = turkey, Ch = chicken,

* = exact collection date not available.

### Prevalence of influenza A virus HA subtypes in poultry and wild birds

**Fig 1:**
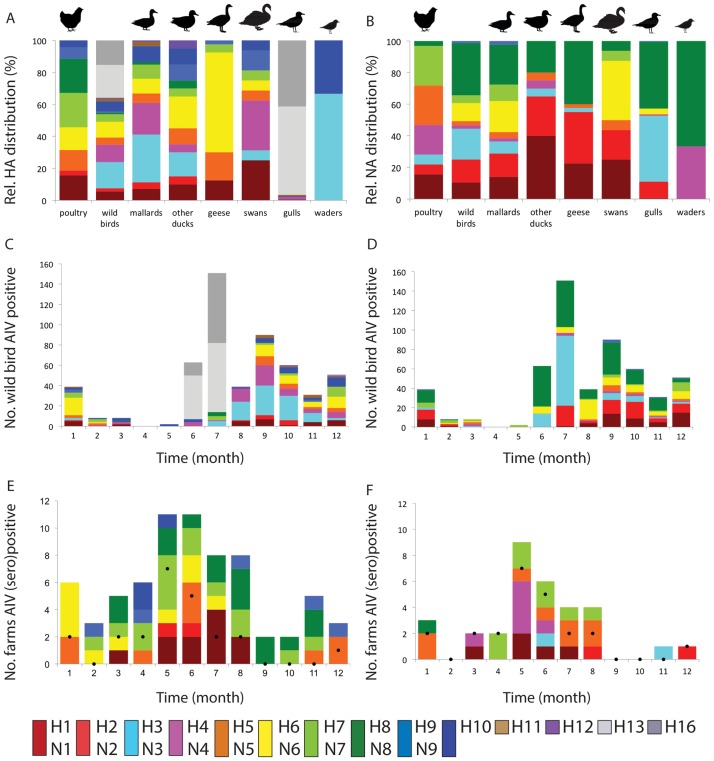
Avian influenza virus subtype distribution in wild birds and poultry, the Netherlands, 2006–2011. Subtype distribution shown for poultry and wild bird species for the hemagglutinin, HA (A) and neuraminidase, NA (B). Distribution based on 70 poultry cases (70 HA and 32 NA known) and 542 wild bird virus isolates (i.e. 250 mallards, 20 other ducks, 40 geese, 16 swans, 201 gulls and 15 waders). Subtype distribution in wild birds in time shown for the HA (C) and NA (D) was based on virus isolates. Subtype distribution in poultry in time shown for the HA (E) and NA (F) was based on antibody detection and/or virus isolation. Black dots indicated number of virus positive farms per month.

In poultry, the most frequently detected HA subtypes were H7 (21%) and H8 (21%), followed by H1 (16%), H5 (13%), H6 (14%), H9 (7%), H10 (4%) and H2 (3%) (Fig 1A, Table 4, S3 Table). Viruses of the H1 subtype were primarily detected in turkeys (8 of 11), even though only 2% of Dutch poultry farms house turkeys. Due to follow-up investigation of all AIV-(sero)positive poultry farms for H5- and H7 AIV or antibodies, HA subtypes other than H5 or H7 may be under represented among the HA subtypes detected in poultry. In wild birds, H13 (20%), H3 (16%), H16 (15%) and H4 (11%) were the most abundantly isolated HA subtypes, followed by H6 (10%), H1 (6%), H10 (6%), H5 (5%), H7 (5%), H2 (2%), H11 (2%), H8 (1%), H9 (1%) and H12 (1%). Viruses of the H3 and H4 subtype were primarily isolated from dabbling ducks (128 of 147; 87% of all H3 and H4 viruses isolated), while H13 and H16 subtypes were exclusively isolated from gulls (194 of 194, 100% of all H13 and H16 viruses isolated). HA subtype diversity based on viruses detected in poultry was highest in May and June (Fig 1E), and in wild birds in September to January (Fig 1C). Of the HA subtypes detected in poultry, H5 and H6 were significantly more frequently isolated from geese (H5: geese 7 of 40; 18% versus all wild birds combined 25 of 542; 5%, P < 0.01, Fisher’s exact test. H6: geese 25 of 40; 63% versus all wild birds combined 53 of 542; 10%, P < 0.01, Fisher’s exact test, S3 Table), while H10 was significantly more frequently isolated from waders (waders 5 of 15; 33% versus all wild birds combined 34 of 542; 6%, P < 0.01, Fisher’s exact test). All HA subtypes isolated from geese (n = 5) were detected in poultry (n = 8) (Fig 1, S3 Table).

**Table 4:** Avian influenza virus HA and NA subtype combinations detected in wild birds and poultry, the Netherlands, 2006 to 2011. For wild birds, subtypes were based on virus isolates. For poultry, subtypes were based on antibody detection, virus detection and/or virus isolation. Numbers refer to wild birds and numbers between brackets refer to poultry farms. Subtype combinations indicated with an asterisk were significant more frequently detected in poultry than in wild birds, with * = P <0.05 and ** = P <0.01 (Fisher’s exact test).

Subtype	N1	N2	N3	N4	N5	N6	N7	N8	N9	Nx	Total
**H1**	26 (1)	1	1	1	1(6)**					(4)	30 (11)
**H2**		3	8(1)							(1)	11 (2)
**H3**	1	16	1			11		59	1		89 (0)
**H4**		5			7	41		5			58 (0)
**H5**		16	8			1				(9)	25 (9)
**H6**	11(3)	11			3(2)			28(1)		(4)	53 (10)
**H7**	13(1)	1	4(1)	1(2)*			6(5)**	1		(6)	26 (15)
**H8**	1			4(4)**						(11)	5 (15)
**H9**		5(2)								(3)	5(5)
**H10**	3			6		4	21(3)				34 (3)
**H11**	2							1	6		9 (0)
**H12**					3						3 (0)
**H13**		21	3			5		82			111 (0)
**H16**			80					3			83 (0)
**Total**	57 (5)	79 (2)	105 (2)	12 (6)	14 (8)	62 (0)	27 (8)	179 (1)	7 (0)	(38)	542 (70)

No H5 or H7 HPAIV were detected in poultry or wild birds within the study period. In addition to virus isolations, in wild birds H5 and H7 viruses were detected using HA-specific RT-PCR assays. Of 4,070 M-RT-PCR positive birds, 96 birds tested positive for H5 viruses and 36 birds tested positive for H7 viruses. H5 viruses were detected from August until March with most H5 virus detections in October (26 of 96, detected in October in 6 of 6 years). H7 viruses were detected from July until April with most H7 virus detections in December (12 of 36, detected in December in 3 of 6 years). Of M-RT-PCR positive birds, H5 viruses were detected in swans significantly more frequently (3 of 31; 10%) than in all wild birds combined (96 of 4,070; 2%) (P <0.05, Fisher’s exact test), whereas in gulls H5 viruses were detected significantly less frequently (1 of 423; 0.2%) by H5-specific RT-PCR (P < 0.01, Fisher’s exact test). Furthermore, H5 viruses were detected in mallards (62 of 2,466; 3% of M-RT-PCR positive birds), other ducks (9 of 478; 2%) and geese (21 of 648; 3%). No H5 viruses were detected in M-RT-PCR positive waders (0 of 23; 0%). Of M-RT-PCR positive birds, H7 viruses were detected in mallards (17 of 2,466; 1%), other ducks (6 of 478; 1%) and geese (3 of 648; 0.5%). No H7 viruses were detected in swans (0 of 31, 0%), gulls (0 of 423; 0%) or waders (0 of 23; 0%) by H7-specific RT-PCR.

### Prevalence of influenza A virus NA subtypes in poultry and wild birds

In poultry, N5 (25%), N7 (25%) and N4 (19%) were most frequently detected, followed by N1 (16%), N2 (6%), N3 (6%) and N8 (3%) (Fig 1B, Table 4, S3 Table). Viruses of the N5 subtype were more frequently detected in turkeys, and in most cases linked to H1 that was also mostly detected in turkeys. In wild birds, N8 (33%) and N3 (19%) were most frequently detected, followed by N2 (15%), N1 (11%), N6 (11%), N7 (5%), N5 (3%), N4 (2%) and N9 (1%). Viruses of the N3 and N8 subtype were most frequently isolated from gulls and combined with H13 or H16 subtype. NA subtypes detected in poultry differed from the NA subtypes as detected in wild birds (Fig 1, S3 Table), but all NA subtypes isolated from other duck species, geese and waders were detected in poultry. Highest NA subtype diversity based on virus detection was detected in June in poultry (Fig 1F), and in September to January in wild birds (Fig 1D).

### Difference in influenza A virus HA/NA combinations in poultry and wild birds

The subtype of AIV that circulated on poultry farms was characterized for 32 out of 82 AIV infected poultry farms, resulting in 13 different HA/NA combinations (Table 4). In poultry, most frequently detected HA/NA combinations were H1N5 (6 of 32, 19%), H7N7 (5 of 32, 16%) and H8N4 (4 of 32, 12%) (Table 4). These subtype combinations and H7N4 were significantly more frequently detected in poultry than in wild birds (P < 0.05, Fisher’s exact test). Part of detections of these subtypes (i.e. H1N5 and H6N5) were epidemiologically linked (e.g. described contact between farms during introduction or infectious period potentially explaining spread between farms). In wild birds most frequently isolated HA/NA combinations were H13N8 (82 of 542, 15%), H16N3 (80 of 542, 15%), H3N8 (59 of 542, 11%) and H4N6 (41 of 542, 8%).

All HA/NA combinations detected in poultry were detected in wild birds in the Netherlands. Nearly all HA/NA combinations detected in poultry (n = 13) were as well detected in ducks (12 of 13), part of the HA/NA combinations detected in poultry were detected in geese (6 of 13) or swans (6 of 13) and none of the HA/NA combinations were detected in gulls (0 of 13) or waders (0 of 13). However, the HA/NA combinations that were detected in poultry were relatively more frequently isolated from geese (24 of 40; 63%) and swans (9 of 16; 56%) than from ducks (95 of 270; 35%), and were not isolated from gulls (0 of 201; 0%) or waders (0 of 15; 0%).

The majority of HA/NA combinations as detected in poultry in this study were found in studies done previously in Europe in mallards (12 of 13 [36]) and in the US in ducks (7 of 13 [37]; 10 of 13 [38]; 11 of 13 [39]). No large-scale studies have been published on HA/NA combinations in geese or swans. In contrast to our findings where no HA/NA combinations as detected in poultry were found in gulls or waders, 10 of 13 HA/NA combinations detected in poultry in this study have been detected in shorebirds in the US [39].

### Genetic links of poultry and wild bird influenza A viruses

A total of 16 LPAIV isolated from poultry between 2006 and 2011 (Table 3) were included in the genetic analyses. For most poultry HA and NA nucleotide sequences, the closest relatives as identified by BLAST and phylogeny were wild bird LPAIV (11 of 16 poultry HA and NA genes, Table 3, S2 Fig). Poultry LPAIV that were most closely related to other poultry LPAIV were of subtypes less commonly or rarely detected in wild birds within the study period (i.e. H7, H8, N4 and N7) (Table 4, S3 Table).

Based on genetic analyses of the HA and NA segments, the majority of poultry LPAIV isolates were most closely related to HA and NA of two different LPAIV (Table 3), with one poultry LPAIV isolate genetically most closely related to a single wild bird LPAIV isolate (i.e. A/Ch/Netherlands/11009919/11 [H1N1] and A/White-fronted Goose/Netherlands/4/2011 [H1N1]). A second poultry LPAIV was genetically most closely related to H10N7 LPAIV isolated from two mallards sampled at one site on one day (i.e. A/Ch/Netherlands/10008427/10 (H10N7) with HA of A/Mallard/Netherlands/67/2008 (H10N7) and NA of A/Mallard/Netherlands/74/2008 (H10N7)).

Although all poultry HA and NA subtypes were detected in viruses isolated from wild birds in the Netherlands within the study period, several of the 16 poultry isolates were genetically most closely related to LPAIV isolated from wild birds sampled outside the Netherlands but within Western Europe (Table 3). The time interval between detection of genetically closely related LPAIV varied considerably, from 2 days until 2,532 days (Table 3). This time interval was shorter for more common wild bird HA subtypes like H6 (2 to 105 days) than for more rarely detected wild bird HA subtypes like H9 (805 to 2,532 days). The time interval for more common wild bird NA subtypes like N1 (111 to 606 days) and N2 (703 to 1,167 days) did not differ from more rarely detected wild bird NA subtypes like N7 (245 to 523 days) and N5 (325 to 493 days) (Tables 3 and 4).

### Landscape analyses of poultry farms in relation to wild birds

Mallards were observed significantly more frequently near poultry farms (675 of 703 farms; 96%) than Eurasian wigeons (490 of 703; 70%, P < 0.0001, Fisher’s exact test) or greater white-fronted geese (512 of 703; 73%, P < 0.0001, Fisher’s exact test). However, presence of mallards, Eurasian wigeons or greater white-fronted geese did not differ significantly between primary infected, secondary infected or AIV-negative farms (P > 0.05, Fisher’s exact test). Despite the fact that mallards were observed more frequently within 1000 meter around poultry farms, Eurasian wigeons and greater white-fronted geese were, if observed, counted in significantly higher numbers than mallards (mean mallards 63 birds per farm, mean Eurasian wigeons 154 birds per farm (P < 0.0001, Mann-Whitney test) and greater white-fronted geese 226 birds per farm (P < 0.05, Mann-Whitney test).

**Fig 2:**
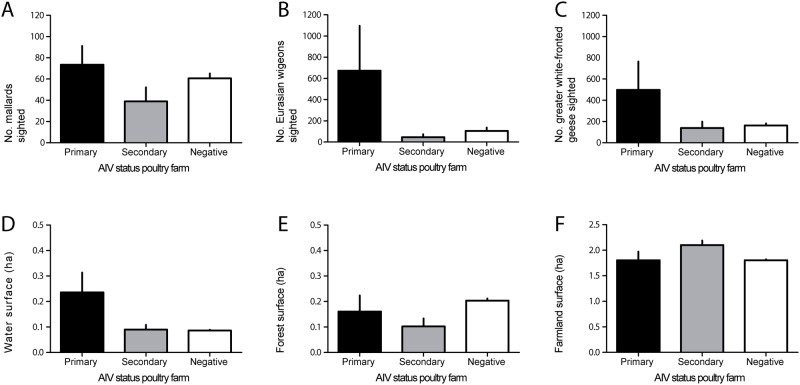
Wild bird distribution and environmental characteristics near primary infected, secondary infected and avian influenza virus negative poultry farms in the Netherlands. For poultry farms located near bird monitoring areas (n = 703: consisting of 6 primary infected, 19 secondary infected and 678 AIV negative farms) number of mallards (A), Eurasian wigeons (B) and greater white-fronted geese (C) within 1000 meters around farms (mean±SE) was investigated. For all poultry farms (n = 2,064: consisting of 18 primary infected, 47 secondary infected and 1,999 AIV negative farms) surface of water (D), forest (E) and farmland (F) within 100 meters around farms (mean±SE) was investigated. Black bars indicates primary infected farms, grey indicates secondary infected farms and white indicates AIV negative farms. Asterisk indicates statistically significant differences (P < 0.05, Mann-Whitney test).

Overall, mean number of mallards counted near primary infected farms (n = 73) was significantly higher than near secondary (n = 39, P < 0.05, Mann-Whitney test) or near AIV-negative farms (n = 61, P < 0.05, Mann-Whitney test) (Fig 2). Mean number of Eurasian wigeons and greater white-fronted geese was higher near primary infected farms than near secondary infected or AIV-negative farms, however not significantly (respectively 673, 45, 104 Eurasian wigeons and 499, 139, 163 greater white-fronted geese) (P > 0.05, Mann-Whitney test) (Fig 2). Water surface within 100 meter around a poultry farm was higher near primary infected farms (0.24 ha, n = 18) than near secondary infected (0.09 ha, n = 47, P > 0.05, Mann-Whitney test) or than AIV-negative farms (0.09 ha, n = 1999, P > 0.05, Mann-Whitney test), however not significantly (Fig 2). Surface of forest or farmland within 100 meter around poultry farm did not differ significantly between primary, secondary and AIV-negative poultry farms (P > 0.05, Mann-Whitney test) (Fig 2). Water, farmland and forest surface within 1000 meter around poultry farms did not differ significantly between primary infected, secondary infected and AIV-negative farms (data not shown) (all P > 0.05, Mann-Whitney test).

In addition, when the data on birds sighted near farms was analyzed by indoor layer farm (i.e. 3 primary infected, 3 secondary infected, 212 AIV-negative farms) and outdoor layer farm (i.e. 3 primary infected, 10 secondary infected and 88 AIV-negative farms) subgroups the mean number of mallards, Eurasian wigeon and greater white-fronted geese was—similar to when data was analyzed with the different poultry production types merged—highest near primary infected farms, however not significantly higher than AIV-negative farms (P > 0.05, Mann-Whitney test). When the data on environmental characteristics was analyzed by indoor layer farm (i.e. 5 primary infected, 11 secondary infected and 761 AIV-negative farms) and outdoor layer farm (i.e. 7 primary infected, 24 secondary infected and 295 AIV-negative farms) subgroups the water surface within 100 meter around an outdoor layer farm was significantly higher near primary infected farms (0.36 ha, n = 3) than near secondary infected outdoor layer farms (0.11 ha, n = 10, P < 0.05, Mann-Whitney test) or than near AIV-negative outdoor layer farms (0.07 ha, n = 88, P < 0.0001, Mann-Whitney test). Surface of forest or farmland within 100 meter around indoor layer or outdoor layer farms did not differ significantly between primary, secondary and AIV-negative farms (P > 0.05, Mann-Whitney test). Farms of poultry production types other than indoor layers or outdoor layers (i.e. turkeys, ducks, mixed and broilers) could not be evaluated at the production type level as these poultry production types were not represented in all 3 categories: primary infected, secondary infected and AIV-negative farms.

## Discussion

Within this 6-year study in the Netherlands, LPAIV subtype distribution differed between poultry and wild birds and indicated apparent differences in host susceptibility to LPAIV subtypes and lineages. LPAIV of some subtypes (i.e. H1, H5, H7, H8, H9, N4, N5 and N7) were significantly more frequently detected in poultry than in wild birds, while LPAIV of other subtypes (i.e. H3, H4, H13, H16, N6 and N8) were significantly more frequently detected in wild birds than in poultry. Given the significant differences, random subtype distribution in wild birds and poultry seems unlikely. Within this study, poultry LPAIV subtype combinations were most frequently detected within wild geese (27 of 40 virus isolates, 67%), followed by swans and ducks. Whether geese acted as so called bridge species for introduction of LPAIV into poultry farms, or whether they are susceptible to the same LPAIV subtypes as chickens and turkeys but do not act as bridge species, or whether they were infected with LPAIV strains that have a broader host range in general (e.g. H6 viruses [40])—and therefore are more likely to infect poultry—needs to be determined. Low pathogenic avian influenza viruses of the H8 and H9 subtype as detected in poultry (e.g. H8N1, H8N4, H9N2) were detected in ducks and geese in the Netherlands rarely, and exclusively outside LPAIV peak prevalence in autumn. Similar to our findings, H8 and H9 viruses were very uncommon in mallards in Sweden, and if detected then most often of the subtype H8N4 or H9N2 [36]. H8 and H9 viruses were very uncommon in ducks in the US [38], but relatively common in waterfowl and shorebirds in Australia [41]. Remarkably, none of the common HA subtypes in *Anseriformes*, like H3 and H4 were detected in poultry. A study done previously on the effect of season on the incidence of LPAIV in turkeys showed that these virus subtypes were isolated from turkeys to a limited extent [21]. Potential explanations for differences in host susceptibility may be related to the virus strain (e.g. virus tropism, replication, immune evasion) and/or related to modes of transmission (e.g. respiratory, uptake fecal material, water-dependent) [42–47]. It may be worthwhile to experimentally test a variety of LPAIV subtypes and lineages in poultry, to investigate if particular viruses are indeed more prone to cause infections in chickens and turkeys. In addition, observed difference in LPAIV subtype distribution between poultry and wild birds may partly be explained by the considerable spatial discordance between sampling locations of wild birds and locations of poultry farms (S1 Fig).

In this study, a long time-interval between LPAIV detection in wild birds and poultry was detected suggesting that the conditions for LPAIV introduction into poultry rely on more than just LPAIV peak prevalence in wild birds. The long time-interval between LPAIV detection in wild birds and poultry may be explained by the variation between and within seasons in LPAIV subtype predominance as described for ducks [36, 38, 39, 48, 49], suggesting an inter-annual and within season variation in the subtypes that could potentially spill over to poultry. In addition, despite LPAIV peak prevalence in e.g. wild ducks in fall and winter, LPAIV may not reach farms at that time of year due to foraging and aggregation behavior of ducks elsewhere. In addition to wild bird behavior and distribution, seasonal changes in poultry behavior in outdoor farms potentially affect the exposure to LPAIV. It has been suggested that outdoor layers spend more time outside when precipitation is low. In the Netherlands, spring is the driest season, which may explain increased LPAIV detections in outdoor poultry at the end of spring and early summer, however published data supporting this is lacking. Furthermore, a specific wild bird species may be at the source of introduction into poultry that is currently not identified. Most sampling activities in live wild birds focus on mallards—and high LPAIV prevalence and diversity has been demonstrated in this species—whereas a different avian species may be infected with LPAIV more relevant to poultry. Also, the spatial scale at which the surveillance program is being carried out may affect the time interval between LPAIV detection in wild birds and poultry. For instance, a relatively short time interval (i.e. 6–8 weeks) was reported for LPAIV detection in sentinel ducks and domestic turkeys in a 4-year study in Minnesota, USA [21]. Thus, in addition to LPAIV prevalence and LPAIV characterization in both poultry and wild birds, data on wild bird species distribution and behavior directly near poultry farms year-round would be valuable information to define risk species and periods of AIV introduction.

The majority of poultry LPAIV isolates most likely originated from independent introductions from wild birds, but such independent wild bird origin can not be inferred with confidence for some HA and NA subtype viruses, i.e. H7, H8 and N4. The long time interval between the detection of poultry LPAIV and their most closely related LPAIV in wild birds as detected by genetic analyses of HA and NA segments, may indicate that the wild bird surveillance program as implemented in the Netherlands is of insufficient intensity or focus if it were to provide “early warning signals” for outbreaks in poultry. Also, a relatively large proportion of poultry HA or NA segments were most closely related to LPAIVs detected outside the Netherlands, in most cases Western European countries. To better facilitate studies like this one, organisations involved in avian influenza surveillance programs should be encouraged to release LPAIV sequence data—for poultry and wild bird viruses—more routinely into public databases.

In our study, mallards were observed more frequently near poultry farms than Eurasian wigeons or greater white-fronted geese. This is not surprising, given the more continuous distribution of mallards in winter, whereas wigeons and geese tend to aggregate in large flocks locally. Consequently, if wigeons or geese were found, the number of birds was much higher than for mallards. However, of these three bird species, only mallards were sighted in statistically significantly higher numbers near AIV primary infected farms. Increased water surface directly surrounding the poultry farms was associated with AIV primary infected farms, however not statistically supported. When farm data with respect to observed wild birds and environmental characteristics near farms was evaluated based on farm type (i.e. indoor layers and outdoor layers), water surface directly surrounding AIV primary infected outdoor layer farms was significantly higher than water surface directly surrounding AIV secondary infected outdoor layer farms and than water surface directly surrounding AIV-negative outdoor layer farms, nevertheless these findings need to be interpreted cautiously as sample size is extremely small. Although annual bird counts cover a large part of the Netherlands, the counts were skewed towards water rich and poultry poor areas and therefore a minority of farms was covered by these counts. Detailed case-control studies on year-round wild bird distribution and behavior near AIV-positive and -negative poultry farms may identify wild bird related risk factors in relation to AIV introduction.

Despite relatively intensive avian influenza surveillance programs established in the Netherlands, it is still difficult to link wild bird and poultry LPAIV with certainty in time and space. To ultimately better target wild bird surveillance programs, more fundamental knowledge is needed on the susceptibility of host species to different LPAIV and on the routes of virus introduction into farms. Therefore, more detailed multi-disciplinary studies are needed that include year-round data on virus prevalence and wild bird distribution and behavior near poultry farms during day and night, and data on poultry like timing of seroconversion (e.g. based on measuring AIV-specific antibodies in eggs from layers), age at sampling, seasonality of placing new flocks, biosecurity and presence of other disease(s). In addition, metagenomics on feces from poultry and different wild bird species may be a helpful tool to identify bridge-species. Furthermore, virus isolation and virus sequencing of both wild birds and poultry is crucial to identify potential bridge and/or reservoir wild bird species, as well as to support experimental studies on the identification of viruses more prone to cause infections in chickens and turkeys. Improved knowledge on host species and routes into poultry farms, will facilitate better targeted poultry and wild bird surveillance programs. Our findings establish that evaluation of the design of current large-scale AIV surveillance programs in wild birds and poultry is needed to improve for risk assessment of AIV introduction and minimize the costs.

## Supporting information

S1 Fig Distribution of poultry farms (A) and sites of wild bird sampling (B) within the Netherlands, 2006 to 2011. Black indicates poultry farms or wild birds that tested positive for avian influenza viruses, grey indicates poultry farms or wild birds that tested negative for avian influenza viruses. The figure is derived from TOP10NL, Kadaster, Basisregistratie Topografie (BRT), licensed under CC-BY-4.0.
(PDF)

S2 Fig Maximum likelihood trees of influenza A virus HA and NA subtypes as detected in poultry, the Netherlands, 2006–2011. H1 (A), H6 (B), H7 (C), H8 (D), H9 (E), H10 (F), N1 (G), N2 (H), N4 (I), N5 (J) and N7 (K). Red indicates influenza viruses isolated from poultry in the Netherlands within this study period and blue indicates the genetically closest influenza virus isolated from wild birds.
(PDF)

S1 Table The Low Pathogenic Avian Influenza Viruses (LPAIV) and the accession numbers of the segments used in this study as listed in online databases GenBank and GISAID EpiFlu. (PDF)

S2 Table Details of the Low Pathogenic Avian Influenza Viruses (LPAIV) sequences downloaded from the GISAID EpiFlu database. We gratefully acknowledge the authors, originating and submitting laboratories of the sequences from the GISAID EpiFlu Database on which this research is based. All submitters may be contacted directly via the GISAID website.
(PDF)

S3 Table Avian influenza virus hemagglutinin and neuraminidase subtype distribution among poultry and wild bird species, the Netherlands, 2006–2011. Number and percentage (between brackets) of hemagglutinin (A) and neuraminidase (B) subtypes are shown for poultry and wild bird species. Poultry subtypes are shown for primary and secondary cases (i.e. all combined) and separate for primary cases only (i.e. primary cases). Subtypes indicated with an asterisk were significantly more or less frequently detected in the corresponding group than in all wild birds combined, with * = P <0.05 and ** = P <0.01 (Fisher’s exact test).
(PDF)
