# Supplementary material for: Discordant detection of avian influenza virus subtypes in time and space between poultry and wild birds; Towards improvement of surveillance programs
Source: PLoS One. 2017 Mar 9;12(3):e0173470. doi: 10.1371/journal.pone.0173470 (PMC5344487; doi:10.1371/journal.pone.0173470)
Supplement: S1 Fig — Black indicates poultry farms or wild birds that tested positive for avian influenza viruses, grey indicates poultry farms or wild birds that tested negative for avian influenza viruses. The figure is derived from TOP10NL, Kadaster, Basisregistratie Topografie (BRT), licensed under CC-BY-4.0. (PDF) [file pone.0173470.s001.pdf]

Supporting Information

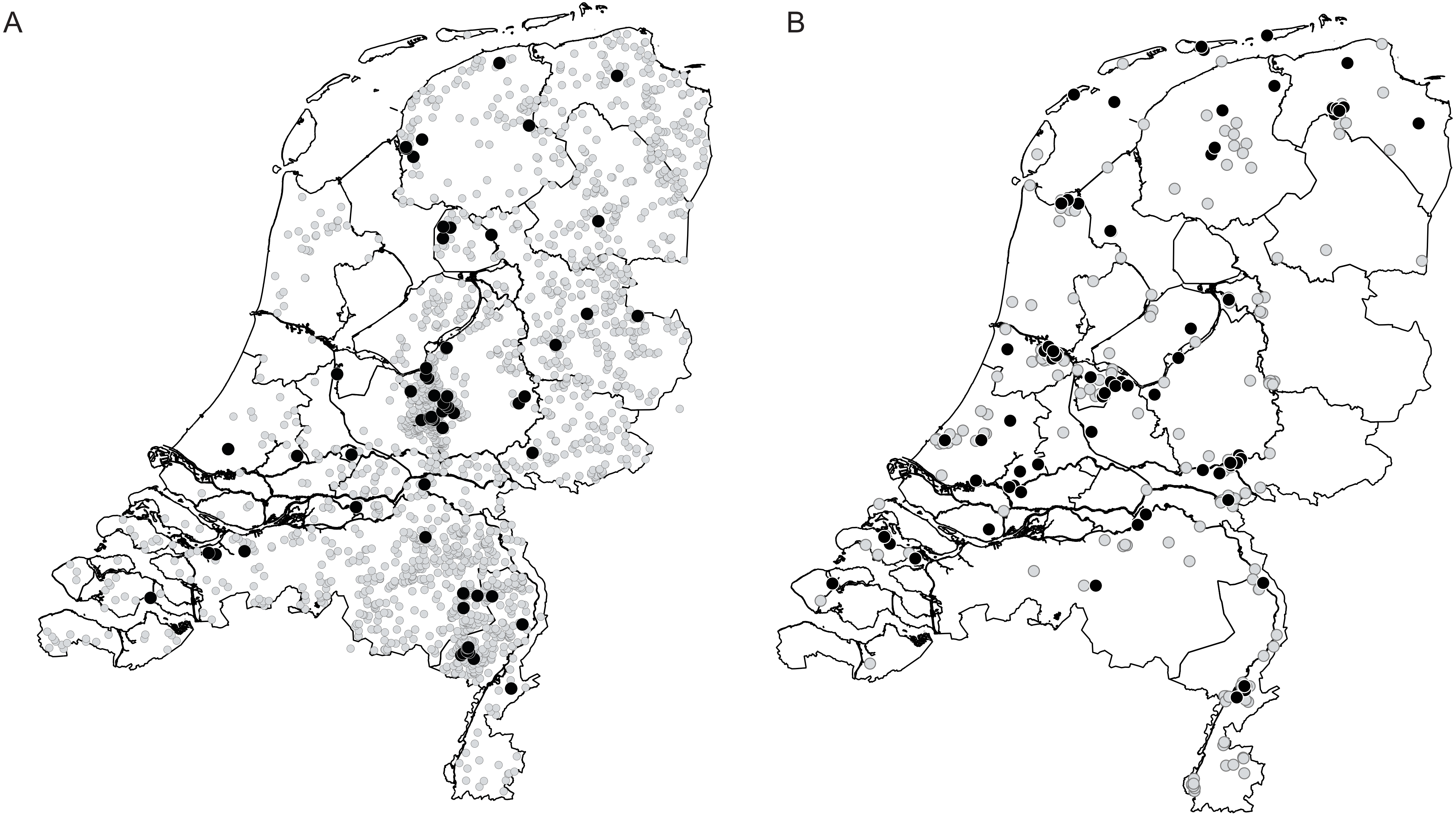

S1 Figure. Distribution of poultry farms (A) and sites of wild bird sampling (B) within the Netherlands, 2006 to 2011. Black indicates poultry farms or wild birds that tested positive for avian influenza viruses, grey indicates poultry farms or wild birds that tested negative for avian influenza viruses. The figure is derived from TOP10NL, Kadaster, Basisregistratie Topografie (BRT), licenced under CC-BY-4.0.
