# Supplementary material for: Discordant detection of avian influenza virus subtypes in time and space between poultry and wild birds; Towards improvement of surveillance programs
Source: PLoS One. 2017 Mar 9;12(3):e0173470. doi: 10.1371/journal.pone.0173470 (PMC5344487; doi:10.1371/journal.pone.0173470)
Supplement: S2 Fig — H1 (A), H6 (B), H7 (C), H8 (D), H9 (E), H10 (F), N1 (G), N2 (H), N4 (I), N5 (J) and N7 (K). Red indicates influenza viruses isolated from poultry in the Netherlands within this study period and blue indicates the genetically closest influenza virus isolated from wild birds. (PDF) [file pone.0173470.s002.pdf]

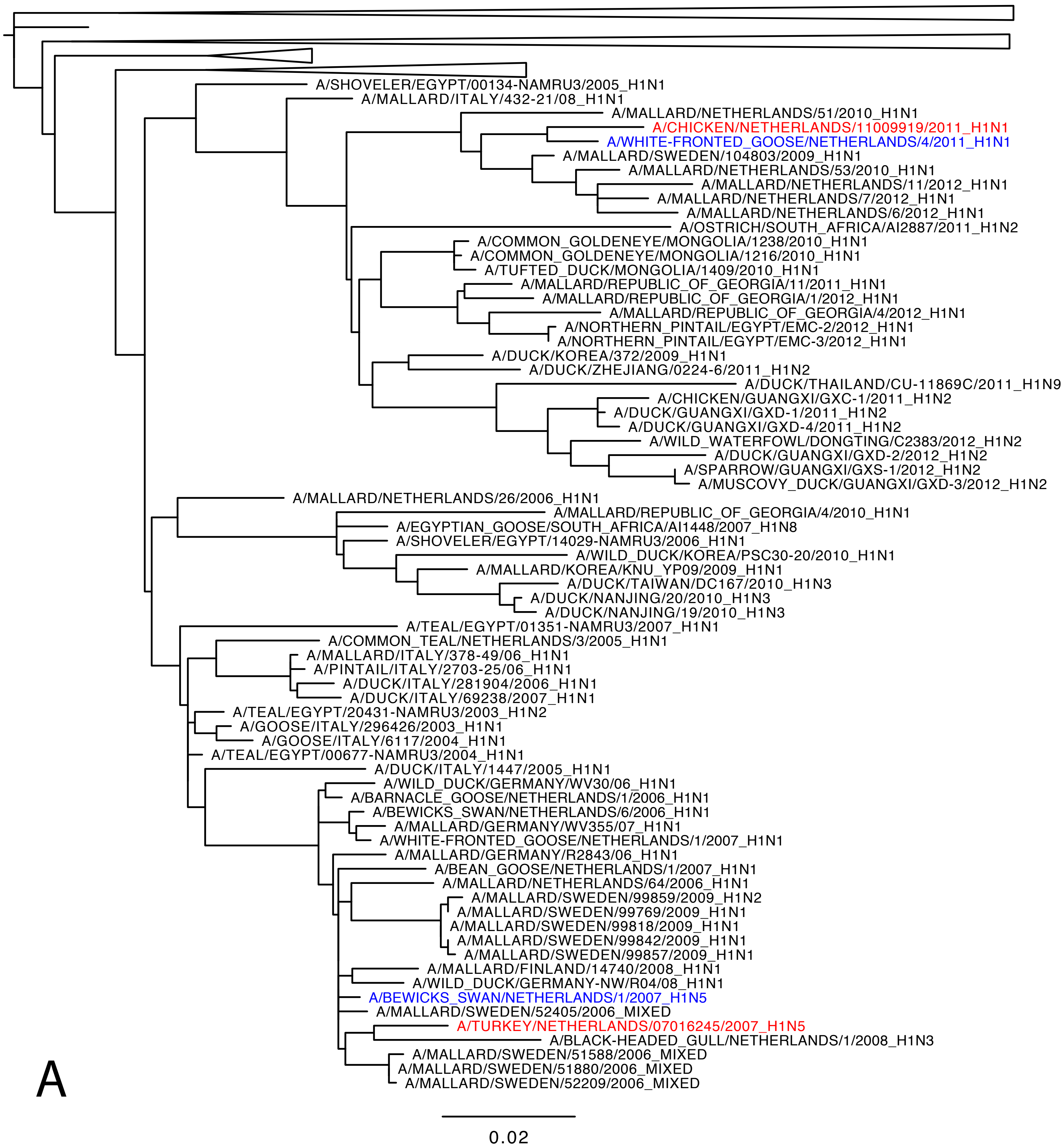
A/SHOVELER/EGYPT/00134-NAMRU3/2005_H1N1
A/MALLARD/ITALY/432-21/08_H1N1
A/MALLARD/NETHERLANDS/51/2010_H1N1
A/CHICKEN/NETHERLANDS/11009919/2011_H1N1
A/WHITE-FRONTED_GOOSE/NETHERLANDS/4/2011_H1N1
A/MALLARD/SWEDEN/104803/2009_H1N1
A/MALLARD/NETHERLANDS/53/2010_H1N1
A/MALLARD/NETHERLANDS/11/2012_H1N1
A/MALLARD/NETHERLANDS/7/2012_H1N1
A/MALLARD/NETHERLANDS/6/2012_H1N1
A/OSTRICH/SOUTH_AFRICA/AI2887/2011_H1N2
A/COMMON_GOLDENEYE/MONGOLIA/1238/2010_H1N1
A/COMMON_GOLDENEYE/MONGOLIA/1216/2010_H1N1
A/TUFTED_DUCK/MONGOLIA/1409/2010_H1N1
A/MALLARD/REPUBLIC_OF_GEORGIA/11/2011_H1N1
A/MALLARD/REPUBLIC_OF_GEORGIA/1/2012_H1N1
A/MALLARD/REPUBLIC_OF_GEORGIA/4/2012_H1N1
A/NORTHERN_PINTAIL/EGYPT/EMC-2/2012_H1N1
A/NORTHERN_PINTAIL/EGYPT/EMC-3/2012_H1N1
A/DUCK/KOREA/372/2009_H1N1
A/DUCK/ZHEJIANG/0224-6/2011_H1N2
A/DUCK/THAILAND/CU-11869C/2011_H1N9
A/CHICKEN/GUANGXI/GXC-1/2011_H1N2
A/DUCK/GUANGXI/GXD-1/2011_H1N2
A/DUCK/GUANGXI/GXD-4/2011_H1N2
A/WILD_WATERFOWL/DONGTING/C2383/2012_H1N2
A/DUCK/GUANGXI/GXD-2/2012_H1N2
A/SPARROW/GUANGXI/GXS-1/2012_H1N2
A/MUSCOVY_DUCK/GUANGXI/GXD-3/2012_H1N2
A/MALLARD/NETHERLANDS/26/2006_H1N1
A/MALLARD/REPUBLIC_OF_GEORGIA/4/2010_H1N1
A/EGYPTIAN_GOOSE/SOUTH_AFRICA/AI1448/2007_H1N8
A/SHOVELER/EGYPT/14029-NAMRU3/2006_H1N1
A/WILD_DUCK/KOREA/PSC30-20/2010_H1N1
A/MALLARD/KOREA/KNU_YP09/2009_H1N1
A/DUCK/TAIWAN/DC167/2010_H1N3
A/DUCK/NANJING/20/2010_H1N3
A/DUCK/NANJING/19/2010_H1N3
A/TEAL/EGYPT/01351-NAMRU3/2007_H1N1
A/COMMON_TEAL/NETHERLANDS/3/2005_H1N1
A/MALLARD/ITALY/378-49/06_H1N1
A/PINTAIL/ITALY/2703-25/06_H1N1
A/DUCK/ITALY/281904/2006_H1N1
A/DUCK/ITALY/69238/2007_H1N1
A/TEAL/EGYPT/20431-NAMRU3/2003_H1N2
A/GOOSE/ITALY/296426/2003_H1N1
A/GOOSE/ITALY/6117/2004_H1N1
A/TEAL/EGYPT/00677-NAMRU3/2004_H1N1
A/DUCK/ITALY/1447/2005_H1N1
A/WILD_DUCK/GERMANY/WV30/06_H1N1
A/BARNACLE_GOOSE/NETHERLANDS/1/2006_H1N1
A/BEWICKS_SWAN/NETHERLANDS/6/2006_H1N1
A/MALLARD/GERMANY/WV355/07_H1N1
A/WHITE-FRONTED_GOOSE/NETHERLANDS/1/2007_H1N1
A/MALLARD/GERMANY/R2843/06_H1N1
A/BEAN_GOOSE/NETHERLANDS/1/2007_H1N1
A/MALLARD/NETHERLANDS/64/2006_H1N1
A/MALLARD/SWEDEN/99859/2009_H1N2
A/MALLARD/SWEDEN/99769/2009_H1N1
A/MALLARD/SWEDEN/99818/2009_H1N1
A/MALLARD/SWEDEN/99842/2009_H1N1
A/MALLARD/SWEDEN/99857/2009_H1N1
A/MALLARD/FINLAND/14740/2008_H1N1
A/WILD_DUCK/GERMANY-NW/R04/08_H1N1
A/BEWICKS_SWAN/NETHERLANDS/1/2007_H1N5
A/MALLARD/SWEDEN/52405/2006_MIXED
A/TURKEY/NETHERLANDS/07016245/2007_H1N5
A/BLACK-HEADED_GULL/NETHERLANDS/1/2008_H1N3
A/MALLARD/SWEDEN/51588/2006_MIXED
A/MALLARD/SWEDEN/51880/2006_MIXED
A/MALLARD/SWEDEN/52209/2006_MIXED
A
0.02

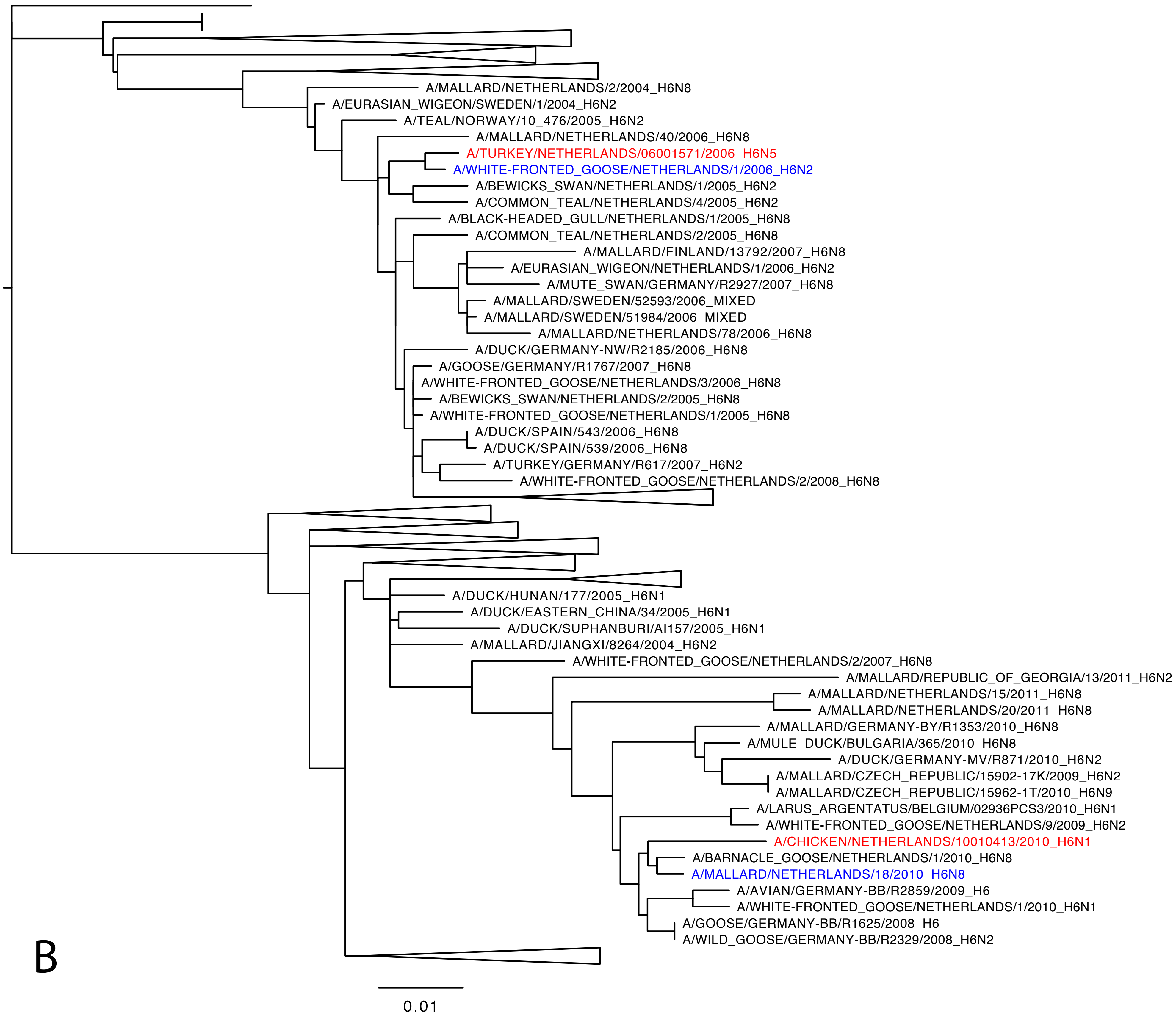
A/MALLARD/NETHERLANDS/2/2004_H6N8
A/EURASIAN_WIGEON/SWEDEN/1/2004_H6N2
A/TEAL/NORWAY/10_476/2005_H6N2
A/MALLARD/NETHERLANDS/40/2006_H6N8
A/TURKEY/NETHERLANDS/06001571/2006_H6N5
A/WHITE-FRONTED_GOOSE/NETHERLANDS/1/2006_H6N2
A/BEWICKS_SWAN/NETHERLANDS/1/2005_H6N2
A/COMMON_TEAL/NETHERLANDS/4/2005_H6N2
A/BLACK-HEADED_GULL/NETHERLANDS/1/2005_H6N8
A/COMMON_TEAL/NETHERLANDS/2/2005_H6N8
A/MALLARD/FINLAND/13792/2007_H6N8
A/EURASIAN_WIGEON/NETHERLANDS/1/2006_H6N2
A/MUTE_SWAN/GERMANY/R2927/2007_H6N8
A/MALLARD/SWEDEN/52593/2006_MIXED
A/MALLARD/SWEDEN/51984/2006_MIXED
A/MALLARD/NETHERLANDS/78/2006_H6N8
A/DUCK/GERMANY-NW/R2185/2006_H6N8
A/GOOSE/GERMANY/R1767/2007_H6N8
A/WHITE-FRONTED_GOOSE/NETHERLANDS/3/2006_H6N8
A/BEWICKS_SWAN/NETHERLANDS/2/2005_H6N8
A/WHITE-FRONTED_GOOSE/NETHERLANDS/1/2005_H6N8
A/DUCK/SPAIN/543/2006_H6N8
A/DUCK/SPAIN/539/2006_H6N8
A/TURKEY/GERMANY/R617/2007_H6N2
A/WHITE-FRONTED_GOOSE/NETHERLANDS/2/2008_H6N8
A/DUCK/HUNAN/177/2005_H6N1
A/DUCK/EASTERN_CHINA/34/2005_H6N1
A/DUCK/SUPHANBURI/AI157/2005_H6N1
A/MALLARD/JIANGXI/8264/2004_H6N2
A/WHITE-FRONTED_GOOSE/NETHERLANDS/2/2007_H6N8
A/MALLARD/REPUBLIC_OF_GEORGIA/13/2011_H6N2
A/MALLARD/NETHERLANDS/15/2011_H6N8
A/MALLARD/NETHERLANDS/20/2011_H6N8
A/MALLARD/GERMANY-BY/R1353/2010_H6N8
A/MULE_DUCK/BULGARIA/365/2010_H6N8
A/DUCK/GERMANY-MV/R871/2010_H6N2
A/MALLARD/CZECH_REPUBLIC/15902-17K/2009_H6N2
A/MALLARD/CZECH_REPUBLIC/15962-1T/2010_H6N9
A/LARUS_ARGENTATUS/BELGIUM/02936PCS3/2010_H6N1
A/WHITE-FRONTED_GOOSE/NETHERLANDS/9/2009_H6N2
A/CHICKEN/NETHERLANDS/10010413/2010_H6N1
A/BARNACLE_GOOSE/NETHERLANDS/1/2010_H6N8
A/MALLARD/NETHERLANDS/18/2010_H6N8
A/AVIAN/GERMANY-BB/R2859/2009_H6
A/WHITE-FRONTED_GOOSE/NETHERLANDS/1/2010_H6N1
A/GOOSE/GERMANY-BB/R1625/2008_H6
A/WILD_GOOSE/GERMANY-BB/R2329/2008_H6N2
B
0.01

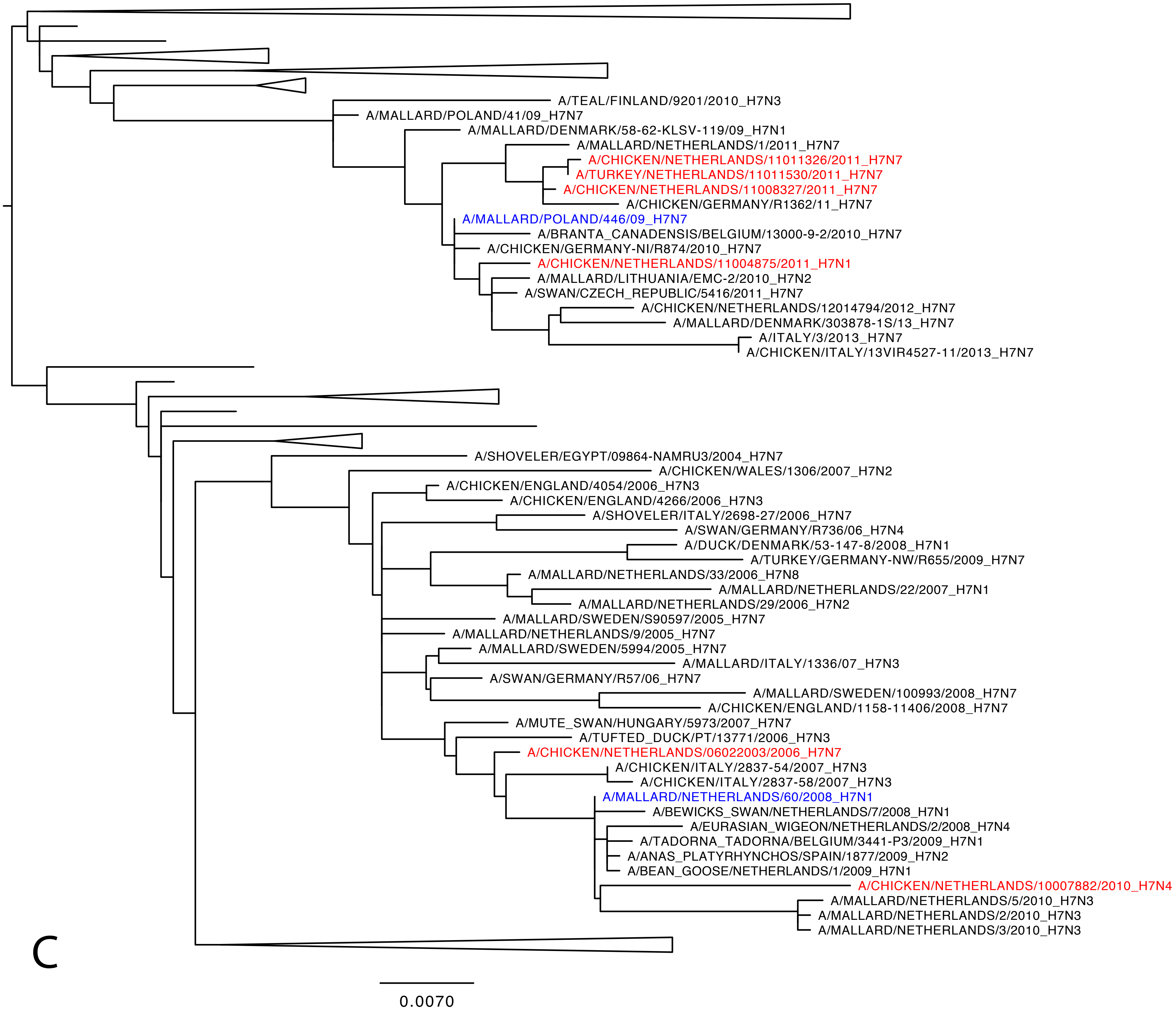
A/TEAL/FINLAND/9201/2010_H7N3
A/MALLARD/POLAND/41/09_H7N7
A/MALLARD/DENMARK/58-62-KLSV-119/09_H7N1
A/MALLARD/NETHERLANDS/1/2011_H7N7
A/CHICKEN/NETHERLANDS/11011326/2011_H7N7
A/TURKEY/NETHERLANDS/11011530/2011_H7N7
A/CHICKEN/NETHERLANDS/11008327/2011_H7N7
A/CHICKEN/GERMANY/R1362/11_H7N7
A/MALLARD/POLAND/446/09_H7N7
A/BRANTA_CANADENSIS/BELGIUM/13000-9-2/2010_H7N7
A/CHICKEN/GERMANY-NI/R874/2010_H7N7
A/CHICKEN/NETHERLANDS/11004875/2011_H7N1
A/MALLARD/LITHUANIA/EMC-2/2010_H7N2
A/SWAN/CZECH_REPUBLIC/5416/2011_H7N7
A/CHICKEN/NETHERLANDS/12014794/2012_H7N7
A/MALLARD/DENMARK/303878-1S/13_H7N7
A/ITALY/3/2013_H7N7
A/CHICKEN/ITALY/13VIR4527-11/2013_H7N7
A/SHOVELER/EGYPT/09864-NAMRU3/2004_H7N7
A/CHICKEN/WALES/1306/2007_H7N2
A/CHICKEN/ENGLAND/4054/2006_H7N3
A/CHICKEN/ENGLAND/4266/2006_H7N3
A/SHOVELER/ITALY/2698-27/2006_H7N7
A/SWAN/GERMANY/R736/06_H7N4
A/DUCK/DENMARK/53-147-8/2008_H7N1
A/TURKEY/GERMANY-NW/R655/2009_H7N7
A/MALLARD/NETHERLANDS/33/2006_H7N8
A/MALLARD/NETHERLANDS/22/2007_H7N1
A/MALLARD/NETHERLANDS/29/2006_H7N2
A/MALLARD/SWEDEN/S90597/2005_H7N7
A/MALLARD/NETHERLANDS/9/2005_H7N7
A/MALLARD/SWEDEN/5994/2005_H7N7
A/MALLARD/ITALY/1336/07_H7N3
A/SWAN/GERMANY/R57/06_H7N7
A/MALLARD/SWEDEN/100993/2008_H7N7
A/CHICKEN/ENGLAND/1158-11406/2008_H7N7
A/MUTE_SWAN/HUNGARY/5973/2007_H7N7
A/TUFTED_DUCK/PT/13771/2006_H7N3
A/CHICKEN/NETHERLANDS/06022003/2006_H7N7
A/CHICKEN/ITALY/2837-54/2007_H7N3
A/CHICKEN/ITALY/2837-58/2007_H7N3
A/MALLARD/NETHERLANDS/60/2008_H7N1
A/BEWICKS_SWAN/NETHERLANDS/7/2008_H7N1
A/EURASIAN_WIGEON/NETHERLANDS/2/2008_H7N4
A/TADORNA_TADORNA/BELGIUM/3441-P3/2009_H7N1
A/ANAS_PLATYRHYNCHOS/SPAIN/1877/2009_H7N2
A/BEAN_GOOSE/NETHERLANDS/1/2009_H7N1
A/CHICKEN/NETHERLANDS/10007882/2010_H7N4
A/MALLARD/NETHERLANDS/5/2010_H7N3
A/MALLARD/NETHERLANDS/2/2010_H7N3
A/MALLARD/NETHERLANDS/3/2010_H7N3
C
0.0070

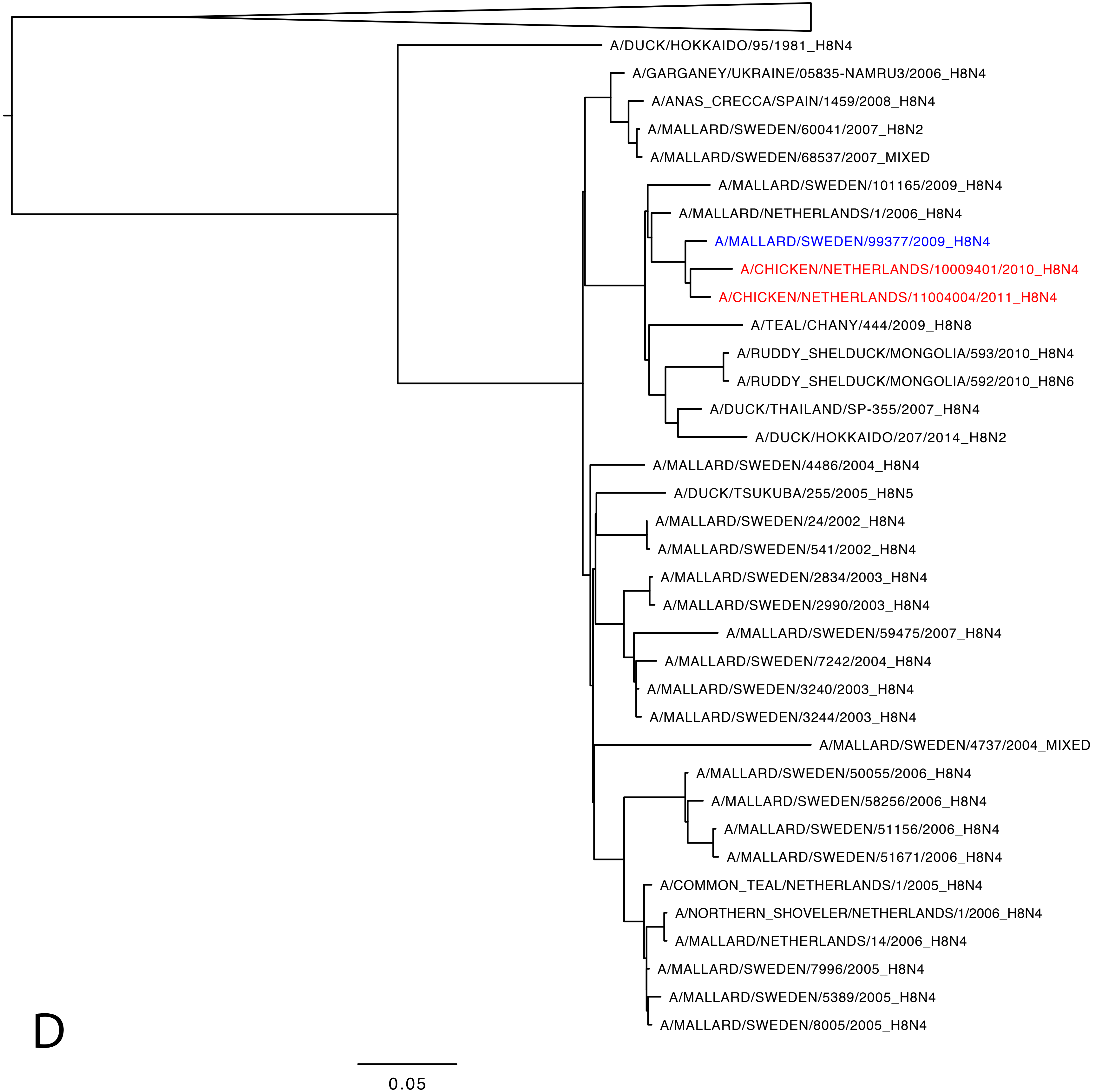
A/DUCK/HOKKAIDO/95/1981_H8N4
A/GARGANEY/UKRAINE/05835-NAMRU3/2006_H8N4
A/ANAS_CRECCA/SPAIN/1459/2008_H8N4
A/MALLARD/SWEDEN/60041/2007_H8N2
A/MALLARD/SWEDEN/68537/2007_MIXED
A/MALLARD/SWEDEN/101165/2009_H8N4
A/MALLARD/NETHERLANDS/1/2006_H8N4
A/MALLARD/SWEDEN/99377/2009_H8N4
A/CHICKEN/NETHERLANDS/10009401/2010_H8N4
A/CHICKEN/NETHERLANDS/11004004/2011_H8N4
A/TEAL/CHANY/444/2009_H8N8
A/RUDDY_SHELDUCK/MONGOLIA/593/2010_H8N4
A/RUDDY_SHELDUCK/MONGOLIA/592/2010_H8N6
A/DUCK/THAILAND/SP-355/2007_H8N4
A/DUCK/HOKKAIDO/207/2014_H8N2
A/MALLARD/SWEDEN/4486/2004_H8N4
A/DUCK/TSUKUBA/255/2005_H8N5
A/MALLARD/SWEDEN/24/2002_H8N4
A/MALLARD/SWEDEN/541/2002_H8N4
A/MALLARD/SWEDEN/2834/2003_H8N4
A/MALLARD/SWEDEN/2990/2003_H8N4
A/MALLARD/SWEDEN/59475/2007_H8N4
A/MALLARD/SWEDEN/7242/2004_H8N4
A/MALLARD/SWEDEN/3240/2003_H8N4
A/MALLARD/SWEDEN/3244/2003_H8N4
A/MALLARD/SWEDEN/4737/2004_MIXED
A/MALLARD/SWEDEN/50055/2006_H8N4
A/MALLARD/SWEDEN/58256/2006_H8N4
A/MALLARD/SWEDEN/51156/2006_H8N4
A/MALLARD/SWEDEN/51671/2006_H8N4
A/COMMON_TEAL/NETHERLANDS/1/2005_H8N4
A/NORTHERN_SHOVELER/NETHERLANDS/1/2006_H8N4
A/MALLARD/NETHERLANDS/14/2006_H8N4
A/MALLARD/SWEDEN/7996/2005_H8N4
A/MALLARD/SWEDEN/5389/2005_H8N4
A/MALLARD/SWEDEN/8005/2005_H8N4
D
0.05

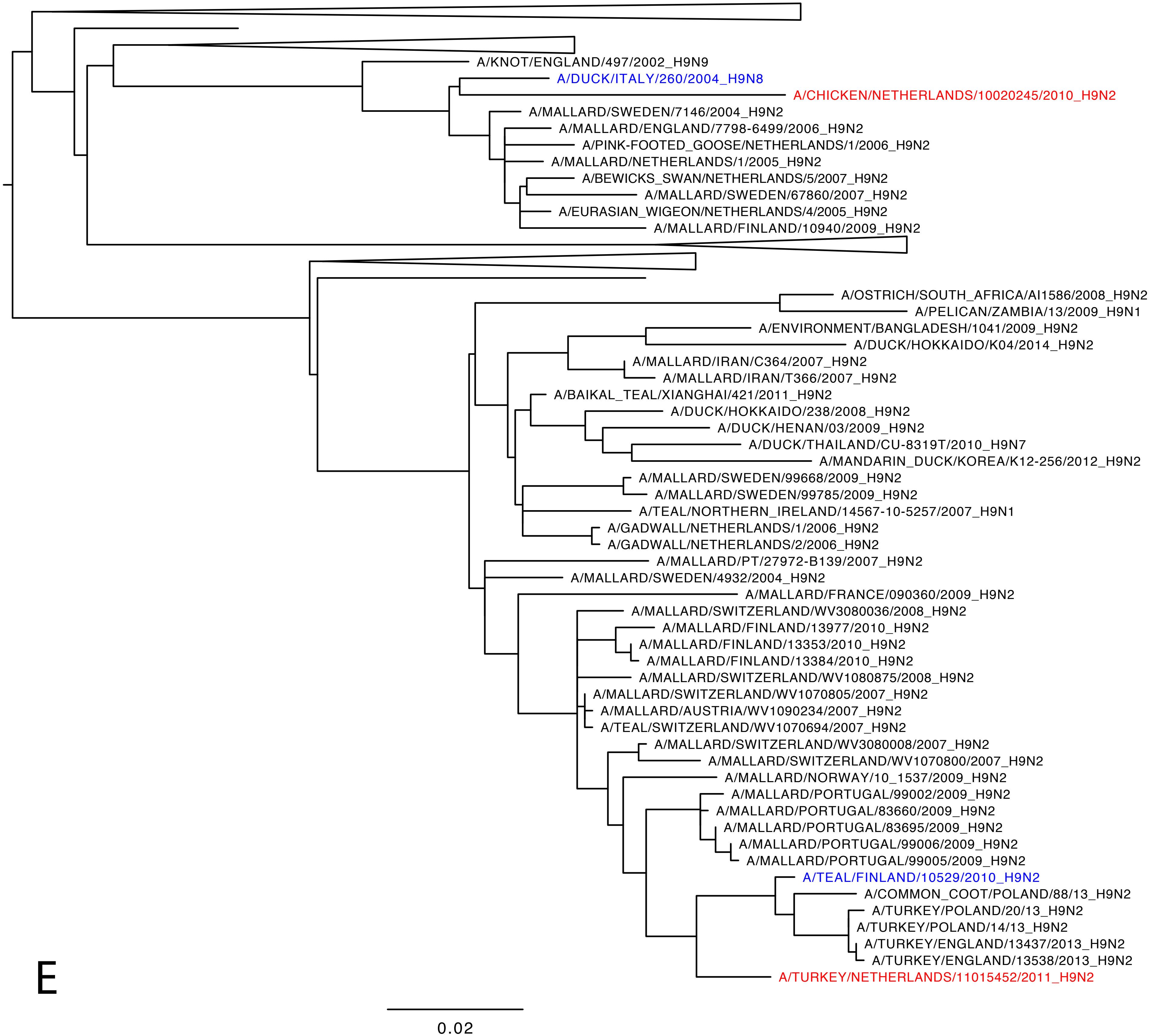
A/KNOT/ENGLAND/497/2002_H9N9
A/DUCK/ITALY/260/2004_H9N8
A/CHICKEN/NETHERLANDS/10020245/2010_H9N2
A/MALLARD/SWEDEN/7146/2004_H9N2
A/MALLARD/ENGLAND/7798-6499/2006_H9N2
A/PINK-FOOTED_GOOSE/NETHERLANDS/1/2006_H9N2
A/MALLARD/NETHERLANDS/1/2005_H9N2
A/BEWICKS_SWAN/NETHERLANDS/5/2007_H9N2
A/MALLARD/SWEDEN/67860/2007_H9N2
A/EURASIAN_WIGEON/NETHERLANDS/4/2005_H9N2
A/MALLARD/FINLAND/10940/2009_H9N2
A/OSTRICH/SOUTH_AFRICA/AI1586/2008_H9N2
A/PELICAN/ZAMBIA/13/2009_H9N1
A/ENVIRONMENT/BANGLADESH/1041/2009_H9N2
A/DUCK/HOKKAIDO/K04/2014_H9N2
A/MALLARD/IRAN/C364/2007_H9N2
A/MALLARD/IRAN/T366/2007_H9N2
A/BAIKAL_TEAL/XIANGHAI/421/2011_H9N2
A/DUCK/HOKKAIDO/238/2008_H9N2
A/DUCK/HENAN/03/2009_H9N2
A/DUCK/THAILAND/CU-8319T/2010_H9N7
A/MANDARIN_DUCK/KOREA/K12-256/2012_H9N2
A/MALLARD/SWEDEN/99668/2009_H9N2
A/MALLARD/SWEDEN/99785/2009_H9N2
A/TEAL/NORTHERN_IRELAND/14567-10-5257/2007_H9N1
A/GADWALL/NETHERLANDS/1/2006_H9N2
A/GADWALL/NETHERLANDS/2/2006_H9N2
A/MALLARD/PT/27972-B139/2007_H9N2
A/MALLARD/SWEDEN/4932/2004_H9N2
A/MALLARD/FRANCE/090360/2009_H9N2
A/MALLARD/SWITZERLAND/WV3080036/2008_H9N2
A/MALLARD/FINLAND/13977/2010_H9N2
A/MALLARD/FINLAND/13353/2010_H9N2
A/MALLARD/FINLAND/13384/2010_H9N2
A/MALLARD/SWITZERLAND/WV1080875/2008_H9N2
A/MALLARD/SWITZERLAND/WV1070805/2007_H9N2
A/MALLARD/AUSTRIA/WV1090234/2007_H9N2
A/TEAL/SWITZERLAND/WV1070694/2007_H9N2
A/MALLARD/SWITZERLAND/WV3080008/2007_H9N2
A/MALLARD/SWITZERLAND/WV1070800/2007_H9N2
A/MALLARD/NORWAY/10_1537/2009_H9N2
A/MALLARD/PORTUGAL/99002/2009_H9N2
A/MALLARD/PORTUGAL/83660/2009_H9N2
A/MALLARD/PORTUGAL/83695/2009_H9N2
A/MALLARD/PORTUGAL/99006/2009_H9N2
A/MALLARD/PORTUGAL/99005/2009_H9N2
A/TEAL/FINLAND/10529/2010_H9N2
A/COMMON_COOT/POLAND/88/13_H9N2
A/TURKEY/POLAND/20/13_H9N2
A/TURKEY/POLAND/14/13_H9N2
A/TURKEY/ENGLAND/13437/2013_H9N2
A/TURKEY/ENGLAND/13538/2013_H9N2
A/TURKEY/NETHERLANDS/11015452/2011_H9N2
E
0.02

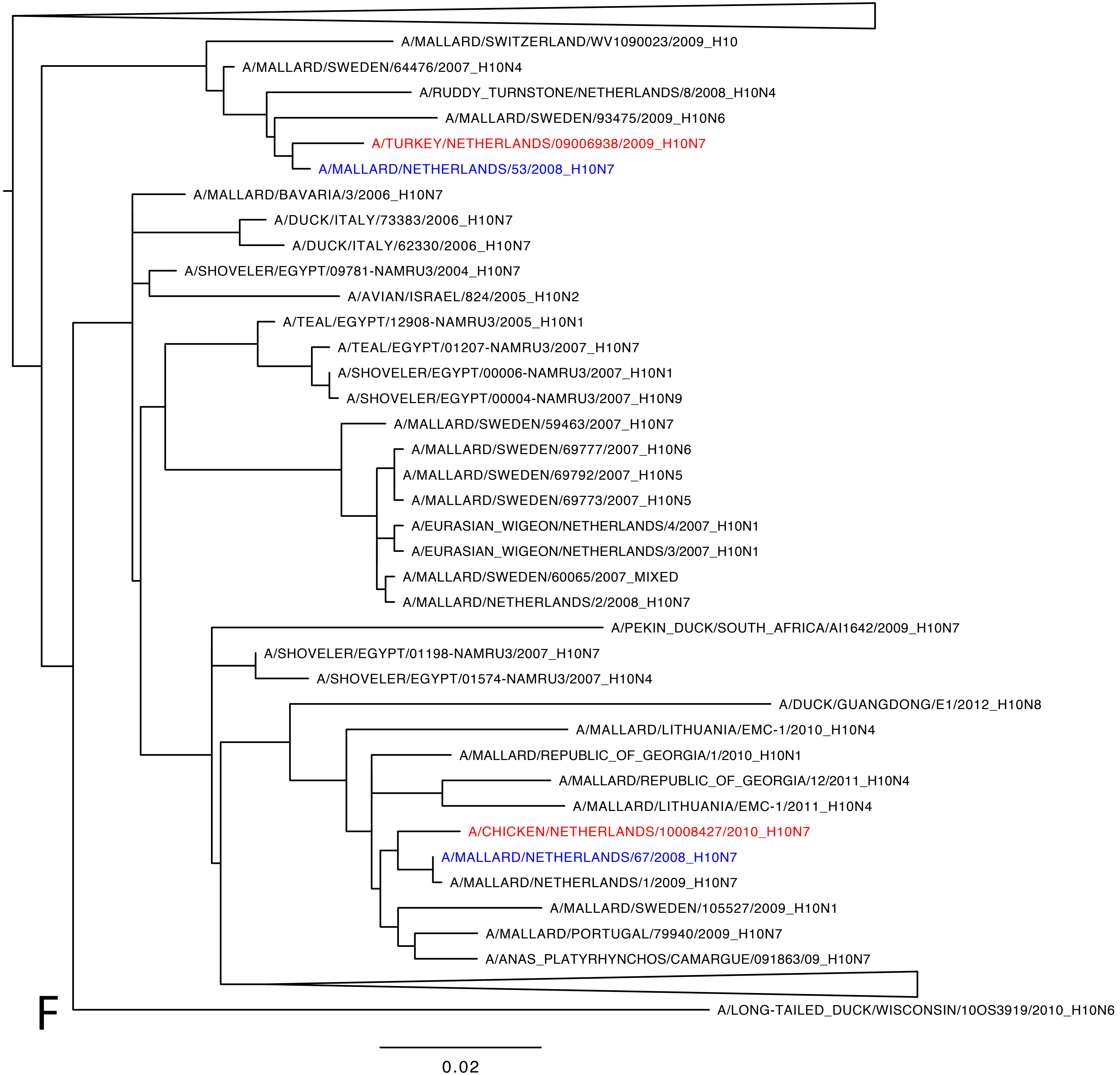
A/MALLARD/SWITZERLAND/WV1090023/2009_H10
A/MALLARD/SWEDEN/64476/2007_H10N4
A/RUDDY_TURNSTONE/NETHERLANDS/8/2008_H10N4
A/MALLARD/SWEDEN/93475/2009_H10N6
A/TURKEY/NETHERLANDS/09006938/2009_H10N7
A/MALLARD/NETHERLANDS/53/2008_H10N7
A/MALLARD/BAVARIA/3/2006_H10N7
A/DUCK/ITALY/73383/2006_H10N7
A/DUCK/ITALY/62330/2006_H10N7
A/SHOVELER/EGYPT/09781-NAMRU3/2004_H10N7
A/AVIAN/ISRAEL/824/2005_H10N2
A/TEAL/EGYPT/12908-NAMRU3/2005_H10N1
A/TEAL/EGYPT/01207-NAMRU3/2007_H10N7
A/SHOVELER/EGYPT/00006-NAMRU3/2007_H10N1
A/SHOVELER/EGYPT/00004-NAMRU3/2007_H10N9
A/MALLARD/SWEDEN/59463/2007_H10N7
A/MALLARD/SWEDEN/69777/2007_H10N6
A/MALLARD/SWEDEN/69792/2007_H10N5
A/MALLARD/SWEDEN/69773/2007_H10N5
A/EURASIAN_WIGEON/NETHERLANDS/4/2007_H10N1
A/EURASIAN_WIGEON/NETHERLANDS/3/2007_H10N1
A/MALLARD/SWEDEN/60065/2007_MIXED
A/MALLARD/NETHERLANDS/2/2008_H10N7
A/PEKIN_DUCK/SOUTH_AFRICA/AI1642/2009_H10N7
A/SHOVELER/EGYPT/01198-NAMRU3/2007_H10N7
A/SHOVELER/EGYPT/01574-NAMRU3/2007_H10N4
A/DUCK/GUANGDONG/E1/2012_H10N8
A/MALLARD/LITHUANIA/EMC-1/2010_H10N4
A/MALLARD/REPUBLIC_OF_GEORGIA/1/2010_H10N1
A/MALLARD/REPUBLIC_OF_GEORGIA/12/2011_H10N4
A/MALLARD/LITHUANIA/EMC-1/2011_H10N4
A/CHICKEN/NETHERLANDS/10008427/2010_H10N7
A/MALLARD/NETHERLANDS/67/2008_H10N7
A/MALLARD/NETHERLANDS/1/2009_H10N7
A/MALLARD/SWEDEN/105527/2009_H10N1
A/MALLARD/PORTUGAL/79940/2009_H10N7
A/ANAS_PLATYRHYNCHOS/CAMARGUE/091863/09_H10N7
A/LONG-TAILED_DUCK/WISCONSIN/10OS3919/2010_H10N6
F
0.02

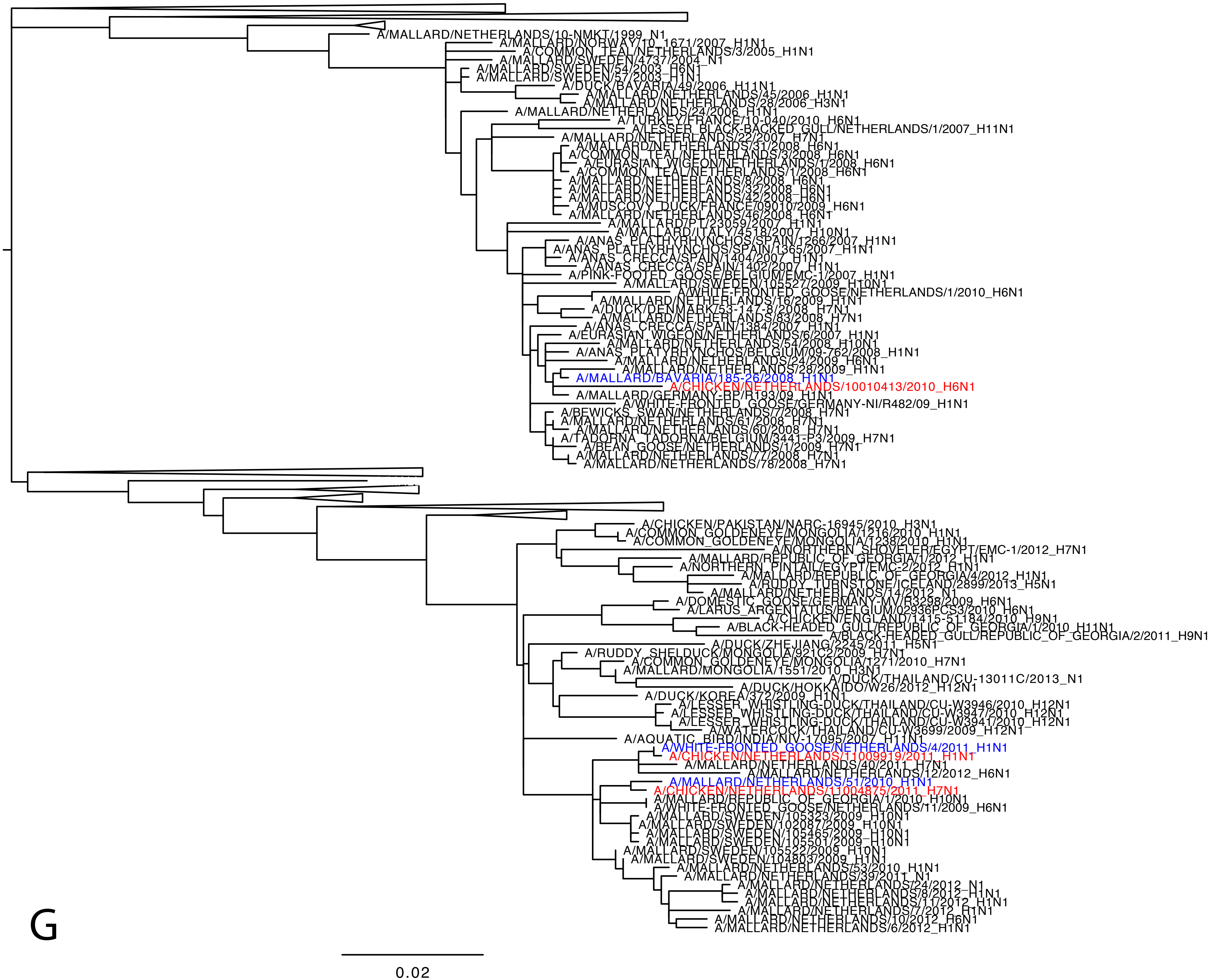
A/MALLARD/NETHERLANDS/10-NMKT/1999_N1
A/MALLARD/NORWAY/10_1671/2007_H1N1
A/COMMON_TEAL/NETHERLANDS/3/2005_H1N1
A/MALLARD/SWEDEN/4737/2004_N1
A/MALLARD/SWEDEN/54/2003_H6N1
A/MALLARD/SWEDEN/57/2003_H1N1
A/DUCK/BAVARIA/49/2006_H11N1
A/MALLARD/NETHERLANDS/45/2006_H1N1
A/MALLARD/NETHERLANDS/28/2006_H3N1
A/MALLARD/NETHERLANDS/24/2006_H1N1
A/TURKEY/FRANCE/10-040/2010_H6N1
A/LESSER_BLACK-BACKED_GULL/NETHERLANDS/1/2007_H11N1
A/MALLARD/NETHERLANDS/22/2007_H7N1
A/MALLARD/NETHERLANDS/31/2008_H6N1
A/COMMON_TEAL/NETHERLANDS/3/2008_H6N1
A/EURASIAN_WIGEON/NETHERLANDS/1/2008_H6N1
A/COMMON_TEAL/NETHERLANDS/1/2008_H6N1
A/MALLARD/NETHERLANDS/8/2008_H6N1
A/MALLARD/NETHERLANDS/32/2008_H6N1
A/MALLARD/NETHERLANDS/42/2008_H6N1
A/MUSCOVY_DUCK/FRANCE/09010/2009_H6N1
A/MALLARD/NETHERLANDS/46/2008_H6N1
A/MALLARD/PT/23059/2007_H1N1
A/MALLARD/ITALY/4518/2007_H10N1
A/ANAS_PLATHYRHYNCHOS/SPAIN/1266/2007_H1N1
A/ANAS_PLATHYRHYNCHOS/SPAIN/1365/2007_H1N1
A/ANAS_CRECCA/SPAIN/1404/2007_H1N1
A/ANAS_CRECCA/SPAIN/1402/2007_H1N1
A/PINK-FOOTED_GOOSE/BELGIUM/EMC-1/2007_H1N1
A/MALLARD/SWEDEN/105527/2009_H10N1
A/WHITE-FRONTED_GOOSE/NETHERLANDS/1/2010_H6N1
A/MALLARD/NETHERLANDS/16/2009_H1N1
A/DUCK/DENMARK/53-147-8/2008_H7N1
A/MALLARD/NETHERLANDS/83/2008_H7N1
A/ANAS_CRECCA/SPAIN/1384/2007_H1N1
A/EURASIAN_WIGEON/NETHERLANDS/6/2007_H1N1
A/MALLARD/NETHERLANDS/54/2008_H10N1
A/ANAS_PLATYRHYNCHOS/BELGIUM/09-762/2008_H1N1
A/MALLARD/NETHERLANDS/24/2009_H6N1
A/MALLARD/NETHERLANDS/28/2009_H1N1
A/MALLARD/BAVARIA/185-26/2008_H1N1
A/CHICKEN/NETHERLANDS/10010413/2010_H6N1
A/MALLARD/GERMANY-BP/R193/09_H1N1
A/WHITE-FRONTED_GOOSE/GERMANY-NI/R482/09_H1N1
A/BEWICKS_SWAN/NETHERLANDS/7/2008_H7N1
A/MALLARD/NETHERLANDS/61/2008_H7N1
A/MALLARD/NETHERLANDS/60/2008_H7N1
A/TADORNA_TADORNA/BELGIUM/3441-P3/2009_H7N1
A/BEAN_GOOSE/NETHERLANDS/1/2009_H7N1
A/MALLARD/NETHERLANDS/77/2008_H7N1
A/MALLARD/NETHERLANDS/78/2008_H7N1
A/CHICKEN/PAKISTAN/NARC-16945/2010_H3N1
A/COMMON_GOLDENEYE/MONGOLIA/1216/2010_H1N1
A/COMMON_GOLDENEYE/MONGOLIA/1238/2010_H1N1
A/NORTHERN_SHOVELER/EGYPT/EMC-1/2012_H7N1
A/MALLARD/REPUBLIC_OF_GEORGIA/1/2012_H1N1
A/NORTHERN_PINTAIL/EGYPT/EMC-2/2012_H1N1
A/MALLARD/REPUBLIC_OF_GEORGIA/4/2012_H1N1
A/RUDDY_TURNSTONE/ICELAND/2899/2013_H5N1
A/MALLARD/NETHERLANDS/14/2012_N1
A/DOMESTIC_GOOSE/GERMANY-MV/R3298/2009_H6N1
A/LARUS_ARGENTATUS/BELGIUM/02936PCS3/2010_H6N1
A/CHICKEN/ENGLAND/1415-51184/2010_H9N1
A/BLACK-HEADED_GULL/REPUBLIC_OF_GEORGIA/1/2010_H11N1
A/BLACK-HEADED_GULL/REPUBLIC_OF_GEORGIA/2/2011_H9N1
A/DUCK/ZHEJIANG/2245/2011_H5N1
A/RUDDY_SHELDUCK/MONGOLIA/921C2/2009_H7N1
A/COMMON_GOLDENEYE/MONGOLIA/1271/2010_H7N1
A/MALLARD/MONGOLIA/1551/2010_H3N1
A/DUCK/THAILAND/CU-13011C/2013_N1
A/DUCK/HOKKAIDO/W26/2012_H12N1
A/DUCK/KOREA/372/2009_H1N1
A/LESSER_WHISTLING-DUCK/THAILAND/CU-W3946/2010_H12N1
A/LESSER_WHISTLING-DUCK/THAILAND/CU-W3947/2010_H12N1
A/LESSER_WHISTLING-DUCK/THAILAND/CU-W3941/2010_H12N1
A/WATERCOCK/THAILAND/CU-W3699/2009_H12N1
A/AQUATIC_BIRD/INDIA/NIV-17095/2007_H11N1
A/WHITE-FRONTED_GOOSE/NETHERLANDS/4/2011_H1N1
A/CHICKEN/NETHERLANDS/11009919/2011_H1N1
A/MALLARD/NETHERLANDS/40/2011_H7N1
A/MALLARD/NETHERLANDS/12/2012_H6N1
A/MALLARD/NETHERLANDS/51/2010_H1N1
A/CHICKEN/NETHERLANDS/11004875/2011_H7N1
A/MALLARD/REPUBLIC_OF_GEORGIA/1/2010_H10N1
A/WHITE-FRONTED_GOOSE/NETHERLANDS/11/2009_H6N1
A/MALLARD/SWEDEN/105323/2009_H10N1
A/MALLARD/SWEDEN/102087/2009_H10N1
A/MALLARD/SWEDEN/105465/2009_H10N1
A/MALLARD/SWEDEN/105501/2009_H10N1
A/MALLARD/SWEDEN/105522/2009_H10N1
A/MALLARD/SWEDEN/104803/2009_H1N1
A/MALLARD/NETHERLANDS/53/2010_H1N1
A/MALLARD/NETHERLANDS/39/2011_N1
A/MALLARD/NETHERLANDS/24/2012_N1
A/MALLARD/NETHERLANDS/8/2012_H1N1
A/MALLARD/NETHERLANDS/11/2012_H1N1
A/MALLARD/NETHERLANDS/7/2012_H1N1
A/MALLARD/NETHERLANDS/10/2012_H6N1
A/MALLARD/NETHERLANDS/6/2012_H1N1
G
0.02

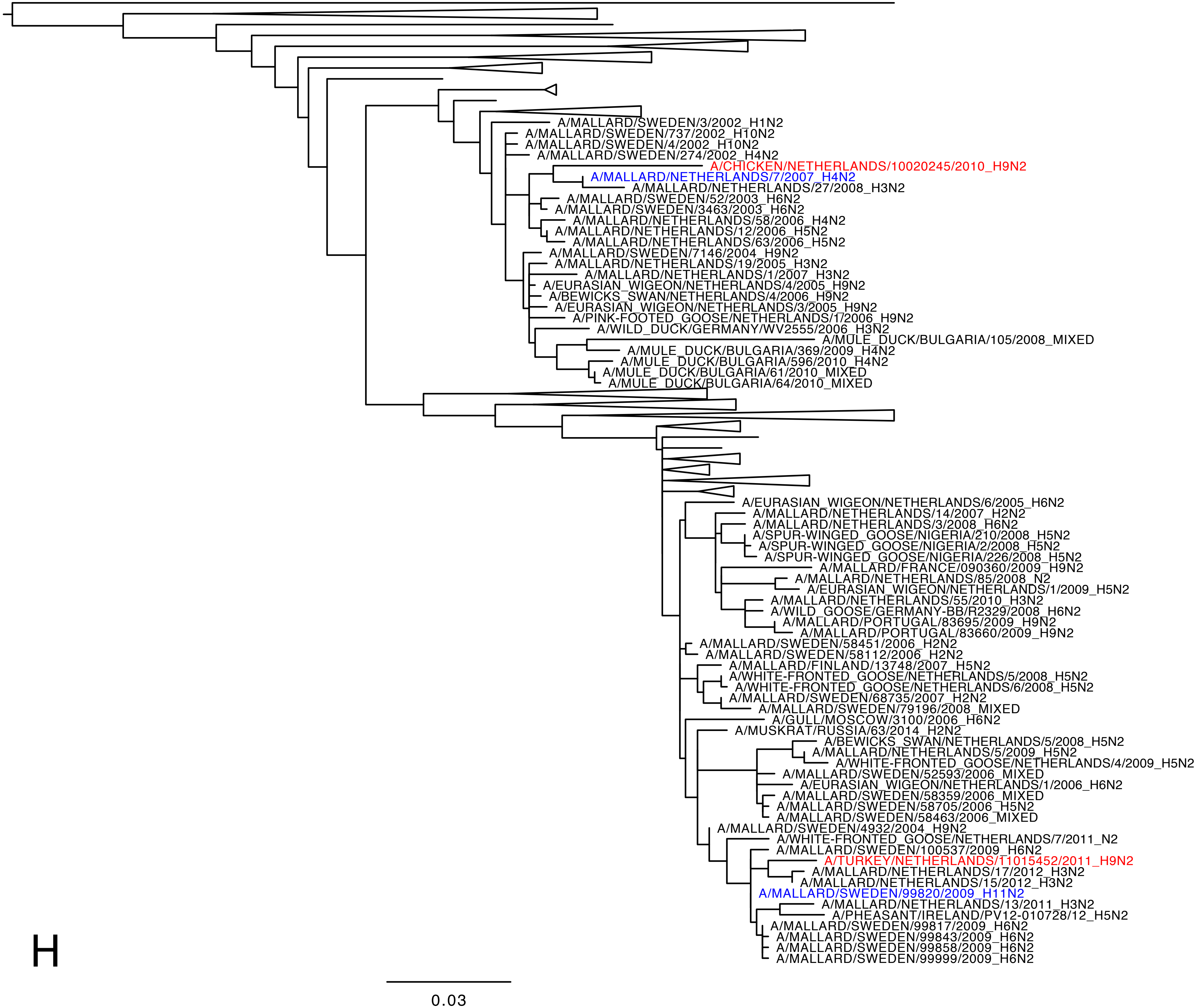
A/MALLARD/SWEDEN/3/2002_H1N2
A/MALLARD/SWEDEN/737/2002_H10N2
A/MALLARD/SWEDEN/4/2002_H10N2
A/MALLARD/SWEDEN/274/2002_H4N2
A/CHICKEN/NETHERLANDS/10020245/2010_H9N2
A/MALLARD/NETHERLANDS/7/2007_H4N2
A/MALLARD/NETHERLANDS/27/2008_H3N2
A/MALLARD/SWEDEN/52/2003_H6N2
A/MALLARD/SWEDEN/3463/2003_H6N2
A/MALLARD/NETHERLANDS/58/2006_H4N2
A/MALLARD/NETHERLANDS/12/2006_H5N2
A/MALLARD/NETHERLANDS/63/2006_H5N2
A/MALLARD/SWEDEN/7146/2004_H9N2
A/MALLARD/NETHERLANDS/19/2005_H3N2
A/MALLARD/NETHERLANDS/1/2007_H3N2
A/EURASIAN_WIGEON/NETHERLANDS/4/2005_H9N2
A/BEWICKS_SWAN/NETHERLANDS/4/2006_H9N2
A/EURASIAN_WIGEON/NETHERLANDS/3/2005_H9N2
A/PINK-FOOTED_GOOSE/NETHERLANDS/1/2006_H9N2
A/WILD_DUCK/GERMANY/WV2555/2006_H3N2
A/MULE_DUCK/BULGARIA/105/2008_MIXED
A/MULE_DUCK/BULGARIA/369/2009_H4N2
A/MULE_DUCK/BULGARIA/596/2010_H4N2
A/MULE_DUCK/BULGARIA/61/2010_MIXED
A/MULE_DUCK/BULGARIA/64/2010_MIXED
A/EURASIAN_WIGEON/NETHERLANDS/6/2005_H6N2
A/MALLARD/NETHERLANDS/14/2007_H2N2
A/MALLARD/NETHERLANDS/3/2008_H6N2
A/SPUR-WINGED_GOOSE/NIGERIA/210/2008_H5N2
A/SPUR-WINGED_GOOSE/NIGERIA/2/2008_H5N2
A/SPUR-WINGED_GOOSE/NIGERIA/226/2008_H5N2
A/MALLARD/FRANCE/090360/2009_H9N2
A/MALLARD/NETHERLANDS/85/2008_N2
A/EURASIAN_WIGEON/NETHERLANDS/1/2009_H5N2
A/MALLARD/NETHERLANDS/55/2010_H3N2
A/WILD_GOOSE/GERMANY-BB/R2329/2008_H6N2
A/MALLARD/PORTUGAL/83695/2009_H9N2
A/MALLARD/PORTUGAL/83660/2009_H9N2
A/MALLARD/SWEDEN/58451/2006_H2N2
A/MALLARD/SWEDEN/58112/2006_H2N2
A/MALLARD/FINLAND/13748/2007_H5N2
A/WHITE-FRONTED_GOOSE/NETHERLANDS/5/2008_H5N2
A/WHITE-FRONTED_GOOSE/NETHERLANDS/6/2008_H5N2
A/MALLARD/SWEDEN/68735/2007_H2N2
A/MALLARD/SWEDEN/79196/2008_MIXED
A/GULL/MOSCOW/3100/2006_H6N2
A/MUSKRAT/RUSSIA/63/2014_H2N2
A/BEWICKS_SWAN/NETHERLANDS/5/2008_H5N2
A/MALLARD/NETHERLANDS/5/2009_H5N2
A/WHITE-FRONTED_GOOSE/NETHERLANDS/4/2009_H5N2
A/MALLARD/SWEDEN/52593/2006_MIXED
A/EURASIAN_WIGEON/NETHERLANDS/1/2006_H6N2
A/MALLARD/SWEDEN/58359/2006_MIXED
A/MALLARD/SWEDEN/58705/2006_H5N2
A/MALLARD/SWEDEN/58463/2006_MIXED
A/MALLARD/SWEDEN/4932/2004_H9N2
A/WHITE-FRONTED_GOOSE/NETHERLANDS/7/2011_N2
A/MALLARD/SWEDEN/100537/2009_H6N2
A/TURKEY/NETHERLANDS/11015452/2011_H9N2
A/MALLARD/NETHERLANDS/17/2012_H3N2
A/MALLARD/NETHERLANDS/15/2012_H3N2
A/MALLARD/SWEDEN/99820/2009_H11N2
A/MALLARD/NETHERLANDS/13/2011_H3N2
A/PHEASANT/IRELAND/PV12-010728/12_H5N2
A/MALLARD/SWEDEN/99817/2009_H6N2
A/MALLARD/SWEDEN/99843/2009_H6N2
A/MALLARD/SWEDEN/99858/2009_H6N2
A/MALLARD/SWEDEN/99999/2009_H6N2
H
0.03

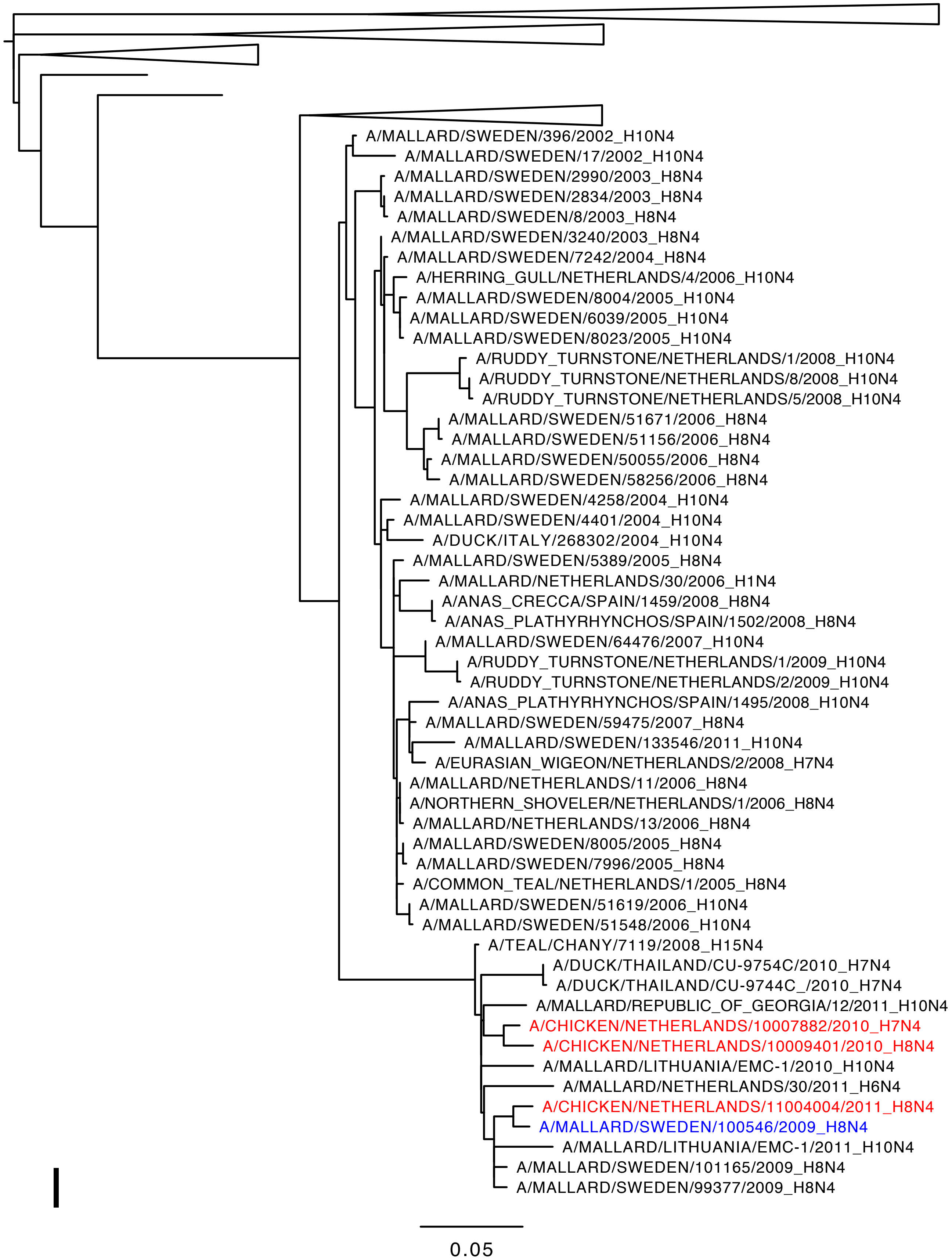
A/MALLARD/SWEDEN/396/2002_H10N4
A/MALLARD/SWEDEN/17/2002_H10N4
A/MALLARD/SWEDEN/2990/2003_H8N4
A/MALLARD/SWEDEN/2834/2003_H8N4
A/MALLARD/SWEDEN/8/2003_H8N4
A/MALLARD/SWEDEN/3240/2003_H8N4
A/MALLARD/SWEDEN/7242/2004_H8N4
A/HERRING_GULL/NETHERLANDS/4/2006_H10N4
A/MALLARD/SWEDEN/8004/2005_H10N4
A/MALLARD/SWEDEN/6039/2005_H10N4
A/MALLARD/SWEDEN/8023/2005_H10N4
A/RUDDY_TURNSTONE/NETHERLANDS/1/2008_H10N4
A/RUDDY_TURNSTONE/NETHERLANDS/8/2008_H10N4
A/RUDDY_TURNSTONE/NETHERLANDS/5/2008_H10N4
A/MALLARD/SWEDEN/51671/2006_H8N4
A/MALLARD/SWEDEN/51156/2006_H8N4
A/MALLARD/SWEDEN/50055/2006_H8N4
A/MALLARD/SWEDEN/58256/2006_H8N4
A/MALLARD/SWEDEN/4258/2004_H10N4
A/MALLARD/SWEDEN/4401/2004_H10N4
A/DUCK/ITALY/268302/2004_H10N4
A/MALLARD/SWEDEN/5389/2005_H8N4
A/MALLARD/NETHERLANDS/30/2006_H1N4
A/ANAS_CRECCA/SPAIN/1459/2008_H8N4
A/ANAS_PLATHYRHYNCHOS/SPAIN/1502/2008_H8N4
A/MALLARD/SWEDEN/64476/2007_H10N4
A/RUDDY_TURNSTONE/NETHERLANDS/1/2009_H10N4
A/RUDDY_TURNSTONE/NETHERLANDS/2/2009_H10N4
A/ANAS_PLATHYRHYNCHOS/SPAIN/1495/2008_H10N4
A/MALLARD/SWEDEN/59475/2007_H8N4
A/MALLARD/SWEDEN/133546/2011_H10N4
A/EURASIAN_WIGEON/NETHERLANDS/2/2008_H7N4
A/MALLARD/NETHERLANDS/11/2006_H8N4
A/NORTHERN_SHOVELER/NETHERLANDS/1/2006_H8N4
A/MALLARD/NETHERLANDS/13/2006_H8N4
A/MALLARD/SWEDEN/8005/2005_H8N4
A/MALLARD/SWEDEN/7996/2005_H8N4
A/COMMON_TEAL/NETHERLANDS/1/2005_H8N4
A/MALLARD/SWEDEN/51619/2006_H10N4
A/MALLARD/SWEDEN/51548/2006_H10N4
A/TEAL/CHANY/7119/2008_H15N4
A/DUCK/THAILAND/CU-9754C/2010_H7N4
A/DUCK/THAILAND/CU-9744C_/2010_H7N4
A/MALLARD/REPUBLIC_OF_GEORGIA/12/2011_H10N4
A/CHICKEN/NETHERLANDS/10007882/2010_H7N4
A/CHICKEN/NETHERLANDS/10009401/2010_H8N4
A/MALLARD/LITHUANIA/EMC-1/2010_H10N4
A/MALLARD/NETHERLANDS/30/2011_H6N4
A/CHICKEN/NETHERLANDS/11004004/2011_H8N4
A/MALLARD/SWEDEN/100546/2009_H8N4
A/MALLARD/LITHUANIA/EMC-1/2011_H10N4
A/MALLARD/SWEDEN/101165/2009_H8N4
A/MALLARD/SWEDEN/99377/2009_H8N4
0.05

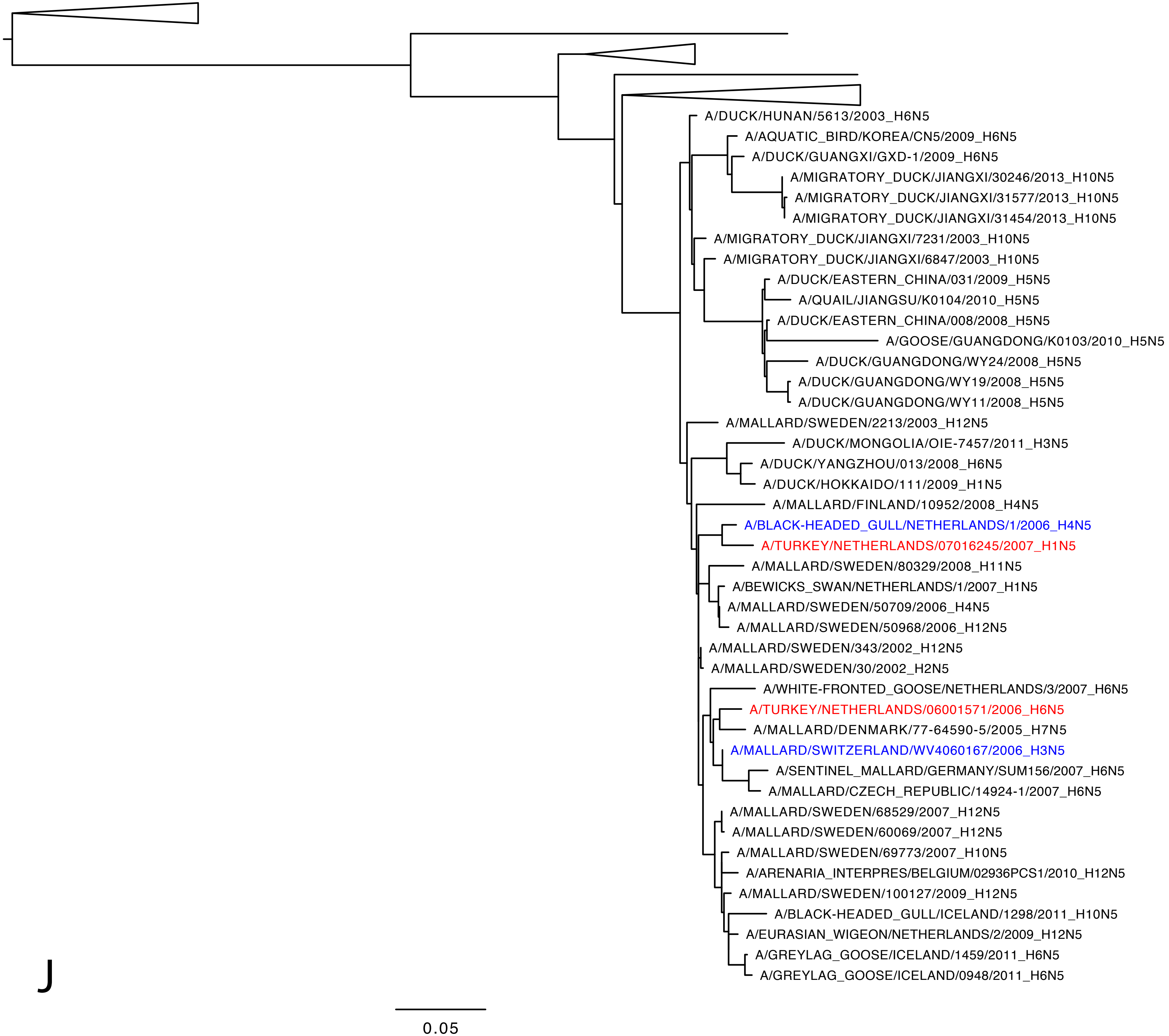
A/DUCK/HUNAN/5613/2003_H6N5
A/AQUATIC_BIRD/KOREA/CN5/2009_H6N5
A/DUCK/GUANGXI/GXD-1/2009_H6N5
A/MIGRATORY_DUCK/JIANGXI/30246/2013_H10N5
A/MIGRATORY_DUCK/JIANGXI/31577/2013_H10N5
A/MIGRATORY_DUCK/JIANGXI/31454/2013_H10N5
A/MIGRATORY_DUCK/JIANGXI/7231/2003_H10N5
A/MIGRATORY_DUCK/JIANGXI/6847/2003_H10N5
A/DUCK/EASTERN_CHINA/031/2009_H5N5
A/QUAIL/JIANGSU/K0104/2010_H5N5
A/DUCK/EASTERN_CHINA/008/2008_H5N5
A/GOOSE/GUANGDONG/K0103/2010_H5N5
A/DUCK/GUANGDONG/WY24/2008_H5N5
A/DUCK/GUANGDONG/WY19/2008_H5N5
A/DUCK/GUANGDONG/WY11/2008_H5N5
A/MALLARD/SWEDEN/2213/2003_H12N5
A/DUCK/MONGOLIA/OIE-7457/2011_H3N5
A/DUCK/YANGZHOU/013/2008_H6N5
A/DUCK/HOKKAIDO/111/2009_H1N5
A/MALLARD/FINLAND/10952/2008_H4N5
A/BLACK-HEADED_GULL/NETHERLANDS/1/2006_H4N5
A/TURKEY/NETHERLANDS/07016245/2007_H1N5
A/MALLARD/SWEDEN/80329/2008_H11N5
A/BEWICKS_SWAN/NETHERLANDS/1/2007_H1N5
A/MALLARD/SWEDEN/50709/2006_H4N5
A/MALLARD/SWEDEN/50968/2006_H12N5
A/MALLARD/SWEDEN/343/2002_H12N5
A/MALLARD/SWEDEN/30/2002_H2N5
A/WHITE-FRONTED_GOOSE/NETHERLANDS/3/2007_H6N5
A/TURKEY/NETHERLANDS/06001571/2006_H6N5
A/MALLARD/DENMARK/77-64590-5/2005_H7N5
A/MALLARD/SWITZERLAND/WV4060167/2006_H3N5
A/SENTINEL_MALLARD/GERMANY/SUM156/2007_H6N5
A/MALLARD/CZECH_REPUBLIC/14924-1/2007_H6N5
A/MALLARD/SWEDEN/68529/2007_H12N5
A/MALLARD/SWEDEN/60069/2007_H12N5
A/MALLARD/SWEDEN/69773/2007_H10N5
A/ARENARIA_INTERPRES/BELGIUM/02936PCS1/2010_H12N5
A/MALLARD/SWEDEN/100127/2009_H12N5
A/BLACK-HEADED_GULL/ICELAND/1298/2011_H10N5
A/EURASIAN_WIGEON/NETHERLANDS/2/2009_H12N5
A/GREYLAG_GOOSE/ICELAND/1459/2011_H6N5
A/GREYLAG_GOOSE/ICELAND/0948/2011_H6N5
J
0.05

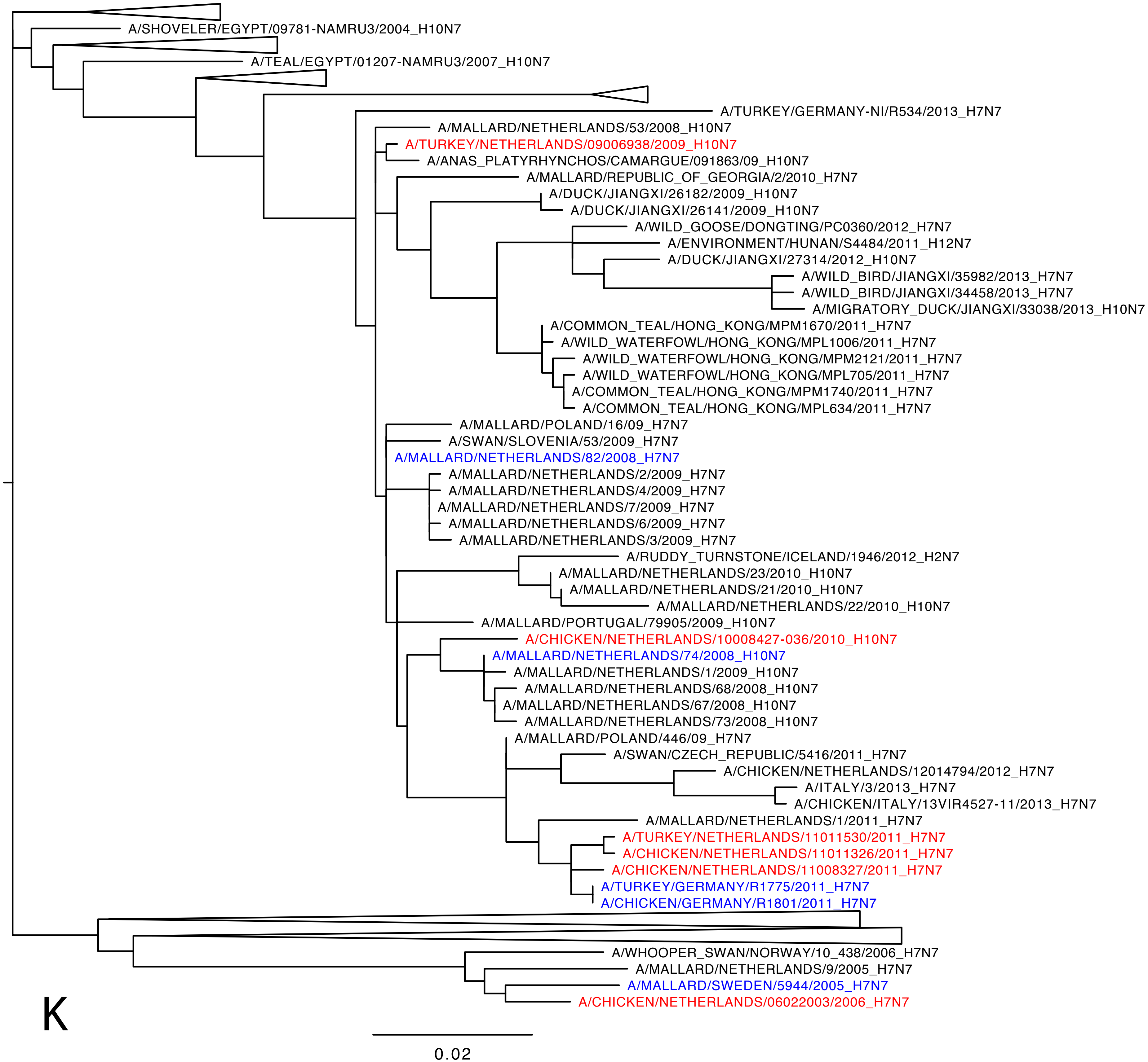

A/SHOVELER/EGYPT/09781-NAMRU3/2004_H10N7
A/TEAL/EGYPT/01207-NAMRU3/2007_H10N7
A/TURKEY/GERMANY-NI/R534/2013_H7N7
A/MALLARD/NETHERLANDS/53/2008_H10N7
A/TURKEY/NETHERLANDS/09006938/2009_H10N7
A/ANAS_PLATYRHYNCHOS/CAMARGUE/091863/09_H10N7
A/MALLARD/REPUBLIC_OF_GEORGIA/2/2010_H7N7
A/DUCK/JIANGXI/26182/2009_H10N7
A/DUCK/JIANGXI/26141/2009_H10N7
A/WILD_GOOSE/DONGTING/PC0360/2012_H7N7
A/ENVIRONMENT/HUNAN/S4484/2011_H12N7
A/DUCK/JIANGXI/27314/2012_H10N7
A/WILD_BIRD/JIANGXI/35982/2013_H7N7
A/WILD_BIRD/JIANGXI/34458/2013_H7N7
A/MIGRATORY_DUCK/JIANGXI/33038/2013_H10N7
A/COMMON_TEAL/HONG_KONG/MPM1670/2011_H7N7
A/WILD_WATERFOWL/HONG_KONG/MPL1006/2011_H7N7
A/WILD_WATERFOWL/HONG_KONG/MPM2121/2011_H7N7
A/WILD_WATERFOWL/HONG_KONG/MPL705/2011_H7N7
A/COMMON_TEAL/HONG_KONG/MPM1740/2011_H7N7
A/COMMON_TEAL/HONG_KONG/MPL634/2011_H7N7
A/MALLARD/POLAND/16/09_H7N7
A/SWAN/SLOVENIA/53/2009_H7N7
A/MALLARD/NETHERLANDS/82/2008_H7N7
A/MALLARD/NETHERLANDS/2/2009_H7N7
A/MALLARD/NETHERLANDS/4/2009_H7N7
A/MALLARD/NETHERLANDS/7/2009_H7N7
A/MALLARD/NETHERLANDS/6/2009_H7N7
A/MALLARD/NETHERLANDS/3/2009_H7N7
A/RUDDY_TURNSTONE/ICELAND/1946/2012_H2N7
A/MALLARD/NETHERLANDS/23/2010_H10N7
A/MALLARD/NETHERLANDS/21/2010_H10N7
A/MALLARD/NETHERLANDS/22/2010_H10N7
A/MALLARD/PORTUGAL/79905/2009_H10N7
A/CHICKEN/NETHERLANDS/10008427-036/2010_H10N7
A/MALLARD/NETHERLANDS/74/2008_H10N7
A/MALLARD/NETHERLANDS/1/2009_H10N7
A/MALLARD/NETHERLANDS/68/2008_H10N7
A/MALLARD/NETHERLANDS/67/2008_H10N7
A/MALLARD/NETHERLANDS/73/2008_H10N7
A/MALLARD/POLAND/446/09_H7N7
A/SWAN/CZECH_REPUBLIC/5416/2011_H7N7
A/CHICKEN/NETHERLANDS/12014794/2012_H7N7
A/ITALY/3/2013_H7N7
A/CHICKEN/ITALY/13VIR4527-11/2013_H7N7
A/MALLARD/NETHERLANDS/1/2011_H7N7
A/TURKEY/NETHERLANDS/11011530/2011_H7N7
A/CHICKEN/NETHERLANDS/11011326/2011_H7N7
A/CHICKEN/NETHERLANDS/11008327/2011_H7N7
A/TURKEY/GERMANY/R1775/2011_H7N7
A/CHICKEN/GERMANY/R1801/2011_H7N7
A/WHOOPER_SWAN/NORWAY/10_438/2006_H7N7
A/MALLARD/NETHERLANDS/9/2005_H7N7
A/MALLARD/SWEDEN/5944/2005_H7N7
A/CHICKEN/NETHERLANDS/06022003/2006_H7N7
K
0.02

S2 Figure. Maximum Likelihood trees of influenza A virus HA and NA subtypes as detected in poultry, the Netherlands, 2006 - 2011. H1 (A), H6 (B), H7 (C), H8 (D), H9 (E), H10 (F), N1 (G), N2 (H), N4 (I), N5 (J) and N7 (K). Red indicates influenza viruses isolated from poultry in the Netherlands within this study period and blue indicates the genetically closest influenza virus isolated from wild birds.
