# Supplementary material for: Discordant detection of avian influenza virus subtypes in time and space between poultry and wild birds; Towards improvement of surveillance programs
Source: PLoS One. 2017 Mar 9;12(3):e0173470. doi: 10.1371/journal.pone.0173470 (PMC5344487; doi:10.1371/journal.pone.0173470)
Supplement: S1 Table — (PDF) [file pone.0173470.s003.pdf]

S1 Table. The low pathogenic avian influenza viruses (LPAIV) and the accession numbers of the segments used in this study as listed in online databases GenBank and GISAID EpiFlu.

Viral Name	Source	Accession number HA segment	Accession number NA segment
A/White-fronted_goose/Netherlands/1/2007_H1N1	GenBank	KR862410	KR862542
A/Bean_goose/Netherlands/1/2007_H1N1	GenBank	KR862411	KR862543
A/Mallard/Netherlands/26/2006_H1N1	GenBank	KR862413	
A/Mallard/Netherlands/64/2006_H1N1	GenBank	KR862414	KR862551
A/Mallard/Netherlands/23/2006_H1N1	GenBank	KR862412	
A/Bewicks_swan/Netherlands/6/2006_H1N1	GenBank	KR862416	
A/Pink-footed_goose/Belgium/EMC-1/2007_H1N1	GenBank	KR862369	KR862390
A/Eurasian_wigeon/Netherlands/2/2007_H1N1	GenBank	KR862417	
A/Black-headed_gull/Netherlands/1/2008_H1N3	GenBank	KR862418	
A/White-fronted_goose/Netherlands/4/2011_H1N1	GenBank	KR862419	KR862558
A/Common_teal/Netherlands/3/2005_H1N1	GenBank	KR862408	KR862539
A/Barnacle_goose/Netherlands/1/2006_H1N1	GenBank	KR862409	KR862540
A/Eurasian_wigeon/Netherlands/6/2007_H1N1	GenBank	KR862420	KR862560
A/Mallard/Netherlands/16/2009_H1N1	GenBank	KR862421	KR862577
A/Mallard/Netherlands/53/2010_H1N1	GenBank	KR862422	KR862581
A/Mallard/Netherlands/6/2012_H1N1	GenBank	KR862423	KR862582
A/Mallard/Netherlands/7/2012_H1N1	GenBank	KR862424	KR862586
A/Mallard/Netherlands/11/2012_H1N1	GenBank	KR862426	KR862590
A/Mallard/Netherlands/51/2010_H1N1	GenBank	KR862427	KR862595
A/Northern_pintail/Egypt/EMC-2/2012_H1N1	GenBank	KR862370	KR862392
A/Northern_pintail/Egypt/EMC-3/2012_H1N1	GenBank	KR862371	
A/Chicken/Netherlands/11009919/2011_H1N1	GenBank	KY676319	KY676335
A/Teal/Egypt/20431-NAMRU3/2003_H1N2	GISAID	EPI372275	
A/Shoveler/Egypt/00134-NAMRU3/2005_H1N1	GISAID	EPI372331	
A/Shoveler/Egypt/14029-NAMRU3/2006_H1N1	GISAID	EPI372378	EPI372377
A/Teal/Egypt/01351-NAMRU3/2007_H1N1	GISAID	EPI372466	EPI372465
A/Teal/Egypt/00677-NAMRU3/2004_H1N1	GISAID	EPI372528	
A/Goose/Italy/6117/2004_H1N1	GISAID	EPI178520	EPI178522
A/Mallard/Germany/R2843/06_H1N1	GISAID	EPI222781	
A/Mallard/Germany-RP/R193/09_H1N1	GISAID	EPI248501	EPI248500
A/Wild_duck/Germany/WV30/06_H1N1	GISAID	EPI248514	EPI248512
A/Mallard/Germany/WV355/07_H1N1	GISAID	EPI248519	EPI248517
A/Wild_duck/Germany-NW/R04/08_H1N1	GISAID	EPI248521	
A/Anas_platyrhynchos/Belgium/09-762/2008_H1N1	GISAID	EPI257212	EPI257214
A/Wild_duck/Korea/CSM38/2004b_H1N1	GISAID	EPI296244	
A/Duck/Italy/7686-11/10_H1N1	GISAID	EPI301849	
A/Pintail/Italy/2703-25/06_H1N1	GISAID	EPI301856	
A/Mallard/Italy/378-49/06_H1N1	GISAID	EPI301857	
A/Teal/Italy/6323-5/07_H1N1	GISAID	EPI301858	
A/Mallard/Italy/432-21/08_H1N1	GISAID	EPI301859	
A/Shoveler/Italy/6965-6/07_H1N3	GISAID	EPI301860	
A/Turkey/Netherlands/07016245/2007_H1N5	GenBank	KY676320	KY676341
A/Anas_crecca/Spain/1384/2007_H1N1	GenBank	FN386464	FN386472
A/Anas_crecca/Spain/1404/2007_H1N1	GenBank	FN386465	FN386474
A/Anas_plathyrhynchos/Spain/1365/2007_H1N1	GenBank	FN386463	FN386471
A/Bewicks_swan/Netherlands/1/2007_H1N5	GenBank	CY076976	CY076978
A/Chicken/Guangxi/GXc-1/2011_H1N2	GenBank	KF013910	
A/Common_goldeneye/Mongolia/1216/2010_H1N1	GenBank	KF501071	KF667692
A/Common_goldeneye/Mongolia/1238/2010_H1N1	GenBank	KF501070	KF667690
A/Common_teal/Netherlands/10/2000_H1N1	GenBank	CY060178	CY060180
A/Duck/Guangxi/GXd-1/2011_H1N2	GenBank	KF013918	
A/Duck/Guangxi/GXd-2/2012_H1N2	GenBank	KF013934	
A/Duck/Guangxi/GXd-4/2011_H1N2	GenBank	KF013926	
A/Duck/Hebei/843/2005_H1N2	GenBank	FJ536843	
A/Duck/Hokkaido/111/2009_H1N5	GenBank	AB560963	AB560964
A/Duck/Hokkaido/327/2009_H1N3	GenBank	AB560965	
A/Duck/Italy/1447/2005_H1N1	GenBank	HF563054	
A/Duck/Italy/281904/2006_H1N1	GenBank	FJ432770	
A/Duck/Italy/69238/2007_H1N1	GenBank	FJ432754	
A/Duck/Korea/372/2009_H1N1	GenBank	KJ764732	KJ764734
A/Duck/Korea/U11-1/2007_H1N2	GenBank	KJ764772	
A/Duck/Korea/U11/2007_H1N2	GenBank	HQ014832	
A/Duck/Korea/U14/2007_H1N3	GenBank	HQ014840	
A/Duck/Nanjing/19/2010_H1N3	GenBank	HQ336713	
A/Duck/Nanjing/20/2010_H1N3	GenBank	HQ336721	
A/Duck/Shimane/188/1999_H1N1	GenBank	CY091592	CY091594
A/Duck/Taiwan/DC167/2010_H1N3	GenBank	KC693623	

Viral Name	Source	Accession number HA segment	Accession number NA segment
A/Duck/Thailand/CU-11869C/2011_H1N9	GenBank	KJ161957	
A/Duck/Tsukuba/718/2005_H1N1	GenBank	AB670330	AB472014
A/Duck/Zhejiang/0224-6/2011_H1N2	GenBank	JN605372	
A/Duck/Zhejiang/0607-13/2011_H1N2	GenBank	JN605373	
A/Duck/Zhejiang/0611-15/2011_H1N3	GenBank	JN716320	
A/Duck/Zhejiang/0611-17/2011_H1N3	GenBank	JN605375	
A/Duck/Zhejiang/0611-24/2011_H1N3	GenBank	JN716323	
A/Duck/Zhejiang/0611-8/2011_H1N3	GenBank	JN605374	
A/Duck/Zhejiang/473/2013_H1N4	GenBank	KF357774	
A/Duck/Zhejiang/475/2013_H1N4	GenBank	KF357775	
A/Duck/Zhejiang/476/2013_H1N4	GenBank	KF357776	
A/Duck/Zhejiang/477/2013_H1N4	GenBank	KF357777	KF357766
A/Egyptian_goose/South_Africa/AI1448/2007_H1N8	GenBank	GQ404705	
A/Environment/Korea/CSM12/2007_H1N2	GenBank	KJ764740	
A/Environment/Korea/UPO218/2008_H1N6	GenBank	KJ764780	
A/Goose/Italy/296426/2003_H1N1	GenBank	FJ432778	FJ432780
A/Mallard/Bavaria/185-8/2008_H1N1	GenBank	HQ259224	
A/Mallard/Bavaria/42/2006_H1N1	GenBank	GU046744	GU046745
A/Mallard/Finland/14740/2008_H1N1	GenBank	KF183609	
A/Mallard/Korea/KNU_YP09/2009_H1N1	GenBank	HQ897965	
A/Mallard/Netherlands/10/1999_H1N8	GenBank	CY060206	
A/Mallard/Netherlands/30/2006_H1N4	GenBank	CY076897	CY076899
A/Mallard/Republic_of_Georgia/1/2012_H1N1	GenBank	CY185593	CY185595
A/Mallard/Republic_of_Georgia/11/2011_H1N1	GenBank	KC190172	
A/Mallard/Republic_of_Georgia/4/2010_H1N1	GenBank	CY185441	
A/Mallard/Republic_of_Georgia/4/2012_H1N1	GenBank	CY185641	CY185643
A/Mallard/Sanjiang/390/2007_H1N1	GenBank	CY077076	CY077078
A/Mallard/Sweden/104803/2009_H1N1	GenBank	JX566076	JX566260
A/Mallard/Sweden/3/2002_H1N2	GenBank	CY060268	CY060270
A/Mallard/Sweden/51588/2006_mixed	GenBank	CY164649	
A/Mallard/Sweden/51880/2006_mixed	GenBank	CY164144	
A/Mallard/Sweden/52209/2006_mixed	GenBank	CY164872	
A/Mallard/Sweden/52405/2006_mixed	GenBank	CY164156	
A/Mallard/Sweden/57/2003_H1N1	GenBank	CY060329	CY060330
A/Mallard/Sweden/98/2002_H1N6	GenBank	CY060410	
A/Mallard/Sweden/99769/2009_H1N1	GenBank	JX565989	
A/Mallard/Sweden/99818/2009_H1N1	GenBank	JX565992	
A/Mallard/Sweden/99842/2009_H1N1	GenBank	JX566000	
A/Mallard/Sweden/99857/2009_H1N1	GenBank	JX566013	
A/Mallard/Sweden/99859/2009_H1N2	GenBank	JX566015	
A/Muscovy_duck/Guangxi/GXd-3/2012_H1N2	GenBank	KF013942	
A/Ostrich/South_Africa/AI2887/2011_H1N2	GenBank	JX069105	
A/Pintail/Akita/1265/2008_H1N2	GenBank	AB546162	
A/Pintail/Aomori/1130/2008_H1N3	GenBank	AB546180	
A/Pintail/Aomori/422/2007_H1N1	GenBank	AB546149	AB546151
A/Pintail/Aomori/794/2008_H1N1	GenBank	AB546153	AB546155
A/Pintail/Miyagi/1472/2008_H1N1	GenBank	AB546157	AB546159
A/Pintail/Shimane/324/98_H1N9	GenBank	AB274304	
A/Sparrow/Guangxi/GXs-1/2012_H1N2	GenBank	KF013902	
A/Swan/Hokkaido/55/1996_H1N1	GenBank	AB271115	AB271116
A/Swine/Hong_Kong/644/1993_H1N1	GenBank	CY085009	
A/Tufted_duck/Mongolia/1409/2010_H1N1	GenBank	KF501056	
A/WDK/JX/12416/2005_H1N1	GenBank	FN436023	
A/Wild_duck/Guangdong/520/2001_H1N9	GenBank	KF258943	
A/Wild_duck/Korea/CSM38/2004a_H1N1	GenBank	HQ014744	
A/Wild_duck/Korea/CW09/2005_H1N1	GenBank	HQ014776	
A/Wild_duck/Korea/ESD48/2006_H1N1	GenBank	HQ014816	
A/Wild_duck/Korea/HDR02/2005_H1N1	GenBank	HQ014768	
A/Wild_duck/Korea/PJ25/2006_H1N3	GenBank	HQ014800	
A/Wild_duck/Korea/PSC30-20/2010_H1N1	GenBank	KJ764764	
A/Wild_duck/Korea/SH13/2006_H1N1	GenBank	HQ014784	
A/Wild_duck/Korea/SH14/2006_H1N3	GenBank	HQ014792	
A/Wild_duck/Korea/SH29/2006_H1N3	GenBank	HQ014808	
A/Wild_duck/Korea/SH60/2004_H1N1	GenBank	HQ014760	
A/Wild_duck/Korea/UP122/2007_H1N1	GenBank	HQ014824	
A/Wild_duck/Korea/YS44/2004_H1N2	GenBank	HQ014752	
A/Wild_waterfowl/Dongting/C2383/2012_H1N2	GenBank	KF874481	
A/Avian/Germany-BB/R2859/2009_H6	GISAID	EPI339183	
A/Goose/Germany-BB/R1625/2008_H6	GISAID	EPI279941	
A/Ringed_teal/Germany-NRW/R641/2008_H6	GISAID	EPI279938	

Viral Name	Source	Accession number HA segment	Accession number NA segment
A/Wild_bird/Germany-HH/R1501/2008_H6	GISAID	EPI279939	
A/Wild_bird/Germany-MV/R1511/2008_H6	GISAID	EPI279940	
A/Environment/California/NWRC182841-09/2006_H6N1	GISAID	EPI406091	
A/Environment/California/NWRC183200-14/2006_H6N1	GISAID	EPI406099	
A/Environment/California/NWRC183274-04/2006_H6N1	GISAID	EPI406103	
A/Green-winged_teal/Nova_Scotia/14917/2005_H6N1	GISAID	EPI327397	
A/Larus_argentatus/Belgium/02936pcs3/2010_H6N1	GISAID	EPI345428	EPI345427
A/Northern_shoveler/California/HKWF115/2007_H6N1	GISAID	EPI154816	
A/Duck/Germany-MV/R871/2010_H6N2	GISAID	EPI339182	
A/Green-winged_teal/Minnesota/Sg-00199/2007_H6N2	GISAID	EPI298290	
A/Green-winged_teal/Minnesota/Sg-00222/2007_H6N2	GISAID	EPI298322	
A/Mule_duck/Bulgaria/156/2010_H6N2	GISAID	EPI574180	
A/Mule_duck/Bulgaria/173/2009_H6N2	GISAID	EPI574206	
A/Shoveler/Egypt/13251-NAMRU3/2006_H6N2	GISAID	EPI372371	EPI372370
A/Teal/Egypt/13203-NAMRU3/2006_H6N2	GISAID	EPI372386	EPI372385
A/Turkey/Germany/R617/2007_H6N2	GISAID	EPI317612	
A/Wild_goose/Germany-BB/R2329/2008_H6N2	GISAID	EPI397608	EPI397607
A/Environment/North_Carolina/NWRC183941-06/2006_H6N5	GISAID	EPI406114	
A/Sentinel_mallard/Germany/Sum156/2007_H6N5	GISAID	EPI397610	EPI397609
A/Duck/Germany-NW/R2185/2006_H6N8	GISAID	EPI397606	
A/Environment/California/NWRC183200-06/2006_H6N8	GISAID	EPI406098	
A/Goose/Germany/R1767/2007_H6N8	GISAID	EPI416259	
A/Mallard/Germany-BY/R1353/2010_H6N8	GISAID	EPI339180	
A/Mule_duck/Bulgaria/365/2010_H6N8	GISAID	EPI574207	
A/Mute_swan/Germany/R2927/2007_H6N8	GISAID	EPI185339	
A/Pink-footed_goose/Iceland/0987/2011_H6N8	GISAID	EPI476116	
A/Mule_duck/Bulgaria/175/2009_H6	GISAID	EPI574252	
A/Mule_duck/Bulgaria/181/2010_H6	GISAID	EPI574247	
A/Mallard/Netherlands/78/2006_H6N8	GenBank	KR862436	
A/Eurasian_wigeon/Netherlands/1/2006_H6N2	GenBank	KR862437	KR862610
A/Mallard/Netherlands/40/2006_H6N8	GenBank	KR862438	
A/White-fronted_goose/Netherlands/3/2007_H6N5	GenBank	KR862439	KR862694
A/Mallard/Netherlands/5/2008_H6N8	GenBank	KR862440	
A/Bewicks_swan/Netherlands/8/2009_H6N8	GenBank	KR862441	
A/White-fronted_goose/Netherlands/11/2009_H6N1	GenBank	KR862443	KR862556
A/White-fronted_goose/Netherlands/4/2010_H6N8	GenBank	KR862447	
A/White-fronted_goose/Netherlands/9/2009_H6N2	GenBank	KR862444	KR862636
A/Barnacle_goose/Netherlands/1/2010_H6N8	GenBank	KR862445	
A/White-fronted_goose/Netherlands/1/2010_H6N1	GenBank	KR862446	KR862557
A/Mallard/Netherlands/2/2004_H6N8	GenBank	KR862428	
A/Common_teal/Netherlands/4/2005_H6N2	GenBank	KR862430	KR862604
A/White-fronted_goose/Netherlands/1/2006_H6N2	GenBank	KR862431	KR862606
A/Eurasian_wigeon/Netherlands/6/2005_H6N2	GenBank	KR862432	KR862607
A/White-fronted_goose/Netherlands/1/2005_H6N8	GenBank	KR862433	
A/White-fronted_goose/Netherlands/3/2006_H6N8	GenBank	KR862434	
A/White-fronted_goose/Netherlands/2/2008_H6N8	GenBank	KR862455	
A/White-fronted_goose/Netherlands/2/2007_H6N8	GenBank	KR862456	
A/Mallard/Netherlands/8/2008_H6N1	GenBank	KR862457	KR862563
A/Mallard/Netherlands/42/2008_H6N1	GenBank	KR862459	KR862569
A/Eurasian_wigeon/Netherlands/1/2008_H6N1	GenBank	KR862461	KR862573
A/Common_teal/Netherlands/3/2008_H6N1	GenBank	KR862462	KR862575
A/Mallard/Netherlands/31/2008_H6N1	GenBank	KR862460	KR862572
A/Mallard/Netherlands/17/2009_H6N8	GenBank	KR862463	
A/Mallard/Netherlands/15/2011_H6N8	GenBank	KR862465	
A/Mallard/Netherlands/20/2011_H6N8	GenBank	KR862466	
A/White-fronted_goose/Netherlands/6/2009_H6N8	GenBank	KR862470	
A/Mallard/Netherlands/18/2010_H6N8	GenBank	KR862471	
A/Chicken/Netherlands/10010413/2010_H6N1	GenBank	KY676321	KY676333
A/Turkey/Netherlands/06001571/2006_H6N5	GenBank	KY676322	KY676342
A/American_wigeon/California/8910/2008_H6N1	GenBank	CY094405	
A/American_wigeon/California/HKWF371/2007_H6N5	GenBank	CY032704	
A/American_wigeon/California/HKWF42/2007_H6N1	GenBank	CY033420	
A/American_wigeon/California/HKWF541/2007_H6N5	GenBank	CY033436	
A/Anas_discors/New_Mexico/A00629390/2008_H6N1	GenBank	KF636135	
A/Anas_discors/New_Mexico/A00706363/2008_H6N1	GenBank	KF569944	
A/Aquatic_bird/Korea/W69/2005_H6N5	GenBank	CY098532	CY098534
A/Avian/Japan/8KI0135/2008_H6N5	GenBank	CY079243	CY079245
A/Avian/Japan/8KI0195/2008_H6N8	GenBank	CY079211	
A/Barnacle_goose/Netherlands/1/2005_H6N2	GenBank	CY041386	CY041388
A/Bewicks_swan/Netherlands/1/2005_H6N2	GenBank	DQ822190	DQ822192

Viral Name	Source	Accession number HA segment	Accession number NA segment
A/Bewicks_swan/Netherlands/2/2005_H6N8	GenBank	DQ822198	
A/Black-headed_gull/Netherlands/1/2005_H6N8	GenBank	CY041378	
A/Blue-winged_teal/Ohio/1386/2005_H6N2	GenBank	CY081380	
A/Blue-winged_teal/Ohio/1387/2005_H6N2	GenBank	CY021861	
A/Blue-winged_teal/Ohio/1918/2006_H6N5	GenBank	CY095616	
A/Common_gull/Norway/10_1602/2009_H6N8	GenBank	HE802707	
A/Common_teal/Netherlands/2/2005_H6N8	GenBank	CY041370	
A/Duck/Eastern_China/1/2008_H6N1	GenBank	JF965144	
A/Duck/Eastern_China/34/2005_H6N1	GenBank	JF965160	JF965335
A/Duck/Eastern_China/50/2002_H6N8	GenBank	JF965168	
A/Duck/Eastern_China/52/2002_H6N2	GenBank	JF965170	
A/Duck/Eastern_China/53/2002_H6N2	GenBank	JF965171	
A/Duck/Eastern_China/54/2002_H6N2	GenBank	JF965172	
A/Duck/Eastern_China/55/2003_H6N2	GenBank	JF965173	
A/Duck/Eastern_China/57/2003_H6N2	GenBank	JF965174	
A/Duck/Eastern_China/58/2003_H6N2	GenBank	JF965175	
A/Duck/Eastern_China/59/2003_H6N2	GenBank	JF965176	
A/Duck/Eastern_China/60/2003_H6N2	GenBank	JF965177	
A/Duck/Guangxi/1074/2006_H6N2	GenBank	CY109226	
A/Duck/Guangxi/1157/2006_H6N2	GenBank	CY109234	
A/Duck/Guangxi/1248/2006_H6N2	GenBank	CY109242	
A/Duck/Guangxi/141/2005_H6N2	GenBank	HM144510	HM144680
A/Duck/Guangxi/1455/2004_H6N5	GenBank	HM144533	HM144703
A/Duck/Guangxi/1533/2007_H6N2	GenBank	CY109618	
A/Duck/Guangxi/1598/2005_H6N2	GenBank	HM144511	HM144681
A/Duck/Guangxi/2140/2006_H6N2	GenBank	CY109250	
A/Duck/Guangxi/2736/2006_H6N8	GenBank	CY109258	
A/Duck/Guangxi/3333/2006_H6N1	GenBank	CY109266	CY109268
A/Duck/Guangxi/3459/2005_H6N2	GenBank	HM144512	
A/Duck/Guangxi/585/2005_H6N5	GenBank	HM144536	HM144706
A/Duck/Guizhou/1084/2006_H6N2	GenBank	CY109290	CY109292
A/Duck/Guizhou/1426/2006_H6N1	GenBank	CY109298	CY109300
A/Duck/Guizhou/2492/2007_H6N1	GenBank	CY109658	CY109660
A/Duck/Guizhou/879/2006_H6N1	GenBank	CY109921	CY109922
A/Duck/Guizhou/888/2006_H6N5	GenBank	CY109282	CY109284
A/Duck/Hokkaido/120/2001_H6N2	GenBank	AB286875	
A/Duck/Hokkaido/228/2003_H6N8	GenBank	AB294219	
A/Duck/Hokkaido/W2/2004_H6N8	GenBank	AB450441	
A/Duck/Hunan/1469/2002_H6N8	GenBank	HM144552	
A/Duck/Hunan/177/2005_H6N1	GenBank	HM144392	HM144562
A/Duck/Hunan/2110/2006_H6N2	GenBank	CY109314	
A/Duck/Hunan/5613/2003_H6N5	GenBank	HM144532	HM144702
A/Duck/Hunan/573/2002_H6N2	GenBank	HM144461	
A/Duck/Hunan/748/2005_H6N5	GenBank	HM144537	HM144707
A/Duck/Shantou/7904/2006_H6N2	GenBank	CY109426	
A/Duck/Spain/539/2006_H6N8	GenBank	AM706353	
A/Duck/Spain/543/2006_H6N8	GenBank	AM706355	
A/Duck/Suphanburi/AI157/2005_H6N1	GenBank	JQ711490	JQ711488
A/Duck/Taiwan/WB459/04_H6N5	GenBank	DQ376651	DQ376723
A/Duck/Tsukuba/561/2006_H6N1	GenBank	AB669136	
A/Duck/Yunnan/3136/2006_H6N2	GenBank	CY109346	CY109348
A/Dunlin/Barrow/65/2005_H6N1	GenBank	EF655836	
A/Environment/California/NWRC184193-25/2006_H6	GenBank	CY122504	
A/Environment/Colorado/NWRC183938-30/2006_H6	GenBank	CY122496	
A/Environment/Delaware/NWRC186237-18/2007_H6	GenBank	CY122532	
A/Environment/Illinois/NWRC183983-24/2006_H6N2	GenBank	CY122500	
A/Environment/Lousiana/NWRC186275-18/2007_H6	GenBank	CY122535	
A/Environment/New_York/23857/2005_H6N8	GenBank	CY095204	
A/Environment/New_York/32072-2/2006_H6N8	GenBank	CY095196	
A/Environment/New_York/NWRC183209-12/2006_H6N1	GenBank	CY122484	
A/Eurasian_wigeon/Netherlands/4/2000_H6N2	GenBank	KF695262	
A/Eurasian_wigeon/Sweden/1/2004_H6N2	GenBank	CY041362	
A/Glaucous_gull/Alaska/44198-119/2006_H6N1	GenBank	HM060001	
A/Greylag_goose/Iceland/0911/2011_H6N8	GenBank	CY149468	
A/Greylag_goose/Iceland/0921/2011_H6N8	GenBank	CY149476	
A/Greylag_goose/Iceland/0948/2011_H6N5	GenBank	CY149500	CY149502
A/Greylag_goose/Iceland/0961/2011_H6N8	GenBank	CY149516	
A/Greylag_goose/Iceland/0976/2011_H6N8	GenBank	CY149524	
A/Greylag_goose/Iceland/0980/2011_H6N8	GenBank	CY149532	
A/Greylag_goose/Iceland/1459/2011_H6N5	GenBank	CY149444	CY149446

Viral Name	Source	Accession number HA segment	Accession number NA segment
A/Greylag_goose/Iceland/1474/2011_H6N8	GenBank	CY149452	
A/Greylag_goose/Iceland/1482/2011_H6N8	GenBank	CY149460	
A/Greylag_goose/Netherlands/4/1999_H6N1	GenBank	CY060198	CY060200
A/Gull/Moscow/3100/2006_H6N2	GenBank	EU152237	EU152239
A/Mallard_duck/Minnesota/Sg-00107/2007_H6N2	GenBank	CY034673	
A/Mallard/California/8212/2008_H6N1	GenBank	CY094165	
A/Mallard/California/8293/2008_H6N1	GenBank	CY094173	
A/Mallard/Czech_Republic/14924-1/2007_H6N5	GenBank	JF789626	JF789628
A/Mallard/Czech_Republic/15902-17K/2009_H6N2	GenBank	HQ244430	
A/Mallard/Czech_Republic/15962-1T/2010_H6N9	GenBank	JQ737237	
A/Mallard/Finland/13792/2007_H6N8	GenBank	KF183620	
A/Mallard/Hei_Longjiang/131/2006_H6N2	GenBank	EF634340	
A/Mallard/Jiangxi/10071/2005_H6N1	GenBank	HM144393	HM144563
A/Mallard/Jiangxi/10668/2005_H6N1	GenBank	HM144394	HM144564
A/Mallard/Jiangxi/12147/2005_H6N2	GenBank	HM144516	
A/Mallard/Jiangxi/13228/2005_H6N1	GenBank	HM144395	HM144565
A/Mallard/Jiangxi/227/2003_H6N1	GenBank	HM144388	HM144558
A/Mallard/Jiangxi/7787/2003_H6N1	GenBank	HM144390	HM144560
A/Mallard/Jiangxi/8264/2004_H6N2	GenBank	HM144489	HM144659
A/Mallard/Jiangxi/8341/2004_H6N5	GenBank	HM144534	HM144704
A/Mallard/Jiangxi/8346/2004_H6N5	GenBank	HM144535	HM144705
A/Mallard/Maryland/06OS196/2006_H6N2	GenBank	CY190307	
A/Mallard/Maryland/504/2006_mixed	GenBank	CY081404	
A/Mallard/Minnesota/Sg-00104/2007_H6N1	GenBank	CY078034	
A/Mallard/Minnesota/Sg-00105/2007_H6N1	GenBank	CY078042	
A/Mallard/Minnesota/Sg-00106/2007_H6N2	GenBank	CY078273	
A/Mallard/Minnesota/Sg-00167/2007_H6N1	GenBank	CY078114	
A/Mallard/Minnesota/Sg-00214/2007_H6N1	GenBank	CY035387	
A/Mallard/Minnesota/Sg-00223/2007_H6N1	GenBank	CY078249	
A/Mallard/Netherlands/11/2007_H6N5	GenBank	CY041402	CY041404
A/Mallard/Netherlands/16/99_H6N5	GenBank	AY684892	
A/Mallard/Netherlands/71/2006_H6N2	GenBank	CY041394	CY041396
A/Mallard/Ontario/15915/2005_H6N5	GenBank	CY095323	
A/Mallard/Republic_of_Georgia/13/2011_H6N2	GenBank	CY185577	CY185579
A/Mallard/SanJiang/113/2006_H6N2	GenBank	EU094473	EU094475
A/Mallard/SanJiang/151/2006_H6N2	GenBank	EF592495	
A/Mallard/Sweden/3463/2003_H6N2	GenBank	CY186267	CY186269
A/Mallard/Sweden/51984/2006_mixed	GenBank	CY164804	CY164807
A/Mallard/Sweden/52/2003_H6N2	GenBank	CY060316	CY060318
A/Mallard/Sweden/52593/2006_mixed	GenBank	CY164993	CY164996
A/Mallard/Sweden/54/2003_H6N1	GenBank	KF695223	KF695225
A/Mallard/Sweden/99817/2009_H6N2	GenBank	JX565991	JX566175
A/Mallard/Sweden/99847/2009_H6N2	GenBank	JX566005	
A/Mallard/Sweden/99850/2009_H6N2	GenBank	JX566006	
A/Mallard/Sweden/99854/2009_H6N2	GenBank	JX566010	
A/Mallard/Sweden/99934/2009_H6N2	GenBank	JX566024	
A/Mallard/Sweden/99966/2009_H6N2	GenBank	JX566028	
A/Mallard/Sweden/99983/2009_H6N2	GenBank	JX566029	
A/Mallard/Sweden/99985/2009_H6N2	GenBank	JX566030	
A/Mallard/Sweden/99999/2009_H6N2	GenBank	JX566032	JX566216
A/Mallard/Wisconsin/1534/2009_H6N8	GenBank	CY097277	
A/Muscovy_duck/France/09010/2009_H6N1	GenBank	JN860172	JN860174
A/Northern_pintail/Alaska/44202-143/2006_H6N1	GenBank	EU557517	
A/Northern_pintail/Alaska/44203-078/2006_H6N8	GenBank	EU557519	
A/Northern_pintail/Alaska/44204-158/2006_H6N4	GenBank	EU557520	
A/Northern_shoveler/California/9187/2008_H6N2	GenBank	CY094309	
A/Northern_shoveler/California/K138/2005_H6N2	GenBank	CY045343	
A/Pintail/Alberta/87/1993_H6N8	GenBank	CY127024	
A/Ring-necked_duck/California/K90/2005_H6N8	GenBank	CY043808	
A/Shorebird/Delaware_Bay/13/2004_H6N8	GenBank	CY127726	
A/Shorebird/Delaware_Bay/604/2008_H6N2	GenBank	CY127831	
A/Shorebird/Delaware_Bay/65/2004_H6N8	GenBank	CY127734	
A/Shorebird/Delaware_Bay/707/2009_H6N2	GenBank	CY127895	
A/Teal/Norway/10_476/2005_H6N2	GenBank	FM179757	
A/Turkey/France/09010-1/2009_H6N1	GenBank	JN860180	
A/Turkey/France/10-040/2010_H6N1	GenBank	JQ990779	JQ990781
A/Whitefronted_goose/Netherlands/1/1999_H6N1	GenBank	CY060431	
A/Whitefronted_goose/Netherlands/2/1999_H6N2	GenBank	CY060439	
A/Wild_duck/Jiangxi/8462/2006_H6N1	GenBank	CY109338	CY109340
A/Chicken/Netherlands/11004875/2011_H7N1	GenBank	KY676323	KY676334

Viral Name	Source	Accession number HA segment	Accession number NA segment
A/Duck/Turkey/55/Cetinkaya/49/2006_H7N1	GISAID	EPI346007	
A/Guinea_fowl/Italy/407/2008_H7N1	GISAID	EPI210104	
A/Mallard/Denmark/58-62-KLSV-119/09_H7N1	GISAID	EPI492308	
A/Mallard/Italy/3397-65/2008_H7N1	GISAID	EPI167297	
A/Mallard/Italy/6103-5/2007_H7N1	GISAID	EPI167296	
A/Mallard/Italy/731/09_H7N1	GISAID	EPI492522	
A/Mallard/Italy/794-18/2008_H7N1	GISAID	EPI167299	
A/Shoveler/Egypt/00597-NAMRU3/2004_H7N1	GISAID	EPI372283	EPI372282
A/Shoveler/Egypt/14879-NAMRU3/2006_H7N1	GISAID	EPI372363	EPI372362
A/Tadorna_tadorna/Belgium/3441-P3/2009_H7N1	GISAID	EPI360900	EPI360901
A/Teal/Italy/794-3/2008_H7N1	GISAID	EPI167298	
A/Chicken/Italy/2240/2003_H7N3	GISAID	EPI154960	
A/Chicken/Italy/2837-54/2007_H7N3	GISAID	EPI154980	
A/Chicken/Italy/2837-58/2007_H7N3	GISAID	EPI154981	
A/Chicken/Italy/8093/2002_H7N3	GISAID	EPI154966	
A/Guinea_fowl/Italy/1613/2003_H7N3	GISAID	EPI154959	
A/Mallard/Italy/1336/07_H7N3	GISAID	EPI167295	
A/Mallard/Italy/6103-12/2007_H7N3	GISAID	EPI154982	
A/Mallard/Italy/6104-14/2007_H7N3	GISAID	EPI167300	
A/Shoveler/Egypt/00017-NAMRU3/2007_H7N3	GISAID	EPI372450	
A/Shoveler/Egypt/00241-NAMRU3/2007_H7N3	GISAID	EPI372418	
A/Turkey/Italy/2963/2003_H7N3	GISAID	EPI243279	
A/Turkey/Italy/8307/2002_H7N3	GISAID	EPI154967	
A/Swan/Germany/R736/06_H7N4	GISAID	EPI492517	
A/Teal/Italy/11VIR-792/11_H7N6	GISAID	EPI492520	
A/Branta_canadensis/Belgium/13000-9-2/2010_H7N7	GISAID	EPI360902	
A/Chicken/Germany-NI/R874/2010_H7N7	GISAID	EPI302178	
A/Chicken/Germany/R1362/11_H7N7	GISAID	EPI492511	
A/Chicken/Netherlands/11008327/2011_H7N7	GenBank	KY676325	KY676346
A/Chicken/Netherlands/11011326/2011_H7N7	GenBank	KY676326	KY676347
A/Chicken/Netherlands/12014794/2012_H7N7	GISAID	EPI390921	EPI390922
A/Egyptian_goose/Egypt/05588-NAMRU3/2006_H7N7	GISAID	EPI372394	EPI372393
A/Mallard/Denmark/303878-1S/13_H7N7	GISAID	EPI492307	
A/Mallard/Italy/11VIR-540/11_H7N7	GISAID	EPI492519	
A/Mallard/Poland/01/08_H7N7	GISAID	EPI169422	EPI169423
A/Mallard/Poland/41/09_H7N7	GISAID	EPI211188	
A/Mallard/Poland/446/09_H7N7	GISAID	EPI254381	EPI254382
A/Pochard/Germany/R916/06_H7N7	GISAID	EPI492516	
A/Shoveler/Egypt/09864-NAMRU3/2004_H7N7	GISAID	EPI372323	EPI372322
A/Swan/Germany/R57/06_H7N7	GISAID	EPI492518	
A/Teal/Egypt/00835-NAMRU3/2004_H7N7	GISAID	EPI372307	
A/Turkey/Germany-NW/R655/2009_H7N7	GISAID	EPI356351	
A/Shoveler/Egypt/00215-NAMRU3/2007_H7N9	GISAID	EPI372410	
A/Mallard/Netherlands/82/2008_H7N7	GenBank	KR862473	KR862709
A/Bewicks_swan/Netherlands/7/2008_H7N1	GenBank	KR862474	KR862554
A/Mallard/Lithuania/EMC-2/2010_H7N2	GenBank	KR862374	
A/Mallard/Netherlands/60/2008_H7N1	GenBank	KR862477	KR862566
A/Mallard/Netherlands/61/2008_H7N1	GenBank		KR862567
A/Mallard/Netherlands/2/2009_H7N7	GenBank	KR862479	KR862717
A/Mallard/Netherlands/4/2009_H7N7	GenBank	KR862481	KR862719
A/Mallard/Netherlands/6/2009_H7N7	GenBank	KR862482	KR862720
A/Eurasian_wigeon/Netherlands/2/2008_H7N4	GenBank	KR862485	KR862692
A/Bean_goose/Netherlands/1/2009_H7N1	GenBank	KR862487	KR862576
A/Mallard/Netherlands/2/2010_H7N3	GenBank	KR862488	
A/Mallard/Netherlands/3/2010_H7N3	GenBank	KR862489	
A/Mallard/Netherlands/1/2011_H7N7	GenBank	KR862490	KR862726
A/Mallard/Netherlands/43/2011_H7N1	GenBank	KR862491	
A/Northern_shoveler/Egypt/EMC-1/2012_H7N1	GenBank	KR862376	KR862391
A/Mallard/Netherlands/5/2010_H7N3	GenBank	KR862500	
A/Turkey/Netherlands/11011530/2011_H7N7	GenBank	KY676327	KY676348
A/Chicken/Netherlands/10007882/2010_H7N4	GenBank	KY676324	KY676338
A/Anas_crecca/Spain/1460/2008_H7N9	GenBank	HQ244407	
A/Anas_platyrhynchos/Spain/1877/2009_H7N2	GenBank	KP636486	
A/Chicken/England/1158-11406/2008_H7N7	GenBank	FJ476173	
A/Chicken/England/4054/2006_H7N3	GenBank	EF467826	
A/Chicken/England/4266/2006_H7N3	GenBank	EF467825	
A/Chicken/Italy/1067/99_H7N1	GenBank	AJ584647	
A/Chicken/Italy/1082/1999_H7N1	GenBank	CY022677	CY022679
A/Chicken/Italy/1279/1999_H7N1	GenBank	CY099597	
A/Chicken/Italy/12rs206-19/1999_H7N1	GenBank	KF493039	

Viral Name	Source	Accession number HA segment	Accession number NA segment
A/Chicken/Italy/12rs206-3/1999_H7N1	GenBank	KF492993	
A/Chicken/Italy/12rs206-4/1999_H7N1	GenBank	KF493027	
A/Chicken/Italy/12rs206-5/1999_H7N1	GenBank	KF493029	
A/Chicken/Italy/1391/1999_H7N1	GenBank	CY095514	
A/Chicken/Italy/13VIR4527-11/2013_H7N7	GISAID	EPI677999	EPI677998
A/Chicken/Italy/270638/02_H7N3	GenBank	EU158111	
A/Chicken/Italy/4616/2003_H7N3	GenBank	CY095522	
A/Chicken/Italy/682/2003_H7N3	GenBank	CY034750	
A/Chicken/Netherlands/06022003/2006_H7N7	GenBank	HE802065	KY676343
A/Chicken/Wales/1306/2007_H7N2	GenBank	EF675618	
A/Duck/Denmark/53-147-8/2008_H7N1	GenBank	GQ401157	GQ401158
A/Duck/Italy/4609/2003_H7N2	GenBank	CY031028	
A/Duck/Italy/4692-9/2004_H7	GenBank	CY095570	
A/Duck/Mongolia/47/2001_H7N1	GenBank	AB268557	AB302788
A/Duck/Mongolia/720/2007_H7N6	GenBank	AB450448	
A/Duck/Yunnan/87/2007_H7N6	GenBank	KF258991	
A/Garganey/Crimea/2027/2008_H7N8	GenBank	GU228596	
A/Goose/Czech_Republic/1848-K9/2009_H7N9	GenBank	GU060482	
A/Guinea_fowl/Italy/266184/02_H7N3	GenBank	EU158110	
A/Italy/3/2013_H7N7	GenBank	KF918337	KF918339
A/Mallard/Italy/199/01_H7N3	GenBank	EU158109	
A/Mallard/Italy/250/02_H7N1	GenBank	EU158105	
A/Mallard/Italy/299/05_H7N7	GenBank	EU158104	
A/Mallard/Italy/43/01_H7N3	GenBank	AY586410	
A/Mallard/Netherlands/12/2000_H7N3	GenBank	KF695239	
A/Mallard/Netherlands/22/2007_H7N1	GenBank	CY043840	CY043842
A/Mallard/Netherlands/29/2006_H7N2	GenBank	CY043832	CY043834
A/Mallard/Netherlands/33/2006_H7N8	GenBank	CY041410	
A/Mallard/Netherlands/9/2005_H7N7	GenBank	CY077008	CY077010
A/Mallard/Republic_of_Georgia/2/2010_H7N7	GenBank	CY185425	CY185427
A/Mallard/Republic_of_Georgia/3/2010_H7N3	GenBank	CY185433	
A/Mallard/Sweden/100993/2008_H7N7	GenBank	FJ803198	FJ803196
A/Mallard/Sweden/1985/2003_H7N7	GenBank	CY183336	CY183338
A/Mallard/Sweden/2051/2003_H7N7	GenBank	CY184568	
A/Mallard/Sweden/3269/2003_H7N7	GenBank	CY184576	CY184578
A/Mallard/Sweden/5994/2005_H7N7	GenBank	CY183409	CY183411
A/Mallard/Sweden/S90597/2005_H7N7	GenBank	FJ803182	FJ803175
A/Mute_swan/Hungary/5973/2007_H7N7	GenBank	GQ240813	GQ240815
A/Quail/Italy/3347/2004_H7N3	GenBank	CY020613	
A/Quail/Italy/4610/2003_H7N2	GenBank	CY021509	
A/Shoveler/Italy/2698-27/2006_H7N7	GenBank	CY095600	
A/Shoveler/Italy/2698-3/2006_H7N7	GenBank	CY095592	
A/Swan/Czech_Republic/5416/2011_H7N7	GenBank	JN966905	JN966907
A/Swan/Slovenia/53/2009_H7N7	GenBank	HQ283357	HQ283359
A/Teal/Finland/9201/2010_H7N3	GenBank	KF183621	
A/Tufted_duck/PT/13771/2006_H7N3	GenBank	HM849003	
A/Turkey/Germany/R11/2001_H7N7	GenBank	CY107856	
A/Turkey/Italy/1010/2003_H7N3	GenBank	CY021365	
A/Turkey/Italy/1067/1999_H7N1	GenBank	CY095506	
A/Turkey/Italy/1083/1999_H7N1	GenBank	KF493003	
A/Turkey/Italy/1086/1999_H7N1	GenBank	KF493012	
A/Turkey/Italy/121964/03_H7N3	GenBank	EU158106	
A/Turkey/Italy/1265/1999_H7N1	GenBank	CY025189	
A/Turkey/Italy/12rs206-1/1999_H7N1	GenBank	KF492991	
A/Turkey/Italy/12rs206-11/1999_H7N1	GenBank	KF493032	
A/Turkey/Italy/12rs206-13/1999_H7N1	GenBank	KF493033	
A/Turkey/Italy/12rs206-14/1999_H7N1	GenBank	KF493037	
A/Turkey/Italy/12rs206-17/1999_H7N1	GenBank	KF493038	
A/Turkey/Italy/12rs206-18/1999_H7N1	GenBank	KF492992	
A/Turkey/Italy/12rs206-20/1999_H7N1	GenBank	KF493040	
A/Turkey/Italy/12rs206-6/1999_H7N1	GenBank	KF493031	
A/Turkey/Italy/1555/1999_H7N1	GenBank	KF493042	
A/Turkey/Italy/1744/1999_H7N1	GenBank	KF493043	
A/Turkey/Italy/2043/2003_H7N3	GenBank	CY022613	
A/Turkey/Italy/214845/2002_H7N3	GenBank	AJ627491	
A/Turkey/Italy/2379/2000_H7N1	GenBank	GU053007	
A/Turkey/Italy/251/2003_H7N3	GenBank	CY020589	
A/Turkey/Italy/2684/2003_H7N3	GenBank	CY095554	
A/Turkey/Italy/2715/1999_H7N1	GenBank	CY025173	
A/Turkey/Italy/2716/1999_H7N1	GenBank	KF493058	

Viral Name	Source	Accession number HA segment	Accession number NA segment
A/Turkey/Italy/2732/1999_H7N1	GenBank	GU052969	
A/Turkey/Italy/2962/2003_H7N3	GenBank	JX515663	
A/Turkey/Italy/2987/2003_H7N3	GenBank	CY021493	
A/Turkey/Italy/3283/1999_H7N1	GenBank	GU052976	
A/Turkey/Italy/3337/2004_H7N3	GenBank	CY021501	
A/Turkey/Italy/3620/2003_H7N3	GenBank	CY021357	
A/Turkey/Italy/3807/2004_H7N3	GenBank	CY020597	
A/Turkey/Italy/3829/2004_H7N3	GenBank	CY028676	
A/Turkey/Italy/3889/99_H7N1	GenBank	AJ493466	
A/Turkey/Italy/4130/2004_H7N3	GenBank	CY029913	
A/Turkey/Italy/4169/1999_H7N1	GenBank	CY006037	
A/Turkey/Italy/4372/2004_H7N3	GenBank	CY095538	
A/Turkey/Italy/4479/2004_H7N3	GenBank	CY020581	
A/Turkey/Italy/4603/99_H7N1	GenBank	AJ493471	
A/Turkey/Italy/4608/2003_H7N3	GenBank	CY021485	
A/Turkey/Italy/68819/03_H7N3	GenBank	EU158100	
A/Turkey/Italy/8000/2002_H7N3	GenBank	CY024738	
A/Turkey/Italy/8458/2002_H7N3	GenBank	CY095562	
A/Turkey/Italy/8534/2002_H7N3	GenBank	CY095530	
A/Turkey/Italy/8912/2002_H7N3	GenBank	CY020605	
A/Turkey/Italy/9739/2002_H7N3	GenBank	CY031611	
A/Turkey/Italy/977/1999_H7N1	GenBank	GU052999	
A/Turkey/Italy12rs206-2/1999_H7N1	GenBank	KF492994	
A/Wild_duck/Mongolia/1-241/2008_H7N9	GenBank	JN029686	
A/Yellow-legged_gull/Republic_of_Georgia/1/2012_mixed	GenBank	CY185372	
A/Northern_shoveler/Mississippi/11OS5900/2011_H8N1	GISAID	EPI512596	
A/American_black_duck/Nova_Scotia/02043/2007_H8N4	GISAID	EPI404484	
A/American_green-winged_teal/California/44287-373/2007_H8N4	GISAID	EPI292438	
A/American_green-winged_teal/Interior_Alaska/9BM5045R0/2009_H8N4	GISAID	EPI433069	EPI433100
A/Chicken/Netherlands/10009401/2010_H8N4	GenBank	KY676328	KY676340
A/Environment/Pennsylvania/NWRC182092-24/2006_H8N4	GISAID	EPI406002	
A/Garganey/Ukraine/05835-NAMRU3/2006_H8N4	GISAID	EPI372512	EPI372511
A/Mallard/Interior_Alaska/8BM1966R1/2008_H8N4	GISAID	EPI299970	
A/Mallard/Interior_Alaska/8MP0547/2008_H8N4	GISAID	EPI299411	
A/Mallard/Interior_Alaska/9BM10537R0/2009_H8N4	GISAID	EPI436880	
A/Mallard/Interior_Alaska/9BM8389R0/2009_H8N4	GISAID	EPI452280	EPI452282
A/Mallard/Minnesota/Sg-00675/2008_H8N4	GISAID	EPI188649	
A/Northern_pintail/Interior_Alaska/8BM2011R1/2008_H8N4	GISAID	EPI299978	
A/Northern_pintail/Interior_Alaska/8BM2046R1/2008_H8N4	GISAID	EPI299419	
A/Northern_pintail/Interior_Alaska/8BM2621R1/2008_H8N4	GISAID	EPI299686	
A/Northern_pintail/Interior_Alaska/8BM2987/2008_H8N4	GISAID	EPI299122	
A/Northern_pintail/Interior_Alaska/8BM3088/2008_H8N4	GISAID	EPI299146	
A/Northern_pintail/Interior_Alaska/8BM3091/2008_H8N4	GISAID	EPI299694	
A/Northern_pintail/Interior_Alaska/8BM3137/2008_H8N4	GISAID	EPI299702	
A/Northern_pintail/Interior_Alaska/8MP0689/2008_H8N4	GISAID	EPI299090	
A/Northern_pintail/Interior_Alaska/9BM11556R0/2009_H8N4	GISAID	EPI452128	
A/Northern_pintail/Interior_Alaska/9BM11643R0/2009_H8N4	GISAID	EPI452142	EPI452144
A/Northern_pintail/Interior_Alaska/9BM6510R0/2009_H8N4	GISAID	EPI452001	EPI452003
A/Northern_pintail/Interior_Alaska/9BM7152R2/2009_H8N4	GISAID	EPI452015	EPI452017
A/Northern_pintail/Interior_Alaska/9BM7240R0/2009_H8N4	GISAID	EPI452050	
A/Northern_pintail/Interior_Alaska/9BM7882R0/2009_H8N4	GISAID	EPI452219	
A/Northern_pintail/Interior_Alaska/9BM8109R0/2009_H8N4	GISAID	EPI436940	
A/Northern_pintail/Interior_Alaska/9BM8237R0/2009_H8N4	GISAID	EPI452226	
A/Northern_pintail/Interior_Alaska/9BM8967R0/2009_H8N4	GISAID	EPI436976	
A/Northern_shoveler/California/AKS273/2007_H8N4	GISAID	EPI178742	
A/Northern_shoveler/California/HKWF1203/2007_H8N4	GISAID	EPI222411	
A/Northern_shoveler/California/HKWF1204/2007_H8N4	GISAID	EPI178782	
A/Northern_shoveler/California/HKWF1325/2007_H8N4	GISAID	EPI160322	
A/Northern_shoveler/Interior_Alaska/9BM2925R0/2009_H8N4	GISAID	EPI432837	
A/Northern_shoveler/Minnesota/Sg-00648/2008_H8N4	GISAID	EPI449391	
A/Mallard/Netherlands/14/2006_H8N4	GenBank	KR862502	
A/Chicken/Netherlands/11004004/2011_H8N4	GenBank	KY676329	KY676339
A/American_black_duck/Illinois/4119/2009_H8N4	GenBank	CY097534	
A/Anas_crecca/Spain/1459/2008_H8N4	GenBank	FN386466	FN386475
A/Common_teal/Netherlands/1/2005_H8N4	GenBank	CY041258	CY041260
A/Duck/Alaska/702/1991_H8N2	GenBank	CY015173	
A/Duck/Hokkaido/207/2014_H8N2	GenBank	LC029898	
A/Duck/Hokkaido/95/1981_H8N4	GenBank	AB450454	AB450455
A/Duck/LA/B174/1986_H8N4	GenBank	GU186458	
A/Duck/Thailand/SP-355/2007_H8N4	GenBank	FJ802406	FJ802407

Viral Name	Source	Accession number HA segment	Accession number NA segment
A/Duck/Tsukuba/255/2005_H8N5	GenBank	AB669137	AB472028
A/Duck/Yangzhou/02/2005_H8N4	GenBank	EF061122	EF061126
A/Mallard_duck/Alberta/7/1987_H8N4	GenBank	CY014583	
A/Mallard/Alaska/708/2005_H8N4	GenBank	CY017749	CY017751
A/Mallard/ALB/194/1992_H8N4	GenBank	CY005972	
A/Mallard/Alberta/283/1977_H8N4	GenBank	CY005970	AY207531
A/Mallard/Interior_Alaska/8BM3061/2008_H8N4	GenBank	CY079099	
A/Mallard/Interior_Alaska/8BM3327/2008_H8N4	GenBank	CY080025	
A/Mallard/Interior_Alaska/8BM3584R1/2008_H8N4	GenBank	CY079677	
A/Mallard/Interior_Alaska/8MP0457R1/2008_H8N4	GenBank	CY079963	
A/Mallard/Minnesota/AI09-1833/2009_H8N4	GenBank	CY140663	CY140665
A/Mallard/Minnesota/AI09-1854/2009_H8N4	GenBank	CY140671	
A/Mallard/Minnesota/AI09-1867/2009_H8N4	GenBank	CY140679	
A/Mallard/Minnesota/Sg-00062/2007_H8N4	GenBank	CY064121	
A/Mallard/Minnesota/Sg-00570/2008_H8N4	GenBank	CY139985	
A/Mallard/Minnesota/Sg-00571/2008_mixed	GenBank	CY139994	
A/Mallard/Minnesota/Sg-00678/2008_H8N4	GenBank	CY042859	
A/Mallard/Minnesota/SG-00680/2008_mixed	GenBank	CY140079	
A/Mallard/Minnesota/SG-00684/2008_H8N4	GenBank	CY140088	
A/Mallard/Minnesota/Sg-00686/2008_H8N4	GenBank	CY140096	
A/Mallard/Minnesota/Sg-00688/2008_H8N4	GenBank	CY042898	
A/Mallard/Minnesota/Sg-00690/2008_H8N4	GenBank	CY042906	
A/Mallard/Minnesota/Sg-00701/2008_H8N4	GenBank	CY140342	
A/Mallard/Netherlands/1/2006_H8N4	GenBank	CY043848	CY043850
A/Mallard/Sweden/101165/2009_H8N4	GenBank	CY183572	CY183574
A/Mallard/Sweden/24/2002_H8N4	GenBank	CY060249	CY064796
A/Mallard/Sweden/2834/2003_H8N4	GenBank	CY183368	CY183370
A/Mallard/Sweden/2990/2003_H8N4	GenBank	CY183441	CY183443
A/Mallard/Sweden/3240/2003_H8N4	GenBank	CY183449	CY183451
A/Mallard/Sweden/3244/2003_H8N4	GenBank	CY183457	CY183459
A/Mallard/Sweden/4486/2004_H8N4	GenBank	CY183465	CY183467
A/Mallard/Sweden/4737/2004_mixed	GenBank	CY183474	
A/Mallard/Sweden/50055/2006_H8N4	GenBank	CY183515	CY183517
A/Mallard/Sweden/51156/2006_H8N4	GenBank	CY183523	CY183525
A/Mallard/Sweden/51671/2006_H8N4	GenBank	CY184600	CY184602
A/Mallard/Sweden/5389/2005_H8N4	GenBank	CY183483	CY183485
A/Mallard/Sweden/541/2002_H8N4	GenBank	CY184592	
A/Mallard/Sweden/58256/2006_H8N4	GenBank	CY183531	CY183533
A/Mallard/Sweden/59475/2007_H8N4	GenBank	CY183539	CY183541
A/Mallard/Sweden/60041/2007_H8N2	GenBank	CY183425	
A/Mallard/Sweden/68537/2007_mixed	GenBank	CY183555	
A/Mallard/Sweden/7242/2004_H8N4	GenBank	CY183491	CY183493
A/Mallard/Sweden/7996/2005_H8N4	GenBank	CY183499	CY183501
A/Mallard/Sweden/8005/2005_H8N4	GenBank	CY183507	CY183509
A/Mallard/Sweden/99377/2009_H8N4	GenBank	CY183564	CY183566
A/Mallard/Wisconsin/11OS4489/2011_H8N4	GenBank	CY166162	
A/Mallard/Wisconsin/2080/1984_H8N4	GenBank	CY178863	
A/Mallard/Wisconsin/2086/1984_H8N4	GenBank	CY178214	CY178216
A/Mallard/Wisconsin/426/1979_H8N4	GenBank	CY180660	CY180662
A/Northern_pintail/Alaska/44204-073/2006_H8N4	GenBank	EU557521	
A/Northern_pintail/Alaska/44340-503/2007_H8N4	GenBank	GU168306	
A/Northern_pintail/Alaska/44420-106/2008_H8	GenBank	GU168307	
A/Northern_pintail/Alaska/44500-066/2009_H8N4	GenBank	JX080768	
A/Northern_shoveler/Netherlands/1/2006_H8N4	GenBank	CY077024	CY077026
A/Pintail_duck/Alberta/114/1979_H8N4	GenBank	CY005971	
A/Pintail/Alaska/246/2005_mixed	GenBank	CY096764	CY096768
A/Pintail/Barrow/140/2005_H8N4	GenBank	EF655844	
A/Pintail/Barrow/38/2005_H8N4	GenBank	EF655828	
A/Ruddy_shelduck/Mongolia/592/2010_H8N6	GenBank	KF501097	
A/Ruddy_shelduck/Mongolia/593/2010_H8N4	GenBank	KF501064	
A/Teal/Chany/444/2009_H8N8	GenBank	CY098524	
A/Turkey/Colorado/235497/2003_H8N4	GenBank	GU051909	
A/Turkey/Ontario/6118/1968_H8N4	GenBank	CY130046	EU429793
A/Teal/Northern_Ireland/14567-10-5257/2007_H9N1	GISAID	EPI383878	
A/Common_coot/Poland/88/13_H9N2	GISAID	EPI505111	
A/Mallard/Iran/C364/2007_H9N2	GISAID	EPI302559	
A/Turkey/England/13437/2013_H9N2	GISAID	EPI585514	
A/Turkey/England/13538/2013_H9N2	GISAID	EPI585519	
A/Turkey/Poland/14/13_H9N2	GISAID	EPI500817	
A/Turkey/Poland/20/13_H9N2	GISAID	EPI505105	

Viral Name	Source	Accession number HA segment	Accession number NA segment
A/Ruddy_turnstone/New_Jersey/AI03-128/2003_H9N7	GISAID	EPI454831	
A/Ruddy_turnstone/New_Jersey/AI03-444/2003_H9N9	GISAID	EPI454810	
A/Mallard/Netherlands/1/2005_H9N2	GenBank	KR862503	
A/Gadwall/Netherlands/2/2006_H9N2	GenBank	KR862504	
A/Chicken/Netherlands/10020245/2010_H9N2	GenBank	KY676330	KY676337
A/Baikal_teal/Xianghai/421/2011_H9N2	GenBank	KC162234	
A/Bewicks_swan/Netherlands/5/2007_H9N2	GenBank	CY041274	
A/Chicken/Korea/25232-96006/1996_H9N2	GenBank	KF188387	KF188388
A/Chicken/Korea/25232-MS96CE6/1996_H9N2	GenBank	KF188345	
A/Chicken/Korea/AI-96004/1996_H9N2	GenBank	GU053194	
A/Chicken/Korea/GH2/2007_H9N2	GenBank	HQ871933	HQ871935
A/Chicken/Korea/MS96-CE6/1996_H9N2	GenBank	GU053186	GU053188
A/Common_murre/Oregon/19497-004/2005_H9N5	GenBank	CY075925	CY075927
A/Duck/Chiba/1/2007_H9N2	GenBank	AB874675	
A/Duck/Germany/113/1995_H9N2	GenBank	HE802066	
A/Duck/Henan/03/2009_H9N2	GenBank	KJ162122	
A/Duck/Hokkaido/13/00_H9N2	GenBank	AB276111	AB276112
A/Duck/Hokkaido/238/2008_H9N2	GenBank	AB485600	
A/Duck/Hokkaido/49/98_H9N2	GenBank	AB125928	
A/Duck/Hokkaido/9/99_H9N2	GenBank	AB262463	AB262465
A/Duck/Hokkaido/HY57/2005_H9N4	GenBank	AB455035	AB455036
A/Duck/Hokkaido/K04/2014_H9N2	GenBank	LC042043	
A/Duck/Hong_Kong/Y439/1997_H9N2	GenBank	KF188265	
A/Duck/Italy/260/2004_H9N8	GenBank	JX273564	
A/Duck/Shantou/1588/00_H9N1	GenBank	AF523389	
A/Duck/Shantou/2030/00_H9N1	GenBank	AF523390	
A/Duck/Thailand/CU-8319T/2010_H9N7	GenBank	KF591855	KF591857
A/Duck/Viet_Nam/340/2001_H9N3	GenBank	EF541420	
A/Duck/Viet_Nam/68/2001_H9N3	GenBank	EF541419	
A/Environment/Bangladesh/1041/2009_H9N2	GenBank	KC757809	
A/Eurasian_wigeon/Netherlands/4/2005_H9N2	GenBank	HM136574	KR862602
A/Gadwall/Netherlands/1/2006_H9N2	GenBank	CY043864	CY043866
A/Knot/England/497/2002_H9N9	GenBank	JX273565	
A/Laughing_gull/Delaware_Bay/5/2003_H9N1	GenBank	CY102720	
A/Mallard/Austria/WV1090234/2007_H9N2	GenBank	GU194485	
A/Mallard/England/7798-6499/2006_H9N2	GenBank	JX273566	
A/Mallard/Finland/10940/2009_H9N2	GenBank	KF183626	
A/Mallard/Finland/13353/2010_H9N2	GenBank	KF183630	
A/Mallard/Finland/13384/2010_H9N2	GenBank	KF183632	
A/Mallard/Finland/13977/2010_H9N2	GenBank	KF183634	
A/Mallard/France/090360/2009_H9N2	GenBank	CY080415	CY080417
A/Mallard/Iran/T366/2007_H9N2	GenBank	FN600117	
A/Mallard/Ireland/PV46B/1993_H9N3	GenBank	AB303077	
A/Mallard/Norway/10_1537/2009_H9N2	GenBank	HE802723	
A/Mallard/Portugal/83660/2009_H9N2	GenBank	CY116614	CY184159
A/Mallard/Portugal/83695/2009_H9N2	GenBank	CY184165	CY184167
A/Mallard/Portugal/99002/2009_H9N2	GenBank	CY116616	
A/Mallard/Portugal/99005/2009_H9N2	GenBank	CY116618	
A/Mallard/Portugal/99006/2009_H9N2	GenBank	CY116620	
A/Mallard/PT/27972-B139/2007_H9N2	GenBank	JF745931	
A/Mallard/Sweden/4932/2004_H9N2	GenBank	CY184117	CY184119
A/Mallard/Sweden/67860/2007_H9N2	GenBank	CY184149	
A/Mallard/Sweden/7146/2004_H9N2	GenBank	CY184125	CY184127
A/Mallard/Sweden/99668/2009_H9N2	GenBank	CY184173	
A/Mallard/Sweden/99785/2009_H9N2	GenBank	CY184181	
A/Mallard/Switzerland/WV1070800/2007_H9N2	GenBank	GU194480	
A/Mallard/Switzerland/WV1070805/2007_H9N2	GenBank	GU194481	
A/Mallard/Switzerland/WV1080875/2008_H9N2	GenBank	GU194482	
A/Mallard/Switzerland/WV3080008/2007_H9N2	GenBank	GU194486	
A/Mallard/Switzerland/WV3080036/2008_H9N2	GenBank	GU194487	
A/Mandarin_duck/Korea/K12-256/2012_H9N2	GenBank	KR234076	
A/Ostrich/South_Africa/AI1586/2008_H9N2	GenBank	GQ404721	
A/Pelican/Zambia/13/2009_H9N1	GenBank	AB569567	
A/Pink-footed_goose/Netherlands/1/2006_H9N2	GenBank	CY041266	CY041268
A/Ruddy_turnstone/Delaware_Bay/261/1999_H9N7	GenBank	CY102532	
A/Ruddy_turnstone/Delaware/AI03-114/2003_H9N2	GenBank	CY144483	
A/Ruddy_turnstone/Delaware/AI03-123/2003_mixed	GenBank	CY144473	
A/Ruddy_turnstone/Delaware/AI03-162/2003_mixed	GenBank	CY144464	CY144466
A/Ruddy_turnstone/Delaware/AI03-163/2003_H9N8	GenBank	CY144547	
A/Ruddy_turnstone/Delaware/AI03-165/2003_H9N5	GenBank	CY144456	CY144458

Viral Name	Source	Accession number HA segment	Accession number NA segment
A/Ruddy_turnstone/Delaware/AI03-180/2003_H9N9	GenBank	CY144563	
A/Ruddy_turnstone/Delaware/AI03-193/2003_H9N5	GenBank	CY144373	CY144375
A/Ruddy_turnstone/Delaware/AI03-200/2003_H9N8	GenBank	CY144555	
A/Ruddy_turnstone/Delaware/AI03-224/2003_H9N9	GenBank	CY144523	
A/Shorebird/DE/261/2003_H9N5	GenBank	CY005992	
A/Shorebird/Delaware_Bay/127/2003_H9N2	GenBank	CY102728	
A/Shorebird/Delaware_Bay/163/2003_H9N2	GenBank	KF188256	
A/Shorebird/Delaware_Bay/246/2003_H9N5	GenBank	CY102736	CY102738
A/Shorebird/Delaware_Bay/276/1999_H9N2	GenBank	KF188373	
A/Shorebird/Delaware_Bay/283/2003_H9N1	GenBank	CY102744	
A/Shorebird/Delaware_Bay/286/2003_H9N2	GenBank	KF188278	
A/Shorebird/Delaware_Bay/293/2003_H9N2	GenBank	KF188333	
A/Shorebird/Delaware_Bay/73/2003_H9N2	GenBank	CY101323	
A/Teal/Finland/10529/2010_H9N2	GenBank	KF183628	
A/Teal/Primorie/3628/02_H9N2	GenBank	DQ787797	
A/Teal/Primorie/3631/02_H9N2	GenBank	DQ787802	
A/Teal/Switzerland/WV1070694/2007_H9N2	GenBank	GU194479	
A/Turkey/Germany/EK224/1995_H9N2	GenBank	JX273569	
A/Turkey/Netherlands/11015452/2011_H9N2	GenBank	JX273570	KY676336
A/Duck/Tsukuba/574/2006_H10N1	GISAID	EPI356629	
A/Shoveler/Egypt/00006-NAMRU3/2007_H10N1	GISAID	EPI372442	
A/Teal/Egypt/12908-NAMRU3/2005_H10N1	GISAID	EPI372481	EPI372480
A/Wild_bird/Korea/A323/2009_H10N1	GISAID	EPI387876	
A/Duck/Hokkaido/W87/2007_H10N2	GISAID	EPI161527	
A/Duck/Hunan/S11205/2012_H10N3	GISAID	EPI461563	
A/Duck/Thailand/LM-CU4747/2009_H10N3	GISAID	EPI314742	
A/Duck/Thailand/LM-CU4753/2009_H10N3	GISAID	EPI314746	
A/Muscovy_duck/Thailand/CU-LM4754/2009_H10N3	GISAID	EPI256770	
A/Duck/Italy/268302/2004_H10N4	GISAID	EPI178493	EPI178495
A/Pied_avocet/Ukraine/05848-NAMRU3/2006_H10N4	GISAID	EPI372496	EPI372495
A/Shoveler/Egypt/01574-NAMRU3/2007_H10N4	GISAID	EPI372458	EPI372457
A/Long-tailed_duck/Wisconsin/10OS3919/2010_H10N6	GISAID	EPI419336	
A/Mallard/Denmark/16109-4/2011-11-14_H10N6	GISAID	EPI541472	
A/Anas_platyrhynchos/Camargue/091863/09_H10N7	GISAID	EPI332944	EPI332955
A/Avian/Israel/201/2001_H10N7	GenBank	JN564724	JN575025
A/Avian/Israel/218/2000_H10N7	GenBank	JN564725	JN575026
A/Avian/Israel/232/2001_H10N7	GISAID	EPI456969	
A/Avian/Israel/297/2001_H10N7	GenBank	JN564728	JN575029
A/Avian/Israel/445/2001_H10N7	GenBank	JN564730	JN575031
A/Avian/Israel/457/2001_H10N7	GenBank	JN564731	JN575032
A/Duck/Italy/62330/2006_H10N7	GISAID	EPI178528	
A/Duck/Italy/73383/2006_H10N7	GISAID	EPI174777	
A/Duck/Shimane/45/1997_H10N7	GenBank	AB296078	AB296079
A/Harbor_seal/Denmark/14-5061-1lu/2014-07_H10N7	GISAID	EPI541474	
A/Mallard/Egypt/EMC-4/2012_H10N7	GISAID	EPI552755	
A/Mallard/Netherlands/1/2012_H10N7	GISAID	EPI552756	
A/Mallard/Netherlands/1/2014_H10N7	GISAID	EPI552751	
A/Mallard/Netherlands/47/2010_H10N7	GenBank	KR862524	KR862722
A/Mallard/Netherlands/50/2010_H10N7	GenBank	KR862527	KR862727
A/Northern_pintail/Egypt/EMC-1/2012_H10N7	GISAID	EPI552754	
A/Seal/Sweden/SVA0546/2014_H10N7	GISAID	EPI545212	
A/Shoveler/Egypt/00600-NAMRU3/2004_H10N7	GISAID	EPI372291	
A/Shoveler/Egypt/01198-NAMRU3/2007_H10N7	GISAID	EPI372402	
A/Shoveler/Egypt/09781-NAMRU3/2004_H10N7	GISAID	EPI372339	EPI372338
A/Teal/Egypt/01207-NAMRU3/2007_H10N7	GISAID	EPI372426	EPI372425
A/Seal/Sweden/SVA0824/2014_/H10N7_H10N7	GISAID	EPI547696	
A/Chicken/77/Jiangxi/2014_H10N8	GISAID	EPI537463	
A/Chicken/Jiangxi/102/2013_H10N8	GISAID	EPI530542	
A/Environment/Dongting_Lake/Hunan/3-9/2007_H10N8	GISAID	EPI221966	
A/Environment/Jiangxi/03366/2013_H10N8	GISAID	EPI530386	
A/Environment/Jiangxi/03367/2013_H10N8	GISAID	EPI530394	
A/Environment/Jiangxi/03413/2013_H10N8	GISAID	EPI530402	
A/Environment/Jiangxi/03489/2013_H10N8	GISAID	EPI530410	
A/Environment/Jiangxi/10615/2014_H10N8	GISAID	EPI530418	
A/Environment/Jiangxi/10721/2014_H10N8	GISAID	EPI530426	
A/Environment/Jiangxi/10738/2014_H10N8	GISAID	EPI530434	
A/Jiangxi-Donghu/346/2013_H10N8	GISAID	EPI497477	
A/Jiangxi/09037/2014_H10N8	GISAID	EPI530450	
A/Mallard/Sweden/7/2003_H10N8	GISAID	EPI251793	
A/Northern_shoveler/Hong_Kong/MPC657/2006_H10N9	GISAID	EPI469806	

Viral Name	Source	Accession number HA segment	Accession number NA segment
A/Northern_shoveler/Hong_Kong/MPE2531/2008_H10N9	GISAID	EPI469805	
A/Northern_shoveler/Hong_Kong/MPE2984/2008_H10N9	GISAID	EPI469808	
A/Shoveler/Egypt/00004-NAMRU3/2007_H10N9	GISAID	EPI372434	
A/Ruddy_turnstone/Netherlands/8/2008_H10N4	GenBank	KR862508	KR862683
A/Mallard/Netherlands/1/2007_H10N7	GenBank	KR862510	
A/Eurasian_wigeon/Netherlands/3/2007_H10N1	GenBank	KR862511	
A/Ruddy_turnstone/Netherlands/2/2009_H10N4	GenBank	KR862515	KR862690
A/Mallard/Lithuania/EMC-1/2011_H10N4	GenBank	KR862380	KR862396
A/Mallard/Lithuania/EMC-1/2010_H10N4	GenBank	KR862381	KR862397
A/Mallard/Netherlands/16/2006_H10N7	GenBank	KR862507	
A/Mallard/Netherlands/2/2008_H10N7	GenBank	KR862517	KR862710
A/Mallard/Netherlands/67/2008_H10N7	GenBank	KR862518	KR862711
A/Mallard/Netherlands/1/2009_H10N7	GenBank	KR862522	KR862715
A/Mallard/Netherlands/53/2008_H10N7	GenBank	KR862523	KR862716
A/Mallard/Netherlands/30/2009_H10	GenBank	KR862525	
A/Mallard/Egypt/EMC-1/2012_H10N7	GenBank	KR862382	
A/Chicken/Netherlands/10008427/2010_H10N7	GenBank	KY676331	KY676345
A/Turkey/Netherlands/09006938/2009_H10N7	GenBank	KY676332	KY676344
A/Mallard/Netherlands/06013952/2006_H10N7	GenBank	KY676349	
A/Mallard/Netherlands/06014516/2006_H10N8	GenBank	KY676350	
A/Avian/Israel/543/2008_H10N7	GenBank	JN564732	JN575033
A/Avian/Israel/824/2005_H10N2	GenBank	JN564733	
A/Chicken/Jiangsu/RD5/2013_H10N9	GenBank	KF006414	
A/Common_teal/Hong_Kong/MPK630/2009_H10N9	GenBank	KF259197	
A/Duck/Guangdong/E1/2012_H10N8	GenBank	JQ924786	
A/Duck/Hunan/S1496/2011_H10N8	GenBank	KP862019	
A/Duck/Hunan/S3137/2009_H10N8	GenBank	KP862027	
A/Duck/Hunan/S4280/2009_H10N8	GenBank	KP862035	
A/Duck/Huzhou/4233/2013_mixed	GenBank	KP413924	
A/Duck/Jiangxi/13875/2005_H10N3	GenBank	KP287830	
A/Duck/Jiangxi/13946/2005_H10N3	GenBank	KP287838	
A/Duck/Jiangxi/15846/2013_H10N3	GenBank	KP285477	
A/Duck/Jiangxi/2039/2005_H10N8	GenBank	KP287926	
A/Duck/Jiangxi/24570/2009_mixed	GenBank	KP287794	
A/Duck/Jiangxi/26141/2009_H10N7	GenBank	KP287966	KP287968
A/Duck/Jiangxi/26182/2009_H10N7	GenBank	KP287974	KP287976
A/Duck/Jiangxi/26281/2009_H10N7	GenBank	KP287982	
A/Duck/Jiangxi/26331/2009_H10N7	GenBank	KP287990	
A/Duck/Jiangxi/27314/2012_H10N7	GenBank	KP287665	KP287667
A/Duck/Jiangxi/302/2006_H10N3	GenBank	KP287846	
A/Duck/Jiangxi/6450/2013_H10N8	GenBank	KP285589	
A/Duck/Jiangxi/6544/2013_H10N8	GenBank	KP285661	
A/Duck/Jiangxi/6556/2013_H10N8	GenBank	KP285677	
A/Duck/Jiangxi/6613/2013_mixed	GenBank	KP287044	
A/Duck/Jiangxi/6648/2013_H10N8	GenBank	KP285733	
A/Duck/Jiangxi/860/2006_H10N3	GenBank	KP287886	
A/Duck/Mongolia/149/03_H10N5	GenBank	AB450456	AB270599
A/Duck/Zhejiang/6D20/2013_H10N2	GenBank	KP063197	
A/Eurasian_wigeon/Netherlands/4/2007_H10N1	GenBank	CY077048	
A/Goose/Guizhou/829/2012_H10N7	GenBank	KF259194	
A/Harbour_seal/Germany/1/2014_H10N7	GenBank	KP137835	KP137832
A/Herring_gull/Netherlands/4/2006_H10N4	GenBank	CY077032	CY077034
A/Mallard/Bavaria/3/2006_H10N7	GenBank	FJ183474	FJ183475
A/Mallard/Korea/1203/2010_H10N8	GenBank	JN817572	
A/Mallard/Korea/1242/2010_H10N6	GenBank	JN817576	
A/Mallard/Netherlands/02/2000_H10N7	GenBank	CY076945	CY076947
A/Mallard/Portugal/79940/2009_H10N7	GenBank	CY116612	
A/Mallard/Republic_of_Georgia/1/2010_H10N1	GenBank	KC190171	KC190180
A/Mallard/Republic_of_Georgia/12/2011_H10N4	GenBank	CY185505	CY185507
A/Mallard/Republic_of_Georgia/14/2011_H10N7	GenBank	CY185689	CY185691
A/Mallard/Republic_of_Georgia/15/2011_H10N7	GenBank	CY185385	CY185387
A/Mallard/Sweden/102087/2009_H10N1	GenBank	CY183839	CY183841
A/Mallard/Sweden/104746/2009_H10N1	GenBank	CY183855	
A/Mallard/Sweden/105186/2009_H10N1	GenBank	CY183863	
A/Mallard/Sweden/105323/2009_H10N1	GenBank	CY183887	CY183889
A/Mallard/Sweden/105402/2009_H10N1	GenBank	CY183911	
A/Mallard/Sweden/105404/2009_H10N1	GenBank	CY183919	
A/Mallard/Sweden/105465/2009_H10N1	GenBank	CY183927	CY183929
A/Mallard/Sweden/105501/2009_H10N1	GenBank	CY183951	CY183953
A/Mallard/Sweden/105522/2009_H10N1	GenBank	JX566079	JX566263

Viral Name	Source	Accession number HA segment	Accession number NA segment
A/Mallard/Sweden/105527/2009_H10N1	GenBank	CY183959	CY183961
A/Mallard/Sweden/105536/2009_H10N1	GenBank	CY183967	
A/Mallard/Sweden/133546/2011_H10N4	GenBank	CY183991	CY183993
A/Mallard/Sweden/1417/2002_H10N7	GenBank	CY183637	CY183639
A/Mallard/Sweden/223/2002_H10N2	GenBank	CY183580	
A/Mallard/Sweden/3151/2003_H10N7	GenBank	CY183645	CY183647
A/Mallard/Sweden/4258/2004_H10N4	GenBank	CY183653	CY183655
A/Mallard/Sweden/4401/2004_H10N4	GenBank	CY183661	CY183663
A/Mallard/Sweden/4411/2004_H10N4	GenBank	CY183669	
A/Mallard/Sweden/51/2002_H10N2	GenBank	HM136575	
A/Mallard/Sweden/51548/2006_H10N4	GenBank	CY183718	CY183720
A/Mallard/Sweden/51619/2006_H10N4	GenBank	CY183734	CY183736
A/Mallard/Sweden/51933/2006_H10N9	GenBank	CY183758	
A/Mallard/Sweden/52903/2006_H10N9	GenBank	CY183783	
A/Mallard/Sweden/5812/2005_H10N9	GenBank	CY183677	
A/Mallard/Sweden/5824/2005_H10N7	GenBank	CY183685	
A/Mallard/Sweden/5932/2005_H10N7	GenBank	CY183401	CY183403
A/Mallard/Sweden/59463/2007_H10N7	GenBank	CY183791	CY183793
A/Mallard/Sweden/60065/2007_mixed	GenBank	CY184665	
A/Mallard/Sweden/6039/2005_H10N4	GenBank	CY184640	CY184642
A/Mallard/Sweden/64476/2007_H10N4	GenBank	CY183799	CY183801
A/Mallard/Sweden/69773/2007_H10N5	GenBank	CY183807	CY183809
A/Mallard/Sweden/69777/2007_H10N6	GenBank	CY183815	
A/Mallard/Sweden/69792/2007_H10N5	GenBank	CY183823	
A/Mallard/Sweden/737/2002_H10N2	GenBank	CY183588	CY183590
A/Mallard/Sweden/766/2002_mixed	GenBank	CY183596	
A/Mallard/Sweden/8004/2005_H10N4	GenBank	CY184657	CY184659
A/Mallard/Sweden/8023/2005_H10N4	GenBank	CY183710	CY183712
A/Mallard/Sweden/93475/2009_H10N6	GenBank	CY183831	
A/Mallard/Sweden/948/2002_H10N9	GenBank	CY183629	
A/Mallard/Switzerland/WV1090023/2009_H10	GenBank	HM179251	
A/Migratory_duck/Jiangxi/10857/2005_H10N2	GenBank	KP287998	KP288000
A/Migratory_duck/Jiangxi/10974/2005_H10N6	GenBank	KP287822	
A/Migratory_duck/Jiangxi/21248/2009_H10N8	GenBank	KP287958	
A/Migratory_duck/Jiangxi/30246/2013_H10N5	GenBank	KP284877	KP284879
A/Migratory_duck/Jiangxi/33038/2013_H10N7	GenBank	KP285893	KP285895
A/Migratory_duck/Jiangxi/33158/2013_H10N7	GenBank	KP285901	
A/Migratory_duck/Jiangxi/33238/2013_H10N7	GenBank	KP285917	
A/Migratory_duck/Jiangxi/593/2005_H10N8	GenBank	KP287934	
A/Migratory_duck/Jiangxi/6847/2003_H10N5	GenBank	KP288014	KP288016
A/Migratory_duck/Jiangxi/9334/2005_H10N6	GenBank	KP287814	
A/Migratory_duck/Jiangxi/9492/2005_H10N6	GenBank	KP288006	
A/Ostrich/SouthAfrica/2001_H10N1	GenBank	GQ247860	
A/Pekin_duck/South_Africa/AI1642/2009_H10N7	GenBank	GQ404728	
A/Ruddy_shelduck/Mongolia/1602/2010_H10N8	GenBank	KF501079	
A/Ruddy_shelduck/Mongolia/974/2010_H10N7	GenBank	KF501093	KF667728
A/Surf_scooter/Mongolia/878V/2009_H10N8	GenBank	KF501083	
A/Velvet_scoter/Mongolia/879V/2009_H10N8	GenBank	KF501096	
A/Wild_bird/Korea/A01/2011_H10N4	GenBank	JN817570	JN817551
A/Wild_bird/Korea/A02/2011_H10N4	GenBank	JN817571	JN817552
A/Wild_bird/Korea/A12/2010_H10N1	GenBank	JN817574	
A/Wild_bird/Korea/A13/2010_H10N1	GenBank	JN817575	
A/Wild_bird/Korea/L110-2/2008_H10N4	GenBank	JN817580	JN817549
A/Mallard/Netherlands/24/2006_H1N1	GenBank		KR862544
A/Mallard/Netherlands/25/2006_H1N1	GenBank		KR862545
A/Mallard/Netherlands/45/2006_H1N1	GenBank		KR862547
A/Mallard/Netherlands/46/2006_H1N1	GenBank		KR862548
A/Lesser_black-backed_gull/Netherlands/1/2007_H11N1	GenBank		KR862553
A/Mallard/Netherlands/24/2009_H6N1	GenBank		KR862555
A/Mallard/Netherlands/12/2005_H1N1	GenBank		KR862535
A/Mallard/Netherlands/14/2005_H1N1	GenBank		KR862536
A/Mallard/Netherlands/15/2005_H1N1	GenBank		KR862537
A/Brent_goose/Netherlands/1/2006_H1N1	GenBank		KR862541
A/Mallard/Netherlands/83/2008_H7N1	GenBank		KR862561
A/Mallard/Netherlands/32/2008_H6N1	GenBank		KR862562
A/Mallard/Netherlands/54/2008_H10N1	GenBank		KR862564
A/Mallard/Netherlands/46/2008_H6N1	GenBank		KR862570
A/Mallard/Netherlands/77/2008_H7N1	GenBank		KR862571
A/Common_teal/Netherlands/1/2008_H6N1	GenBank		KR862574
A/Mallard/Netherlands/28/2009_H1N1	GenBank		KR862580

Viral Name	Source	Accession number HA segment	Accession number NA segment
A/Mallard/Netherlands/39/2011_N1	GenBank		KR862583
A/Mallard/Netherlands/40/2011_H7N1	GenBank		KR862584
A/Mallard/Netherlands/10/2012_H6N1	GenBank		KR862587
A/Mallard/Netherlands/24/2012_N1	GenBank		KR862588
A/Mallard/Netherlands/8/2012_H1N1	GenBank		KR862589
A/Mallard/Netherlands/78/2008_H7N1	GenBank		KR862592
A/Mallard/Netherlands/12/2012_H6N1	GenBank		KR862594
A/Mallard/Netherlands/14/2012_N1	GenBank		KR862599
A/White-fronted_goose/Germany-NI/R482/09_H1N1	GISAID	EPI248525	EPI248524
A/White-fronted_goose/Netherlands/1/1999_H6N1	GenBank		CY060433
A/Domestic_goose/Germany-MV/R3298/2009_H6N1	GISAID		EPI339177
A/Mallard/Italy/4518/2007_H10N1	GISAID		EPI511812
A/Duck/Hokkaido/Vac-1/04_H5N1	GenBank		AB259714
A/Chicken/Hubei/Wf/2002_H5N1	GenBank		DQ997088
A/Duck/Guangxi/xa/2001_H5N1	GenBank		DQ997514
A/Duck/Zhejiang/bj/2002_H5N1	GenBank		DQ997411
A/Chicken/Jilin/hk/2004_H5N1	GenBank		DQ997326
A/Teal/Hong_Kong/2978/03_H5N1	GenBank		EF467813
A/Goose/Guangdong/3/97_H5N1	GenBank		AF364335
A/Duck/France/05066b/2005_H5N1	GenBank		AJ972921
A/Duck/Viet_Nam/Ncvd1/2002_H5N1	GenBank		EF541465
A/Duck/Hokkaido/Vac-3/2007_H5N1	GenBank		AB355931
A/Duck/Eastern_China/01/2002_H3N1	GenBank		EU429718
A/Duck/Eastern_China/341/2003_H3N1	GenBank		EU429719
A/Duck/Eastern_China/213/2003_H3N1	GenBank		EU429723
A/Duck/Eastern_China/233/2003_H3N1	GenBank		EU429733
A/Duck/Eastern_China/262/2003_H3N1	GenBank		EU429734
A/Duck/Eastern_China/103/2003_H1N1	GenBank		EU429749
A/Duck/Eastern_China/152/2003_H1N1	GenBank		EU429751
A/Duck/Eastern_China/243/2003_H3N1	GenBank		EU429752
A/Duck/Eastern_China/252/2003_H3N1	GenBank		EU429753
A/Duck/Eastern_China/253/2003_H3N1	GenBank		EU429754
A/Duck/Eastern_China/267/2003_H3N1	GenBank		EU429755
A/Duck/Eastern_China/231/2003_H3N1	GenBank		EU429766
A/Chicken/Shantou/904/2001_H5N1	GenBank		CY029008
A/Quail/Shantou/3846/2002_H5N1	GenBank		CY029169
A/Chicken/Hong_Kong/715.5/01_H5N1	GenBank		AF509100
A/Duck/Hong_Kong/3461/99_H6N1	GenBank		AJ410564
A/Goose/Hong_Kong/385.3/2000_H5N1	GenBank		AF398421
A/Goose/Hong_Kong/385.5/2000_H5N1	GenBank		AF398422
A/Duck/Hokkaido/143/2003_H7N1	GenBank		AB451873
A/Goose/Hong_Kong/Ww26/2000_H5N1	GenBank		AY059483
A/Goose/Hong_Kong/Ww28/2000_H5N1	GenBank		AY059484
A/Duck/Hong_Kong/Ww381/2000_H5N1	GenBank		AY059485
A/Duck/Hong_Kong/Ww382/2000_H5N1	GenBank		AY059486
A/Duck/Hong_Kong/Ww461/2000_H5N1	GenBank		AY059487
A/Goose/Hong_Kong/Ww491/2000_H5N1	GenBank		AY059489
A/Duck/Hong_Kong/2986.1/2000_H5N1	GenBank		AY059490
A/Goose/Hong_Kong/3014.8/2000_H5N1	GenBank		AY059491
A/Mallard/France/2525/2001_H7N1	GenBank		AM157356
A/Mallard/France/2526/2001_H7N1	GenBank		AM157357
A/Mallard/France/691/2002_H1N1	GenBank		AM157358
A/Swan/Mangystau/3/2006_H5N1	GenBank		FJ436943
A/Chicken/Hong_Kong/317.5/2001b_H5N1	GenBank		AY075028
A/Goose/Hong_Kong/3014.5/2000b_H5N1	GenBank		AY075031
A/Duck/Hong_Kong/380.5/2001_H5N1	GenBank		AY075034
A/Mute_swan/Aktau/1460/2006_H5N1	GenBank		FJ434374
A/Duck/Hokkaido/83/2004_H1N1	GenBank		AB470661
A/Duck/Hokkaido/W73/2007_H1N1	GenBank		AB470663
A/Duck/Mongolia/116/2002_H1N1	GenBank		AB470667
A/Duck/Mongolia/253/2003_H1N1	GenBank		AB470668
A/Duck/Mongolia/540/2001_H1N1	GenBank		AB470669
A/Duck/Mongolia/610/2002_H1N1	GenBank		AB470670
A/Duck/Mongolia/867/2002_H7N1	GenBank		AB473545
A/Teal/Viet_Nam/MBP5/2006_H5N1	GenBank		FJ811998
A/Duck/Tsukuba/67/2005_H1N1	GenBank		AB472015
A/Duck/Chiba/884/2004_H3N1	GenBank		AB472013
A/Anas_plathyrhynchos/Spain/1266/2007_H1N1	GenBank		FN386469
A/Anas_crecca/Spain/1402/2007_H1N1	GenBank		FN386473
A/Duck/Korea/334-15/2008_H6N1	GenBank		GQ414900

Viral Name	Source	Accession number HA segment	Accession number NA segment
A/Duck/Korea/112-25/2008_H6N1	GenBank		GQ414901
A/Spot-billed_duck/Korea/625/2008_H6N1	GenBank		GQ414902
A/Spot-billed_duck/Korea/545/2008_H6N1	GenBank		GQ414903
A/Spot-billed_duck/Korea/527/2008_H6N1	GenBank		GQ414905
A/Spot-billed_duck/Korea/534/2008_H6N1	GenBank		GQ414906
A/Spot-billed_duck/Korea/546/2008_H6N1	GenBank		GQ414907
A/Spot-billed_duck/Korea/536/2008_H6N1	GenBank		GQ414908
A/Spot-billed_duck/Korea/540/2008_H6N1	GenBank		GQ414910
A/Mallard/Korea/L08-8/2008_H6N1	GenBank		GQ414904
A/Mallard/Bavaria/47/2006_N1	GenBank		GU046750
A/Mallard/Bavaria/48/2006_N1	GenBank		GU046751
A/Duck/Bavaria/49/2006_H11N1	GenBank		GU046753
A/Waterfowl/Hong_Kong/378.5/2001_H5N1	GenBank		GU186694
A/Goose/Hong_Kong/437-6/1999_H5N1	GenBank		GU052021
A/Goose/Hong_Kong/437-8/1999_H5N1	GenBank		GU052029
A/Goose/Hong_Kong/485.3/2000_H5N1	GenBank		GU052044
A/Goose/Hong_Kong/1032.6/2000_H5N1	GenBank		GU052052
A/Goose/Hong_Kong/3014.5/2000a_H5N1	GenBank		GU052075
A/Chicken/Hong_Kong/317.5/2001a_H5N1	GenBank		GU052083
A/Hong_Kong/378.1/2001_H5N1	GenBank		GU052091
A/Goose/Hong_Kong/668.1/2001_H5N1	GenBank		GU052467
A/Chicken/Hong_Kong/NT873.3/01-MB_H5N1	GenBank		AY221539
A/Pheasant/Hong_Kong/FY155/01-MB_H5N1	GenBank		AY221543
A/Pheasant/Hong_Kong/FY155/01_H5N1	GenBank		AY221544
A/Chicken/Hong_Kong/FY150/01_H5N1	GenBank		AF509095
A/Mallard/Norway/10_1671/2007_H1N1	GenBank		FN773074
A/Mallard/Jiangxi/6845/2003_H6N1	GenBank		HM144559
A/Aquatic_bird/India/NIV-17095/2007_H11N1	GenBank		CY055177
A/Mallard/PT/23059/2007_H1N1	GenBank		HM849011
A/Chicken/Pakistan/NARC-16945/2010_H3N1	GenBank		HQ165998
A/Mallard/Bavaria/185-26/2008_H1N1	GenBank		HQ259234
A/Mallard/Netherlands/28/2006_H3N1	GenBank		CY076907
A/Duck/Korea/GJ74/2007_H3N1	GenBank		JN087234
A/Duck/Zhejiang/2245/2011_H5N1	GenBank		JN646732
A/Lesser_whistling-duck/Thailand/CU-W3947/2010_H12N1	GenBank		JN982529
A/Lesser_whistling-duck/Thailand/CU-W3946/2010_H12N1	GenBank		JN982521
A/Lesser_whistling-duck/Thailand/CU-W3941/2010_H12N1	GenBank		JN982513
A/Watercock/Thailand/CU-W3699/2009_H12N1	GenBank		JN982505
A/Chicken/England/1415-51184/2010_H9N1	GenBank		JQ609665
A/Mallard/Netherlands/10-Nmkt/1999_N1	GenBank		KC209509
A/Duck/Hokkaido/W26/2012_H12N1	GenBank		AB780370
A/Duck/Fujian/17/2001_H5N1	GenBank		AY585401
A/Duck/Guangdong/01/2001_H5N1	GenBank		AY585403
A/Duck/Guangdong/07/2000_H5N1	GenBank		AY585404
A/Duck/Guangdong/40/2000_H5N1	GenBank		AY585407
A/Duck/Guangxi/35/2001_H5N1	GenBank		AY585410
A/Duck/Shanghai/13/2001_H5N1	GenBank		AY585414
A/Duck/Zhejiang/52/2000_H5N1	GenBank		AY585419
A/Turkey/Italy/604/2000_H7N1	GenBank		KF493262
A/European_teal/Novosibirsk/203/2011_H5N1	GenBank		KF462363
A/European_teal/Novosibirsk/239/2011_H5N1	GenBank		KF462365
A/European_teal/Novosibirsk/261/2011_H5N1	GenBank		KF462367
A/Swine/Fujian/F1/2001_H5N1	GenBank		AY747618
A/Common_goldeneye/Mongolia/1271/2010_H7N1	GenBank		KF667689
A/Mallard/Mongolia/1551/2010_H3N1	GenBank		KF667697
A/Ruddy_shelduck/Mongolia/921C2/2009_H7N1	GenBank		KF667727
A/Swine/Fujian/1/2003_H5N1	GenBank		AY747610
A/Goose/Guangdong/1/96_H5N1	GenBank		AF144304
A/Mallard/Sweden/52405/2006_N1	GenBank		CY164158
A/Duck/Thailand/CU-13011C/2013_N1	GenBank		KJ525983
A/Aquatic_Bird/Hong_Kong/m603/98_H11N1	GenBank		AF098551
A/Mallard/Sweden/4737/2004_N1	GenBank		CY183476
A/Black-headed_gull/Republic_of_Georgia/1/2010_H11N1	GenBank		CY185411
A/Black-headed_gull/Republic_of_Georgia/2/2011_H9N1	GenBank		CY185523
A/Ruddy_turnstone/Iceland/2899/2013_H5N1	GenBank		KM213392
A/Chicken/Taiwan/0824/97_H6N1	GenBank		DQ376693
A/Chicken/Taiwan/na3/98_H6N1	GenBank		DQ376694
A/Chicken/Taiwan/165/99_H6N1	GenBank		DQ376698
A/Duck/Taiwan/WB29/99_H6N1	GenBank		DQ376699
A/Duck/Taiwan/A68/03_H6N1	GenBank		DQ376718

Viral Name	Source	Accession number HA segment	Accession number NA segment
A/Swine/Fujian/2001_H5N1	GenBank		DQ432038
A/Pekin_Duck/France/M-2060/01_H1N1	GenBank		AJ697876
A/Environment/Hong_Kong/437-4/99_H5N1	GenBank		AF216714
A/Environment/Hong_Kong/437-10/99_H5N1	GenBank		AF216738
A/Mallard/Netherlands/85/2006_N2	GenBank		KR862609
A/Mallard/Netherlands/12/2006_H5N2	GenBank		KR862611
A/Mallard/Netherlands/43/2006_H5N2	GenBank		KR862612
A/Mallard/Netherlands/58/2006_H4N2	GenBank		KR862614
A/Mallard/Netherlands/63/2006_H5N2	GenBank		KR862615
A/Bewicks_swan/Netherlands/4/2006_H9N2	GenBank		KR862616
A/Mallard/Netherlands/85/2008_N2	GenBank		KR862617
A/White-fronted_goose/Netherlands/8/2009_H6N2	GenBank		KR862635
A/Bewicks_swan/Netherlands/1/2010_H5N2	GenBank		KR862637
A/White-fronted_goose/Netherlands/6/2010_H6N2	GenBank		KR862639
A/White-fronted_goose/Netherlands/7/2011_N2	GenBank		KR862642
A/Mallard/Netherlands/13/2005_H6N2	GenBank		KR862600
A/Eurasian_wigeon/Netherlands/2/2005_H1N2	GenBank		KR862601
A/Eurasian_wigeon/Netherlands/5/2005_N2	GenBank		KR862603
A/Mallard/Netherlands/19/2005_H3N2	GenBank		KR862605
A/Mallard/Netherlands/19/2007_H6N2	GenBank		KR862643
A/Mallard/Netherlands/3/2008_H6N2	GenBank		KR862644
A/Mallard/Netherlands/5/2009_H5N2	GenBank		KR862645
A/Mallard/Netherlands/43/2008_H3N2	GenBank		KR862646
A/Mallard/Netherlands/44/2008_H3N2	GenBank		KR862647
A/Mallard/Netherlands/45/2008_H4N2	GenBank		KR862648
A/Bewicks_swan/Netherlands/5/2008_H5N2	GenBank		KR862651
A/White-fronted_goose/Netherlands/5/2008_H5N2	GenBank		KR862652
A/White-fronted_goose/Netherlands/4/2009_H5N2	GenBank		KR862653
A/Mallard/Netherlands/18/2009_H6N2	GenBank		KR862654
A/Eurasian_wigeon/Netherlands/1/2009_H5N2	GenBank		KR862655
A/Black-headed_gull/Netherlands/8/2010_H13N2	GenBank		KR862656
A/Mallard/Netherlands/28/2010_H5N2	GenBank		KR862657
A/Mallard/Netherlands/34/2010_H3N2	GenBank		KR862658
A/Mallard/Netherlands/13/2011_H3N2	GenBank		KR862659
A/Eurasian_wigeon/Netherlands/1/2011_N2	GenBank		KR862660
A/Mallard/Netherlands/17/2012_H3N2	GenBank		KR862663
A/Mallard/Netherlands/27/2012_N2	GenBank		KR862665
A/Mallard/Netherlands/15/2012_H3N2	GenBank		KR862664
A/Mallard/Netherlands/27/2008_H3N2	GenBank		KR862667
A/Mallard/Netherlands/50/2008_H3N2	GenBank		KR862668
A/Mallard/Netherlands/52/2008_H5N2	GenBank		KR862674
A/White-fronted_goose/Netherlands/6/2008_H5N2	GenBank		KR862675
A/Eurasian_wigeon/Netherlands/1/2010_H5N2	GenBank		KR862676
A/Mallard/Netherlands/55/2010_H3N2	GenBank		KR862677
A/Mallard/Netherlands/18/2012_H4N2	GenBank		KR862680
A/Wild_duck/Germany/WV2555/2006_H3N2	GISAID		EPI185342
A/Pheasant/Ireland/PV12-010728/12_H5N2	GISAID		EPI375596
A/Chicken/Italy/11VIR-7548/2011_H5N2	GISAID		EPI464929
A/Turkey/Italy/12VIR-6607-5/2012_H5N2	GISAID		EPI464937
A/Chicken/Italy/12VIR-7785-67/2012_H5N2	GISAID		EPI464945
A/Turkey/Italy/12VIR-8036-2/2012_H5N2	GISAID		EPI464953
A/Mule_duck/Bulgaria/61/2010_mixed	GISAID		EPI574173
A/Mule_duck/Bulgaria/64/2010_mixed	GISAID		EPI574211
A/Mule_duck/Bulgaria/369/2009_H4N2	GISAID		EPI574213
A/Mule_duck/Bulgaria/105/2008_mixed	GISAID		EPI574258
A/Mule_duck/Bulgaria/174/2009_H6N2	GISAID		EPI574265
A/Mule_duck/Bulgaria/596/2010_H4N2	GISAID		EPI574276
A/Duck/Hong_Kong/301/1978_H7N2	GenBank		AB302790
A/Korea/KBNP-0028/2000_H9N2	GenBank		EF620902
A/Garganey/SanJiang/160/2006_H5N2	GenBank		EF634334
A/Duck/Jiang_Xi/1286/2005_H5N2	GenBank		EF597303
A/Duck/Jiang_Xi/3345/2005_H5N2	GenBank		EF597310
A/Duck/Eastern_China/164/2002_H6N2	GenBank		EU429762
A/Swine/Korea/C13/2008_H5N2	GenBank		FJ461597
A/Duck/Primorie/2621/2001_H5N2	GenBank		GQ162788
A/Spotbill_duck/Xuyi/18/2005_H5N2	GenBank		GQ184331
A/Spotbill_duck/Xuyi/6/2005_H11N2	GenBank		GQ184332
A/Mallard/Xuyi/10/2005_H5N2	GenBank		GQ184334
A/Duck/Tsukuba/9/2005_H2N2	GenBank		AB472017
A/Mallard/Netherlands/2/2005_H4N2	GenBank		CY041252

Viral Name	Source	Accession number HA segment	Accession number NA segment
A/Mallard/Netherlands/26/2005_H11N2	GenBank		CY041420
A/Duck/Shimane/19/2006_H5N2	GenBank		AB472053
A/Duck/Niigata/477/2007_H5N2	GenBank		AB472055
A/Wild_bird/Korea/A81/2009_H5N2	GenBank		GU086246
A/Duck/Korea/A14/2008_H5N2	GenBank		GU086248
A/Duck/Korea/A93/2008_H5N2	GenBank		GU086249
A/Gadwall/Altai/1202/2007_H5N2	GenBank		CY049758
A/Mallard/Netherlands/3/1999_H5N2	GenBank		GU052558
A/Mallard/Sweden/7/2002_H5N2	GenBank		GU052566
A/Chicken/France/03426/2003_H5N2	GenBank		CY046126
A/Duck/France/080032/2008_H5N2	GenBank		CY046176
A/Aquatic_bird/Korea/W96/2005_H5N2	GenBank		GU361236
A/Aquatic_bird/Korea/W113/2006_H5N2	GenBank		GU361237
A/Aquatic_bird/Korea/W114/2006_H5N2	GenBank		GU361238
A/Aquatic_bird/Korea/W121/2006_H5N2	GenBank		GU361240
A/Aquatic_bird/Korea/W125/2006_H5N2	GenBank		GU361241
A/Aquatic_bird/Korea/W163/2007_H5N2	GenBank		GU361244
A/Aquatic_bird/Korea/W216/2007_H5N2	GenBank		GU361256
A/Aquatic_bird/Korea/W230/2007_H5N2	GenBank		GU361262
A/Aquatic_bird/Korea/W234/2007_H5N2	GenBank		GU361264
A/Aquatic_bird/Korea/W344/2008_H5N2	GenBank		GU361267
A/Mallard/Sweden/4/2002_H10N2	GenBank		CY060302
A/Teal/Norway/10_1360/2007_H4N2	GenBank		FN773069
A/Duck/Fujian/11339/2005_H6N2	GenBank		HM144700
A/Duck/Fujian/8719/2005_H6N2	GenBank		HM144699
A/Duck/Fujian/7033/2005_H6N2	GenBank		HM144698
A/Duck/Fujian/5643/2005_H6N2	GenBank		HM144697
A/Duck/Fujian/5426/2005_H6N2	GenBank		HM144696
A/Duck/Fujian/5117/2005_H6N2	GenBank		HM144695
A/Duck/Fujian/4125/2005_H6N2	GenBank		HM144694
A/Duck/Fujian/3937/2005_H6N2	GenBank		HM144693
A/Duck/Fujian/3701/2005_H6N2	GenBank		HM144692
A/Duck/Fujian/3193/2005_H6N2	GenBank		HM144691
A/Duck/Fujian/1695/2005_H6N2	GenBank		HM144689
A/Duck/Fujian/629/2005_H6N2	GenBank		HM144688
A/Duck/Fujian/420/2005_H6N2	GenBank		HM144687
A/Duck/Shantou/22596/2005_H6N2	GenBank		HM144679
A/Mallard/Shantou/198/2005_H6N2	GenBank		HM144661
A/Wild_duck/Shantou/180/2005_H6N2	GenBank		HM144660
A/Wild_duck/Shantou/7900/2004_H6N2	GenBank		HM144658
A/Wild_duck/Shantou/7307/2004_H6N2	GenBank		HM144656
A/Wild_duck/Shantou/3433/2003_H6N2	GenBank		HM144645
A/Wild_duck/Shantou/2853/2003_H6N2	GenBank		HM144642
A/Mallard/Netherlands/7/2007_H4N2	GenBank		CY076923
A/Mallard/Sweden/74/2003_H5N2	GenBank		CY076931
A/Herring_gull/Atyrau/2186/2007_H11N2	GenBank		HQ541743
A/Swine/KU/16/2001_H7N2	GenBank		CY067688
A/Duck/France/05057a/2005_H6N2	GenBank		AM489442
A/Spur-winged_goose/Nigeria/226/2008_H5N2	GenBank		FR771826
A/Spur-winged_goose/Nigeria/210/2008_H5N2	GenBank		FR771827
A/Spur-winged_goose/Nigeria/2/2008_H5N2	GenBank		FR771828
A/Avian/Japan/8KI0148/2008_H4N2	GenBank		CY088723
A/Ostrich/South_Africa/9508103/95_H9N2	GenBank		AF508575
A/Chicken/Korea/99029/99_H9N2	GenBank		AF508582
A/Chicken/Eastern_China/43/2007_H6N2	GenBank		JF965302
A/Teal/Norway/10_1037/2010_H3N2	GenBank		FR873768
A/Mallard/Norway/10_1368/2010_H6N2	GenBank		FR873773
A/Avian/Israel/289/2001_H6N2	GenBank		JN575028
A/Chicken/Korea/KNUSWR09/2009_H9N2	GenBank		JN852797
A/Duck/Jiangxi/5748/2006_H6N2	GenBank		CY109332
A/Mallard/Netherlands/14/2007_H2N2	GenBank		CY121977
A/Mallard/Sweden/58112/2006_H2N2	GenBank		CY121929
A/Mallard/Sweden/58451/2006_H2N2	GenBank		CY121937
A/Mallard/Sweden/68735/2007_H2N2	GenBank		CY121945
A/White-fronted_goose/Netherlands/22/1999_H2N2	GenBank		CY121961
A/Mallard/Sweden/99858/2009_H6N2	GenBank		JX566198
A/Mallard/Sweden/99843/2009_H6N2	GenBank		JX566185
A/Mallard/Sweden/99820/2009_H11N2	GenBank		JX566177
A/Mallard/Sweden/100537/2009_H6N2	GenBank		JX566222
A/Duck/Japan/9UO036/2009_H5N2	GenBank		JX673923

Viral Name	Source	Accession number HA segment	Accession number NA segment
A/Duck/Japan/9UO139/2009_H5N2	GenBank		JX673929
A/Duck/Japan/9UO025/2009_H5N2	GenBank		JX673936
A/Duck/Jiangsu/26/2004_H3N2	GenBank		KC261671
A/Mallard/Finland/13748/2007_H5N2	GenBank		KF183617
A/Mallard/Sweden/21/2002_H5N2	GenBank		KF695272
A/Chicken/New_Jersey/251-4/2008_H5N2	GenBank		KJ018202
A/Mallard/Sweden/274/2002_H4N2	GenBank		CY164218
A/Mallard/Sweden/906/2002_H4N2	GenBank		CY164258
A/Mallard/Sweden/1195/2002_H4N2	GenBank		CY164274
A/Mallard/Sweden/58359/2006_mixed	GenBank		CY165021
A/Mallard/Sweden/58463/2006_mixed	GenBank		CY165049
A/Mallard/Sweden/68504/2007_H4N2	GenBank		CY165100
A/Mallard/Netherlands/1/2007_H3N2	GenBank		CY043818
A/Eurasian_wigeon/Netherlands/3/2005_H9N2	GenBank		CY043858
A/Mallard/Sweden/58705/2006_H5N2	GenBank		CY184143
A/Mallard/Sweden/79196/2008_mixed	GenBank		CY184415
A/Migratory_duck/Jiangxi/10861/2005_H10N2	GenBank		KP287808
A/Duck/Nanchang/1749/1992_H11N2	GenBank		CY005532
A/Chicken/Korea/MS96/96_H9N2	GenBank		AF203786
A/Muskrat/Russia/63/2014_H2N2	GenBank		KR052706
A/Duck/Denmark/65047/04_H5N2	GenBank		DQ251448
A/Dk/Hong_Kong/293/1978_H7N2	GenBank		CY005620
A/Duck/Kingmen/E322/04_H6N2	GenBank		DQ376721
A/Ruddy_turnstone/Netherlands/1/2008_H10N4	GenBank		KR862686
A/Ruddy_turnstone/Netherlands/5/2008_H10N4	GenBank		KR862688
A/Ruddy_turnstone/Netherlands/1/2009_H10N4	GenBank		KR862689
A/Mallard/Netherlands/11/2006_H8N4	GenBank		KR862681
A/Mallard/Netherlands/13/2006_H8N4	GenBank		KR862682
A/Mallard/Netherlands/30/2011_H6N4	GenBank		KR862693
A/Ruddy_Turnstone/Delaware/67/98_H12N4	GISAID		EPI16616
A/Pintail/Alaska/314/2005_H12N4	GISAID		EPI307548
A/Blue-winged_teal/Guatemala/CIP049-04/2010_H8N4	GenBank		CY096650
A/Red_knot/Delaware_Bay/227/1994_mixed	GISAID		EPI345275
A/Shorebird/Delaware_Bay/215/1994_mixed	GISAID		EPI437174
A/Ruddy_turnstone/Delaware_Bay/124/1994_mixed	GISAID		EPI437442
A/Ruddy_turnstone/Delaware_Bay/150/1994_H1N4	GISAID		EPI437470
A/Mallard/Sweden/100546/2009_H8N4	GenBank		JX566224
A/Blue-winged_teal/ALB/685/1982_H6N4	GISAID		EPI85929
A/Mallard_duck/Alberta/299/1977_H4N4	GISAID		EPI87231
A/Mallard/Alberta/194/1992_H8N4	GISAID		EPI87925
A/Duck/Hokkaido/18/00_H10N4	GenBank		AB274042
A/Chicken/New_South_Wales/2/1997_H7N4	GenBank		CY022695
A/Chicken/New_South_Wales/327/1997_H7N4	GenBank		CY022703
A/Emu/New_South_Wales/775/1997_H7N4	GenBank		CY022711
A/Duck/Eastern_China/01/2005_H8N4	GenBank		EU429780
A/Mallard/Iran/V16/04_H8N4	GenBank		AM933239
A/Duck/Hubei/137/1985_H10N4	GenBank		EU559265
A/Mink/Sweden/3900/1984_H10N4	GenBank		GQ176142
A/Mallard/Gloucestershire/PD374/1985_H10N4	GenBank		GQ176126
A/Fowl/Hampshire/PD378/1985_H10N4	GenBank		GQ176118
A/Whistling_swan/Shimane/468/1988_H10N4	GenBank		GQ176110
A/Anas_plathyrhynchos/Spain/1495/2008_H10N4	GenBank		FN386476
A/Anas_plathyrhynchos/Spain/1502/2008_H8N4	GenBank		FN386477
A/Gray_teal/Western_Australia/1840/1979_H4N4	GenBank		CY045265
A/Chicken/NSW/1651/1997_H7N4	GenBank		GU053096
A/Mallard/Sweden/8/2003_H8N4	GenBank		CY060406
A/Mallard/PT/9408-3/2006_H9N4	GenBank		HM849002
A/Duck/Victoria/24/1981_H8N4	GenBank		CY094945
A/Teal/Chany/7119/2008_H15N4	GenBank		CY098542
A/Duck/Tsukuba/20/2007_H8N4	GenBank		AB669141
A/Duck/Thailand/CU-9754C/2010_H7N4	GenBank		JX307164
A/Duck/Thailand/CU-10524C/2011_H7N4	GenBank		JX307193
A/Duck/Thailand/CU-9744C_/2010_H7N4	GenBank		JX307210
A/Environment/Korea/PSC13-43/2008_H8N4	GenBank		JX679162
A/Mallard/Alberta/58/1989_H6N4	GenBank		CY126443
A/Ruddy_turnstone/Delaware/AI03-378/2003_H12N4	GenBank		CY144383
A/Duck/Thailand/CU-10510C/2011_H7N4	GenBank		KF591867
A/Mallard/Sweden/17/2002_H10N4	GenBank		KF695363
A/Mallard/Wisconsin/772/1982_H6N4	GenBank		CY178136
A/Duck/Wisconsin/2366/1985_N4	GenBank		CY177458

Viral Name	Source	Accession number HA segment	Accession number NA segment
A/Mallard/Wisconsin/1360/1983_H7N4	GenBank		CY179525
A/Mallard/Sweden/396/2002_H10N4	GenBank		CY184618
A/Mallard_duck/ALB/7/1987_H8N4	GenBank		CY004998
A/Mallard_duck/ALB/581/1983_H4N4	GenBank		CY004813
A/Mallard/Netherlands/83/2008_H12N5	GenBank		KR862695
A/Eurasian_wigeon/Netherlands/2/2009_H12N5	GenBank		KR862696
A/Mallard/Netherlands/4/2011_H12N5	GenBank		KR862698
A/Mallard/Netherlands/13/2008_H4N5	GenBank		KR862699
A/Duck/Hokkaido/24/04_H10N5	GISAID		EPI160652
A/Mallard/Denmark/77-64590-5/2005_H7N5	GISAID		EPI174859
A/Mallard/California/6524/2008_H12N5	GISAID		EPI328292
A/Mallard/Alberta/220/2006_	GISAID		EPI343416
A/Mallard/Alberta/12/1993_	GISAID		EPI344668
A/Arenaria_interpres/Belgium/02936pcs1/2010_H12N5	GISAID		EPI345387
A/Mallard/Ohio/170/1999_H6N5	GISAID		EPI44086
A/Emperor_goose/Alaska/44064-075/2006_H2N5	GISAID		EPI442443
A/Mallard/Minnesota/182729/1998_H6N5	GISAID		EPI448392
A/Green-winged_teal/Minnesota/Sg-00820/2008_H4N5	GISAID		EPI449492
A/Ruddy_turnstone/New_Jersey/AI07-697/2007_H12N5	GISAID		EPI454987
A/Ruddy_turnstone/New_Jersey/AI07-803/2007_H12N5	GISAID		EPI455001
A/Mule_duck/Bulgaria/674/2010_H6N5	GISAID		EPI574266
A/Mallard/Alberta/202/1996_H2N5	GISAID		EPI85385
A/Pintail/Alberta/49/2003_H9N5	GISAID		EPI86317
A/Mallard/Alberta/52/1997_H12N5	GISAID		EPI86417
A/Green-winged_teal/ALB/199/1991_H12N5	GISAID		EPI88775
A/Duck/Hokkaido/66/01_H12N5	GenBank		AB270747
A/Duck/Hokkaido/1058/2001_H4N5	GenBank		AB270594
A/Chicken/Hubei/119/1983_H10N5	GenBank		EU559271
A/Duck/Tsukuba/11/2004_H6N5	GenBank		AB472026
A/Duck/Shiga/69/2006_H6N5	GenBank		AB472027
A/Anas_plathyrhynchos/Spain/1252/2007_H6N4	GenBank		FN386470
A/Mallard/Switzerland/WV4060167/2006_H3N5	GenBank		GQ415323
A/Duck/Yangzhou/013/2008_H6N5	GenBank		GU220601
A/Duck/Eastern_China/031/2009_H5N5	GenBank		GU727663
A/Duck/Eastern_China/008/2008_H5N5	GenBank		GU727655
A/Mallard/Netherlands/2/1999_H3N5	GenBank		CY060263
A/Black-headed_gull/Netherlands/1/2006_H4N5	GenBank		CY076994
A/Avian/Japan/8KI0040/2008_H3N5	GenBank		CY079277
A/Duck/Vietnam/G18/2009_H12N5	GenBank		AB593481
A/Duck/Mongolia/OIE-7457/2011_H3N5	GenBank		AB701297
A/Goose/Guangdong/K0103/2010_H5N5	GenBank		JQ973688
A/Quail/Jiangsu/K0104/2010_H5N5	GenBank		JQ973680
A/Mallard/Sweden/30/2002_H2N5	GenBank		CY122069
A/Duck/Guangxi/GXd-1/2009_H6N5	GenBank		JX293561
A/Aquatic_bird/Korea/CN5/2009_H6N5	GenBank		JX465642
A/Swine/Hubei/10/2008_H10N5	GenBank		JX500445
A/Mallard/Sweden/100127/2009_H12N5	GenBank		JX566221
A/Wild_duck/Korea/SH12-7/2008_H10N5	GenBank		JX679163
A/Duck/HuBei/03/2010_H5N5	GenBank		JX878685
A/Ruddy_turnstone/Delaware_Bay/118/2007_mixed	GenBank		CY127777
A/Duck/Vietnam/OIE-707/2011_H11N5	GenBank		AB781683
A/Mallard/Astrakhan/263/1982_H14N5	GenBank		AB289336
A/Ruddy_turnstone/New_Jersey/AI07-796/2007_H12N5	GenBank		CY144725
A/Mallard/Finland/10952/2008_H4N5	GenBank		KF183615
A/Black-headed_gull/Iceland/1298/2011_H10N5	GenBank		CY149486
A/Duck/Guangdong/Wy11/2008_H5N5	GenBank		CY091629
A/Duck/Guangdong/Wy19/2008_H5N5	GenBank		CY091637
A/Duck/Guangdong/Wy24/2008_H5N5	GenBank		CY091645
A/Mallard/Sweden/50709/2006_H4N5	GenBank		CY164570
A/Mallard/Sweden/343/2002_H12N5	GenBank		CY184001
A/Mallard/Sweden/2213/2003_H12N5	GenBank		CY184025
A/Mallard/Sweden/3328/2003_H12N5	GenBank		CY184033
A/Mallard/Sweden/50968/2006_H12N5	GenBank		CY184041
A/Mallard/Sweden/60069/2007_H12N5	GenBank		CY184049
A/Mallard/Sweden/68529/2007_H12N5	GenBank		CY184057
A/Black-headed_gull/Republic_of_Georgia/9/2012_H2N5	GenBank		CY185619
A/Mallard/Sweden/79389/2008_mixed	GenBank		CY186278
A/Mallard/Sweden/80329/2008_H11N5	GenBank		CY186398
A/Mallard/Alberta/26/2001_mixed	GenBank		CY185798
A/Migratory_duck/Jiangxi/31454/2013_H10N5	GenBank		KP284895

Viral Name	Source	Accession number HA segment	Accession number NA segment
A/Migratory_duck/Jiangxi/31577/2013_H10N5	GenBank		KP285887
A/Migratory_duck/Jiangxi/7231/2003_H10N5	GenBank		KP288024
A/Black_duck/AUS/4045/1980_H6N5	GenBank		CY005693
A/Mallard/Netherlands/2/2007_H10N7	GenBank		KR862705
A/Mallard/Netherlands/3/2007_H10N7	GenBank		KR862706
A/Northern_shoveler/Egypt/EMC-4/2012_H10N7	GenBank		KR862401
A/Mallard/Netherlands/68/2008_H10N7	GenBank		KR862712
A/Mallard/Netherlands/3/2009_H7N7	GenBank		KR862718
A/Mallard/Netherlands/7/2009_H7N7	GenBank		KR862721
A/Mallard/Netherlands/21/2010_H10N7	GenBank		KR862723
A/Mallard/Netherlands/22/2010_H10N7	GenBank		KR862724
A/Mallard/Netherlands/23/2010_H10N7	GenBank		KR862725
A/Northern_shoveler/Egypt/EMC-2/2012_H10N7	GenBank		KR862402
A/Mallard/Netherlands/73/2008_H10N7	GenBank		KR862733
A/Mallard/Netherlands/74/2008_H10N7	GenBank		KR862734
A/Mallard/Netherlands/15/2013_H7N7	GenBank		KR862742
A/Mallard/Poland/16/09_H7N7	GISAID		EPI254380
A/Turkey/Netherlands/03003568/03_H7N7	GISAID		EPI290239
A/Chicken/Germany/R1801/2011_H7N7	GISAID		EPI356304
A/Turkey/Germany/R1775/2011_H7N7	GISAID		EPI356305
A/Turkey/Germany-NI/R534/2013_H7N7	GISAID		EPI470367
A/Duck/Mongolia/583/02_H4N7	GenBank		AB289334
A/Anser_anser/Germany/R752/06_H7N7	GenBank		AM933236
A/Anas_crecca/Germany/Wv177/05_H7N7	GenBank		AM933237
A/Whooper_swan/Norway/10_438/2006_H7N7	GenBank		FM179762
A/Duck/Hokkaido/W90/2007_H10N7	GenBank		AB450445
A/Duck/Taiwan/4201/99_H7N7	GenBank		AB450450
A/Mallard/Korea/GH170/2007_H7N7	GenBank		FJ750866
A/Magpie/Korea/YJD174/2007_H7N7	GenBank		FJ750856
A/Mallard/Sweden/S90735/2003_H7N7	GenBank		FJ803183
A/Mallard/Korea/GH171/2007_H7N7	GenBank		FJ959088
A/Duck/Shimane/18/2006_H7N7	GenBank		AB472030
A/Duck/Shiga/B149/2007_H7N7	GenBank		AB472031
A/Duck/Tsukuba/664/2007_H7N7	GenBank		AB472059
A/Duck/Tsukuba/922/2008_H7N7	GenBank		AB472060
A/Duck/Chiba/13/2008_H7N7	GenBank		AB472061
A/Duck/Tsukuba/30/2007_H7N7	GenBank		AB472063
A/Northern_pintail/Aomori/372/2008_H7N7	GenBank		AB516423
A/Northern_pintail/Aomori/1001/2008_H7N7	GenBank		AB517633
A/Northern_pintail/Akita/1366/2008_H7N7	GenBank		AB517634
A/Northern_pintail/Akita/1367/2008_H7N7	GenBank		AB517636
A/Mallard/PT/14683/2006_H6N7	GenBank		HM849014
A/Chicken/Netherlands/1/2003_H7N7	GenBank		AY340077
A/Netherlands/219/03_H7N7	GenBank		AY340079
A/King_eider/Alaska/44068-067/2006_H4N7	GenBank		JX081153
A/Mallard/Sweden/109/2002_H2N7	GenBank		CY122033
A/Mallard/Sweden/112/2002_H2N7	GenBank		CY122049
A/Mallard/Sweden/9/2003_H2N7	GenBank		CY122141
A/Chicken/Germany/R28/03_H7N7	GenBank		AJ620349
A/Mallard/Portugal/79905/2009_H10N7	GenBank		CY116607
A/Mallard/Korea/GG2/2007_H7N7	GenBank		KC609788
A/Wild_bird_feces/Korea/HDR22/2006_H7N7	GenBank		KC609785
A/Wild_duck/Korea/MHC40-28/2010_H7N7	GenBank		KC609802
A/Mallard/Korea/NHG187/2008_H7N7	GenBank		KC609794
A/Wild_bird_feces/Korea/HDR23/2006_H7N7	GenBank		KC609786
A/Mallard/64650/03_H5N7	GenBank		AY531030
A/Wild_goose/Dongting/PC0360/2012_H7N7	GenBank		KC876685
A/Duck/Fukui/1/2004_H7N7	GenBank		AB824727
A/Turkey/Ireland/PV8/1995_H7N7	GenBank		KF160885
A/Environment/Hunan/S4484/2011_H12N7	GenBank		CY146766
A/Duck/Fujian/5408/2008_H7N7	GenBank		KF259621
A/Duck/Fujian/5476/2008_H7N7	GenBank		KF259622
A/Duck/Jiangxi/16309/2010_H7N7	GenBank		KF259628
A/Duck/Jiangxi/16326/2010_H7N7	GenBank		KF259629
A/Duck/Jiangxi/16769/2010_H7N7	GenBank		KF259630
A/Duck/Jiangxi/21980/2010_H7N7	GenBank		KF259631
A/Duck/Jiangxi/1717/2003_H7N7	GenBank		KF259633
A/Duck/Jiangxi/1748/2003_H7N7	GenBank		KF259634
A/Common_teal/Hong_Kong/MPM1670/2011_H7N7	GenBank		KF259636
A/Common_teal/Hong_Kong/MPL634/2011_H7N7	GenBank		KF259637

Viral Name	Source	Accession number HA segment	Accession number NA segment
A/Common_teal/Hong_Kong/MPM1740/2011_H7N7	GenBank		KF259638
A/Wild_waterfowl/Hong_Kong/MPL705/2011_H7N7	GenBank		KF259639
A/Wild_waterfowl/Hong_Kong/MPM2121/2011_H7N7	GenBank		KF259640
A/Wild_waterfowl/Hong_Kong/MPL1006/2011_H7N7	GenBank		KF259641
A/Mallard/Sweden/105/2002_H7N7	GenBank		KF695338
A/Mallard/Sweden/7206/2004_H7N7	GenBank		CY183419
A/Mallard/Sweden/124987/2010_H7N7	GenBank		CY183435
A/Mallard/Sweden/6148/2005_H10N7	GenBank		CY183704
A/Mallard/Sweden/885/2002_H7N7	GenBank		CY184506
A/Mallard/Sweden/1337/2002_H7N7	GenBank		CY184514
A/Mallard/Sweden/1645/2002_H7N7	GenBank		CY184522
A/Mallard/Sweden/5944/2005_H7N7	GenBank		CY184586
A/Mallard/Republic_of_Georgia/1/2010_H10N7	GenBank		CY185419
A/Domestic_duck/Republic_of_Georgia/1/2010_H10N7	GenBank		CY185451
A/Domestic_duck/Republic_of_Georgia/2/2010_H10N7	GenBank		CY185459
A/Black-headed_gull/Republic_of_Georgia/7/2012_H2N7	GenBank		CY185651
A/Black-headed_gull/Republic_of_Georgia/8/2012_H2N7	GenBank		CY185699
A/Mallard/Sweden/1628/2002_H7N7	GenBank		CY186302
A/Mallard/Sweden/1671/2002_H7N7	GenBank		CY186310
A/Mallard/Sweden/1678/2002_H7N7	GenBank		CY186318
A/Mallard/Sweden/1682/2002_H7N7	GenBank		CY186334
A/Mallard/Sweden/1448/2002_H7N7	GenBank		CY186416
A/Ruddy_turnstone/Iceland/1946/2012_H2N7	GenBank		KM213382
A/Duck/Jiangxi/5879/2008_mixed	GenBank		KP287687
A/Duck/Jiangxi/1410/2008_H10N7	GenBank		KP287896
A/Duck/Jiangxi/1591/2008_H10N7	GenBank		KP287912
A/Chicken/Jiangxi/10784/2014_H7N7	GenBank		KP414901
A/Wild_bird/Jiangxi/34458/2013_H7N7	GenBank		KP417105
A/Wild_bird/Jiangxi/35982/2013_H7N7	GenBank		KP417121
