# Supplementary material for: Discordant detection of avian influenza virus subtypes in time and space between poultry and wild birds; Towards improvement of surveillance programs
Source: PLoS One. 2017 Mar 9;12(3):e0173470. doi: 10.1371/journal.pone.0173470 (PMC5344487; doi:10.1371/journal.pone.0173470)
Supplement: S2 Table — We gratefully acknowledge the authors, originating and submitting laboratories of the sequences from the GISAID EpiFlu Database on which this research is based. All submitters may be contacted directly via the GISAID website. (PDF) [file pone.0173470.s004.pdf]

Supporting Information

S2 Table. Details of the low pathogenic avian influenza virus (LPAIV) sequences downloaded from the GISAID EpiFlu Database. We gratefully acknowledge the authors, originating and submitting laboratories of the sequences from GISAID EpiFlu Database on which this research is based.
All submiters of data may be contacted directly via the GISAID website.

Isolate name	Accession number HA segment	Accession number NA segment	Country	Collection Date	Originating Laboratory	Submitting Laboratory	Authors
A/Teal/Egypt/20431-NAMRU3/2003_H1N2	EPI372275		Egypt	22-Dec-2003	U.S. Naval Medical Research Unit No.3	Centers for Disease Control and Prevention	Gerloff, Nancy; Simpson, Natosha; Jones, Joyce; Kis, Zoltan; Bahgat, Verina; Soliman,Atef; Elassal, Emad; Ahmed, Lu'ay; Gaynor, Anne; Cornelius, Claire; Davis,Todd
A/Shoveler/Egypt/00134-NAMRU3/2005_H1N1	EPI372331		Egypt	13-Jan-2005	U.S. Naval Medical Research Unit No.3	Centers for Disease Control and Prevention	Gerloff, Nancy; Simpson, Natosha; Jones, Joyce; Kis, Zoltan; Bahgat, Verina; Soliman,Atef; Elassal, Emad; Ahmed, Lu'ay; Gaynor, Anne; Cornelius, Claire; Davis,Todd
A/Shoveler/Egypt/14029-NAMRU3/2006_H1N1	EPI372378	EPI372377	Egypt	8-Dec-2006	U.S. Naval Medical Research Unit No.3	Centers for Disease Control and Prevention	Gerloff, Nancy; Simpson, Natosha; Jones, Joyce; Kis, Zoltan; Bahgat, Verina; Soliman,Atef; Elassal, Emad; Ahmed, Lu'ay; Gaynor, Anne; Cornelius, Claire; Davis,Todd
A/Teal/Egypt/01351-NAMRU3/2007_H1N1	EPI372466	EPI372465	Egypt	26-Jan-2007	U.S. Naval Medical Research Unit No.3	Centers for Disease Control and Prevention	Gerloff, Nancy; Simpson, Natosha; Jones, Joyce; Kis, Zoltan; Bahgat, Verina; Soliman,Atef; Elassal, Emad; Ahmed, Lu'ay; Gaynor, Anne; Cornelius, Claire; Davis,Todd
A/Teal/Egypt/00677-NAMRU3/2004_H1N1	EPI372528		Egypt	28-Jan-2004	U.S. Naval Medical Research Unit No.3	Centers for Disease Control and Prevention	Gerloff, Nancy; Simpson, Natosha; Jones, Joyce; Kis, Zoltan; Bahgat, Verina; Soliman,Atef; Elassal, Emad; Ahmed, Lu'ay; Gaynor, Anne; Cornelius, Claire; Davis,Todd
A/Goose/Italy/6117/2004_H1N1	EPI178520	EPI178522	Italy	2004*	-	Istituto Zooprofilattico Sperimentale della Lombardia e dell'Emi	-
A/Mallard/Germany/R2843/06_H1N1	EPI222781		Germany	2006*	-	Friedrich-Loeffler-Institut	Fereidouni,SR.
A/Mallard/Germany-RP/R193/09_H1N1	EPI248501	EPI248500	Germany	2009*	-	Friedrich-Loeffler-Institut	-
A/Wild_duck/Germany/WV30/06_H1N1	EPI248514	EPI248512	Germany	2006*	-	Friedrich-Loeffler-Institut	-
A/Mallard/Germany/WV355/07_H1N1	EPI248519	EPI248517	Germany	2007*	-	Friedrich-Loeffler-Institut	-
A/Wild_duck/Germany-NW/R04/08_H1N1	EPI248521		Germany	2008*	-	Friedrich-Loeffler-Institut	-
A/Anas_platyrhynchos/Belgium/09-762/2008_H1N1	EPI257212	EPI257214	Belgium	Nov-2008*	Veterinary and Agrochemical Research Institute	Veterinary and Agrochemical Research Institute	Van Borm,Steven
A/Wild_duck/Korea/CSM38/2004b_H1N1	EPI296244		Korea	Nov-2004*	-	Other database import	Jeong,O.-M.; Kim,Y.-J.; Choi,J.-G.; Kang,H.-M.; Kim,M.-C.; Kwon,J.-H.; Lee,Y.-J.
A/Duck/Italy/7686-11/10_H1N1	EPI301849		Italy	15-Dec-2010	Istituto Zooprofilattico Sperimentale Delle Venezie	Istituto Zooprofilattico Sperimentale Delle Venezie	Monne,I.; Fusaro,A.; Valastro,V.; Schivo,A.; Buratin,A.; Terregino,C.; Capua,I.; Cattoli,G.
A/Pintail/Italy/2703-25/06_H1N1	EPI301856		Italy	5-Sep-2006	Istituto Zooprofilattico Sperimentale Delle Venezie	Istituto Zooprofilattico Sperimentale Delle Venezie	Monne,I.; Fusaro,A.; Valastro,V.; Schivo,A.; Buratin,A.; Terregino,C.; Capua,I.; Cattoli,G.
A/Mallard/Italy/378-49/06_H1N1	EPI301857		Italy	4-May-2006	Istituto Zooprofilattico Sperimentale Delle Venezie	Istituto Zooprofilattico Sperimentale Delle Venezie	Monne,I.; Fusaro,A.; Valastro,V.; Schivo,A.; Buratin,A.; Terregino,C.; Capua,I.; Cattoli,G.
A/Teal/Italy/6323-5/07_H1N1	EPI301858		Italy	30-Nov-2007	Istituto Zooprofilattico Sperimentale Delle Venezie	Istituto Zooprofilattico Sperimentale Delle Venezie	Monne,I.; Fusaro,A.; Valastro,V.; Schivo,A.; Buratin,A.; Terregino,C.; Capua,I.; Cattoli,G.
A/Mallard/Italy/432-21/08_H1N1	EPI301859		Italy	15-Feb-2008	Istituto Zooprofilattico Sperimentale Delle Venezie	Istituto Zooprofilattico Sperimentale Delle Venezie	Monne,I.; Fusaro,A.; Valastro,V.; Schivo,A.; Buratin,A.; Terregino,C.; Capua,I.; Cattoli,G.
A/Shoveler/Italy/6965-6/07_H1N3	EPI301860		Italy	1-Feb-2008	Istituto Zooprofilattico Sperimentale Delle Venezie	Istituto Zooprofilattico Sperimentale Delle Venezie	Monne,I.; Fusaro,A.; Valastro,V.; Schivo,A.; Buratin,A.; Terregino,C.; Capua,I.; Cattoli,G.
A/Avian/Germany-BB/R2859/2009_H6	EPI339183		Germany	2009*	-	Friedrich-Loeffler-Institut	-
A/Goose/Germany-BB/R1625/2008_H6	EPI279941		Germany	2008*	-	Friedrich-Loeffler-Institut	-
A/Ringed_teal/Germany-NRW/R641/2008_H6	EPI279938		Germany	2008*	-	Friedrich-Loeffler-Institut	-
A/Wild_bird/Germany-HH/R1501/2008_H6	EPI279939		Germany	2008*	-	Friedrich-Loeffler-Institut	-
A/Wild_bird/Germany-MV/R1511/2008_H6	EPI279940		Germany	2008*	-	Friedrich-Loeffler-Institut	-
A/Environment/California/NWRC182841-09/2006_H6N1	EPI406091		USA	2006*	-	Other database import	Anderson,T.; Piaggio,T.
A/Environment/California/NWRC183200-14/2006_H6N1	EPI406099		USA	2006*	-	Other database import	Anderson,T.; Piaggio,T.
A/Environment/California/NWRC183274-04/2006_H6N1	EPI406103		USA	2006*	-	Other database import	Anderson,T.; Piaggio,T.
A/Green-winged_teal/Nova_Scotia/14917/2005_H6N1	EPI327397		Canada	14-Sep-2005	-	Other database import	The NIAID Influenza Genome Sequencing Consortium
A/Larus_argentatus/Belgium/02936pcs3/2010_H6N1	EPI345428	EPI345427	Belgium	27-Jan-2010	Veterinary and Agrochemical Research Institute	Veterinary and Agrochemical Research Institute	Van Borm,S.; Rosseel,T.;Lambrecht,B.; Vangeluwe,D.; Vandenbussche,F.; van den Berg,T.
A/Northern_shoveler/California/HKWF115/2007_H6N1	EPI154816		USA	24-Oct-2007	-	Other database import	-
A/Duck/Germany-MV/R871/2010_H6N2	EPI339182		Germany	2010*	-	Friedrich-Loeffler-Institut	-
A/Green-winged_teal/Minnesota/Sg-00199/2007_H6N2	EPI298290		USA	14-Sep-2007	-	Other database import	The NIAID Influenza Genome Sequencing Consortium
A/Green-winged_teal/Minnesota/Sg-00222/2007_H6N2	EPI298322		USA	16-Sep-2007	-	Other database import	The NIAID Influenza Genome Sequencing Consortium
A/Mule_duck/Bulgaria/156/2010_H6N2	EPI574180		Bulgaria	9-Feb-2010	-	Other database import	Marinove-Petkova,A.; Georgiev,G.; Petkov,T.; Darnell,D.; Franks,J.; Walker,D.; Seiler,P.; Danner,A.; Graham,A.; McKenzie,P.; Krauss,S.; Webby,R.J.; Webster,R.G.
A/Mule_duck/Bulgaria/173/2009_H6N2	EPI574206		Bulgaria	12-Jan-2009	-	Other database import	Marinove-Petkova,A.; Georgiev,G.; Petkov,T.; Darnell,D.; Franks,J.; Walker,D.; Seiler,P.; Danner,A.; Graham,A.; McKenzie,P.; Krauss,S.; Webby,R.J.; Webster,R.G.
A/Shoveler/Egypt/13251-NAMRU3/2006_H6N2	EPI372371	EPI372370	Egypt	2-Dec-2006	U.S. Naval Medical Research Unit No.3	Centers for Disease Control and Prevention	Gerloff, Nancy; Simpson, Natosha; Jones, Joyce; Kis, Zoltan; Bahgat, Verina; Soliman,Atef; Elassal, Emad; Ahmed, Lu'ay; Gaynor, Anne; Cornelius, Claire; Davis,Todd
A/Teal/Egypt/13203-NAMRU3/2006_H6N2	EPI372386	EPI372385	Egypt	2-Dec-2006	U.S. Naval Medical Research Unit No.3	Centers for Disease Control and Prevention	Gerloff, Nancy; Simpson, Natosha; Jones, Joyce; Kis, Zoltan; Bahgat, Verina; Soliman,Atef; Elassal, Emad; Ahmed, Lu'ay; Gaynor, Anne; Cornelius, Claire; Davis,Todd
A/Turkey/Germany/R617/2007_H6N2	EPI317612		Germany	2007*	Friedrich-Loeffler-Institut	Friedrich-Loeffler-Institut	Stech,O; Weber,S; Mettenleiter,TC; Stech,J
A/Wild_goose/Germany-BB/R2329/2008_H6N2	EPI397608	EPI397607	Germany	2008*	-	Friedrich-Loeffler-Institut	-
A/Environment/North_Carolina/NWRC183941-06/2006_H6N5	EPI406114		USA	2006*	-	Other database import	Anderson,T.; Piaggio,T.
A/Sentinel_mallard/Germany/Sum156/2007_H6N5	EPI397610	EPI397609	Germany	2007*	-	Friedrich-Loeffler-Institut	-
A/Duck/Germany-NW/R2185/2006_H6N8	EPI397606		Germany	2006*	-	Friedrich-Loeffler-Institut	-
A/Environment/California/NWRC183200-06/2006_H6N8	EPI406098		USA	2006*	-	Other database import	Anderson,T.; Piaggio,T.
A/Goose/Germany/R1767/2007_H6N8	EPI416259		Germany	2007*	-	Friedrich-Loeffler-Institut	-
A/Mallard/Germany-BY/R1353/2010_H6N8	EPI339180		Germany	2010*	-	Friedrich-Loeffler-Institut	-
A/Mule_duck/Bulgaria/365/2010_H6N8	EPI574207		Bulgaria	9-Mar-2010	-	Other database import	Marinove-Petkova,A.; Georgiev,G.; Petkov,T.; Darnell,D.; Franks,J.; Walker,D.; Seiler,P.; Danner,A.; Graham,A.; McKenzie,P.; Krauss,S.; Webby,R.J.; Webster,R.G.
A/Mute_swan/Germany/R2927/2007_H6N8	EPI185339		Germany	2007*	-	Friedrich-Loeffler-Institut	-

Isolate name	Accession number HA segment	Accession number NA segment	Country	Collection Date	Originating Laboratory	Submitting Laboratory	Authors
A/Pink-footed_goose/Iceland/0987/2011_H6N8	EPI476116		Iceland	1-Nov-2011	-	Other database import	Wentworth,D.E.; Halpin,R.A.; Lin,X.; Bera,J.; Akopov,A.; Ransier,A.; Mohan,M.; Fedorova,N.; Tsitrin,T.; Stockwell,T.; Amedeo,P.; Appalla,L.; Bishop,B.; Edworthy,P.; Gupta,N.; Hoover,J.; Katzel,D.; Li,K.; Schobel,S.; Shrivastava,S.; Thovarai,V.; Wang,S.; Bao,Y.; Sanders,R.; Zhdanov,S.; Kiryutin,B.; Lipman,D.J.; Tatusova,T.; Hall,J.S.
A/Mule_duck/Bulgaria/175/2009_H6	EPI574252		Bulgaria	12-Jan-2009	-	Other database import	Marinove-Petkova,A.; Georgiev,G.; Petkov,T.; Darnell,D.; Franks,J.; Walker,D.; Seiler,P.; Danner,A.; Graham,A.; McKenzie,P.; Krauss,S.; Webby,R.J.; Webster,R.G.
A/Mule_duck/Bulgaria/181/2010_H6	EPI574247		Bulgaria	11-Feb-2010	-	Other database import	Marinove-Petkova,A.; Georgiev,G.; Petkov,T.; Darnell,D.; Franks,J.; Walker,D.; Seiler,P.; Danner,A.; Graham,A.; McKenzie,P.; Krauss,S.; Webby,R.J.; Webster,R.G.
A/Chicken/Netherlands/11004875/2011_H7N1	pending	EPI316304	Netherlands	22-Apr-2011	Central Veterinary Institute	Central Veterinary Institute	Heutink R., S. Pritz-Verschuren and G.Koch
A/Duck/Turkey/55/Cetinkaya/49/2006_H7N1	EPI346007		Turkey	2-Mar-2006	Pendik Veterinary Control and Research Institute	Animal and Plant Health Agency (APHA)	Collins,S; Russell,C; Focosi-Snyman,R; Essen,S; Shell,W; Akcadag,B; Iyisan,S; Unsal Baca,A; Reid,S; Manvell,R; Banks,J
A/Guinea_fowl/Italy/407/2008_H7N1	EPI210104		Italy	2008*	Istituto Zooprofilattico Sperimentale Delle Venezie	Istituto Zooprofilattico Sperimentale Delle Venezie	-
A/Mallard/Denmark/58-62-KLSV-119/09_H7N1	EPI492308		Denmark	21-Oct-2009	National Veterinary Institute	Animal and Plant Health Agency (APHA)	Barrass,L; Russell,C; Shell,W; Manvell,R; Jorgensen,P; Reid,S
A/Mallard/Italy/3397-65/2008_H7N1	EPI167297		Italy	2008*	-	Istituto Zooprofilattico Sperimentale Delle Venezie	-
A/Mallard/Italy/6103-5/2007_H7N1	EPI167296		Italy	2007*	-	Istituto Zooprofilattico Sperimentale Delle Venezie	-
A/Mallard/Italy/731/09_H7N1	EPI492522		Italy	7-Jan-2009	Istituto Zooprofilattico Sperimentale Delle Venezie	Animal and Plant Health Agency (APHA)	Agyeman-Dua,E; Russell,C; Shell,W; Manvell,R; Terregino,C; Reid,S
A/Mallard/Italy/794-18/2008_H7N1	EPI167299		Italy	2008*	-	Istituto Zooprofilattico Sperimentale Delle Venezie	-
A/Shoveler/Egypt/00597-NAMRU3/2004_H7N1	EPI372283	EPI372282	Egypt	27-Jan-2004	U.S. Naval Medical Research Unit No.3	Centers for Disease Control and Prevention	Gerloff, Nancy; Simpson, Natosha; Jones, Joyce; Kis, Zoltan; Bahgat, Verina; Soliman,Atef; Elassal, Emad; Ahmed, Lu'ay; Gaynor, Anne; Cornelius, Claire; Davis,Todd
A/Shoveler/Egypt/14879-NAMRU3/2006_H7N1	EPI372363	EPI372362	Egypt	22-Dec-2006	U.S. Naval Medical Research Unit No.3	Centers for Disease Control and Prevention	Gerloff, Nancy; Simpson, Natosha; Jones, Joyce; Kis, Zoltan; Bahgat, Verina; Soliman,Atef; Elassal, Emad; Ahmed, Lu'ay; Gaynor, Anne; Cornelius, Claire; Davis,Todd
A/Tadorna_tadorna/Belgium/3441-P3/2009_H7N1	EPI360900	EPI360901	Belgium	21-Mar-2009	Veterinary and Agrochemical Research Institute	Veterinary and Agrochemical Research Institute	-
A/Teal/Italy/794-3/2008_H7N1	EPI167298		Italy	2008*	-	Istituto Zooprofilattico Sperimentale Delle Venezie	-
A/Chicken/Italy/2240/2003_H7N3	EPI154960		Italy	2003*	-	Istituto Zooprofilattico Sperimentale Delle Venezie	Fusaro, A.; Tassoni, L.; Milani, A.; Salviato, A.; Schivo, A.; Monne, I.; Cattoli, G.
A/Chicken/Italy/2837-54/2007_H7N3	EPI154980		Italy	2007*	-	Istituto Zooprofilattico Sperimentale Delle Venezie	-
A/Chicken/Italy/2837-58/2007_H7N3	EPI154981		Italy	2007*	-	Istituto Zooprofilattico Sperimentale Delle Venezie	-
A/Chicken/Italy/8093/2002_H7N3	EPI154966		Italy	2002*	-	Istituto Zooprofilattico Sperimentale Delle Venezie	Fusaro, A.; Tassoni, L.; Milani, A.; Salviato, A.; Schivo, A.; Monne, I.; Cattoli, G.
A/Guinea_fowl/Italy/1613/2003_H7N3	EPI154959		Italy	2003*	-	Istituto Zooprofilattico Sperimentale Delle Venezie	Fusaro, A.; Tassoni, L.; Milani, A.; Salviato, A.; Schivo, A.; Monne, I.; Cattoli, G.
A/Mallard/Italy/1336/07_H7N3	EPI167295		Italy	2007*	-	Istituto Zooprofilattico Sperimentale Delle Venezie	-
A/Mallard/Italy/6103-12/2007_H7N3	EPI154982		Italy	2007*	-	Istituto Zooprofilattico Sperimentale Delle Venezie	-
A/Mallard/Italy/6104-14/2007_H7N3	EPI167300		Italy	2007*	-	Istituto Zooprofilattico Sperimentale Delle Venezie	-
A/Shoveler/Egypt/00017-NAMRU3/2007_H7N3	EPI372450		Egypt	29-Dec-2006	U.S. Naval Medical Research Unit No.3	Centers for Disease Control and Prevention	Gerloff, Nancy; Simpson, Natosha; Jones, Joyce; Kis, Zoltan; Bahgat, Verina; Soliman,Atef; Elassal, Emad; Ahmed, Lu'ay; Gaynor, Anne; Cornelius, Claire; Davis,Todd
A/Shoveler/Egypt/00241-NAMRU3/2007_H7N3	EPI372418		Egypt	5-Jan-2007	U.S. Naval Medical Research Unit No.3	Centers for Disease Control and Prevention	Gerloff, Nancy; Simpson, Natosha; Jones, Joyce; Kis, Zoltan; Bahgat, Verina; Soliman,Atef; Elassal, Emad; Ahmed, Lu'ay; Gaynor, Anne; Cornelius, Claire; Davis,Todd
A/Turkey/Italy/2963/2003_H7N3	EPI243279		Italy	23-May-2003	-	Istituto Zooprofilattico Sperimentale Delle Venezie	-
A/Turkey/Italy/8307/2002_H7N3	EPI154967		Italy	2002*	-	Istituto Zooprofilattico Sperimentale Delle Venezie	Fusaro, A.; Tassoni, L.; Milani, A.; Salviato, A.; Schivo, A.; Monne, I.; Cattoli, G.
A/Swan/Germany/R736/06_H7N4	EPI492517		Germany	Mar-2003*	Friedrich-Loeffler-Institut	Animal and Plant Health Agency (APHA)	Hanna,A; Russell,C; Shell,W; Harder,T; Grund,C; Starick,E; Manvell,R
A/Teal/Italy/11VIR-792/11_H7N6	EPI492520		Italy	31-Jan-2011	Istituto Zooprofilattico Sperimentale Delle Venezie	Animal and Plant Health Agency (APHA)	Collins,S; Hanna,A; Essen,S; Focosi-Snyman,R; Manvell, R; Terregino,C; Reid,S
A/Branta_canadensis/Belgium/13000-9-2/2010_H7N7	EPI360902		Belgium	2010*	Veterinary and Agrochemical Research Institute	Veterinary and Agrochemical Research Institute	-
A/Chicken/Germany-NI/R874/2010_H7N7	EPI302178		Germany	2010*	-	Friedrich-Loeffler-Institut	-
A/Chicken/Germany/R1362/11_H7N7	EPI492511		Germany	May-2011*	Friedrich-Loeffler-Institut	Animal and Plant Health Agency (APHA)	Agyeman-Dua,E; Russell,C; Shell,W; Manvell,R; Harder,T; Grund,C; Starick,E; Fereidouni,S; Reid,S
A/Chicken/Netherlands/11011326/2011_H7N7	pending	EPI325342	Netherlands	21-Jun-2006	Central Veterinary Institute	Central Veterinary Institute	Pritz-Vershuren, Sylvia J.; Heutink, Rene; Koch, Guus
A/Chicken/Netherlands/12014794/2012_H7N7	EPI390921	EPI390922	Netherlands	9-Aug-2012	Central Veterinary Institute	Central Veterinary Institute	Heutink,Rene; Pritz-Verschuren,Sylvia; Bouwstra,Ruth; Koch, Guus
A/Egyptian_goose/Egypt/05588-NAMRU3/2006_H7N7	EPI372394	EPI372393	Egypt	7-Apr-2006	U.S. Naval Medical Research Unit No.3	Centers for Disease Control and Prevention	Gerloff, Nancy; Simpson, Natosha; Jones, Joyce; Kis, Zoltan; Bahgat, Verina; Soliman,Atef; Elassal, Emad; Ahmed, Lu'ay; Gaynor, Anne; Cornelius, Claire; Davis,Todd
A/Mallard/Denmark/303878-1S/13_H7N7	EPI492307		Denmark	26-May-2013	National Veterinary Institute	Animal and Plant Health Agency (APHA)	Puranik,A; Hanna,A; Essen,S; Focosi-Snyman,R; Manvell,R; Hjulsager,C; Trebbien,R; Breum,S; Larsen,L; Reid,S
A/Mallard/Italy/11VIR-540/11_H7N7	EPI492519		Italy	10-Jan-2011	Istituto Zooprofilattico Sperimentale Delle Venezie	Animal and Plant Health Agency (APHA)	Collins,S; Hanna,A; Essen,S; Focosi-Snyman,R; Manvell, R; Terregino,C; Reid,S

Isolate name	Accession number HA segment	Accession number NA segment	Country	Collection Date	Originating Laboratory	Submitting Laboratory	Authors
A/Mallard/Poland/01/08_H7N7	EPI169422	EPI169423	Poland	28-Dec-2007	National Veterinary Research Institute	National Veterinary Research Institute	-
A/Mallard/Poland/41/09_H7N7	EPI211188		Poland	16-Feb-2009	National Veterinary Research Institute	National Veterinary Research Institute	-
A/Mallard/Poland/446/09_H7N7	EPI254381	EPI254382	Poland	27-Dec-2009	National Veterinary Research Institute	National Veterinary Research Institute	Smietanka,K; Pikula,A; Minta,Z
A/Pochard/Germany/R916/06_H7N7	EPI492516		Germany	Mar-2006*	Friedrich-Loeffler-Institut	Animal and Plant Health Agency (APHA)	Hanna,A; Russell,C; Shell,W; Harder,T; Grund,C; Starick,E; Manvell,R
A/Shoveler/Egypt/09864-NAMRU3/2004_H7N7	EPI372323	EPI372322	Egypt	22-Dec-2004	U.S. Naval Medical Research Unit No.3	Centers for Disease Control and Prevention	Gerloff, Nancy; Simpson, Natosha; Jones, Joyce; Kis, Zoltan; Bahgat, Verina; Soliman,Atef; Elassal, Emad; Ahmed, Lu'ay; Gaynor, Anne; Cornelius, Claire; Davis,Todd
A/Swan/Germany/R57/06_H7N7	EPI492518		Germany	Jan-2006*	Friedrich-Loeffler-Institut	Animal and Plant Health Agency (APHA)	Hanna,A; Russell,C; Shell,W; Harder,T; Grund,C; Starick,E; Manvell,R
A/Teal/Egypt/00835-NAMRU3/2004_H7N7	EPI372307		Egypt	18-Feb-2004	U.S. Naval Medical Research Unit No.3	Centers for Disease Control and Prevention	Gerloff, Nancy; Simpson, Natosha; Jones, Joyce; Kis, Zoltan; Bahgat, Verina; Soliman,Atef; Elassal, Emad; Ahmed, Lu'ay; Gaynor, Anne; Cornelius, Claire; Davis,Todd
A/Turkey/Germany-NW/R655/2009_H7N7	EPI356351		Germany	2009*	-	Friedrich-Loeffler-Institut	-
A/Shoveler/Egypt/00215-NAMRU3/2007_H7N9	EPI372410		Egypt	5-Jan-2007	U.S. Naval Medical Research Unit No.3	Centers for Disease Control and Prevention	Gerloff, Nancy; Simpson, Natosha; Jones, Joyce; Kis, Zoltan; Bahgat, Verina; Soliman,Atef; Elassal, Emad; Ahmed, Lu'ay; Gaynor, Anne; Cornelius, Claire; Davis,Todd
A/Chicken/Italy/13VIR4527-11/2013_H7N7	EPI677999	EPI677998	Italy	13-Aug-2013	-	Istituto Zooprofilattico Sperimentale Delle Venezie	Fusaro, A.; Tassoni, L.; Milani, A.; Hughes, J.; Salviato, A.; Murcia, P.; Massi, P.; Bonfanti, L.; Marangon, S.; Cattoli, G.; Monne, I.
A/Northern_shoveler/Mississippi/11OS5900/2011_H8N1	EPI512596		USA	8-Dec-2011	-	Other database import	Wentworth,D.E.; Halpin,R.A.; Lin,X.; Bera,J.; Akopov,A.; Ransier,A.; Mohan,M.; Fedorova,N.; Tsitrin,T.; Puri,V.; Stockwell,T.; Amedeo,P.; Appalla,L.; Bishop,B.; Edworthy,P.; Gupta,N.; Hoover,J.; Katzel,D.; Li,K.; Schobel,S.; Shrivastava,S.; Thovarai,V.; Wang,S.; Nolting,J.M.; Fries,A.C.; Bowman,A.S.; Bao,Y.; Sanders,R.; Zhdanov,S.; Kiryutin,B.; Lipman,D.J.; Tatusova,T.; Slemons,R.
A/American_black_duck/Nova_Scotia/02043/2007_H8N4	EPI404484		Canada	8-Aug-2007	-	Other database import	Wentworth,D.E.; Dugan,V.; Halpin,R.; Lin,X.; Bera,J.; Ghedin,E.; Fedorova,N.; Overton,L.; Tsitrin,T.; Stockwell,T.; Amedeo,P.; Bishop,B.; Chen,H.;
A/American_green-winged_teal/California/44287-373/2007_H8N4	EPI292438		USA	27-Jan-2007	-	Other database import	The NIAID Influenza Genome Sequencing Consortium
A/American_green-winged_teal/Interior_Alaska/9BM5045R0/2009_H8N4	EPI433069	EPI433100	USA	29-Jul-2009	-	Other database import	Wentworth,D.E.; Dugan,V.; Halpin,R.; Lin,X.; Bera,J.; Ghedin,E.; Fedorova,N.; Overton,L.; Tsitrin,T.; Stockwell,T.; Amedeo,P.; Bishop,B.; Chen,H.; Edworthy,P.; Gupta,N.; Katzel,D.; Li,K.; Schobel,S.; Shrivastava,S.; Thovarai,V.; Wang,S.; Runstadler,J.; Lindberg,M.; Huettmann,F.; Petrula,M.; Meixell,B.; Gingrich,J.P.; Gildehaus,L.A.; Vick,L.; Kokx,K.; Dillon,D.; Aldehoff,F.; Felker,E.P.; Marcotte,R.W.; Schmidt,J.C.; Moore,J.R.; Gerdes,K.E.; Bao,Y.; Sanders,R.; Dernovoy,D.; Kiryutin,B.; Lipman,D.J.; Tatusova,T.
A/Environment/Pennsylvania/NWRC182092-24/2006_H8N4	EPI406002		USA	2006*	-	Other database import	Anderson,T.; Piaggio,T.
A/Garganey/Ukraine/05835-NAMRU3/2006_H8N4	EPI372512	EPI372511	Ukraine	13-Aug-2006	U.S. Naval Medical Research Unit No.3	Centers for Disease Control and Prevention	Gerloff, Nancy; Simpson, Natosha; Jones, Joyce; Kis, Zoltan; Bahgat, Verina; Soliman,Atef; Elassal, Emad; Ahmed, Lu'ay; Gaynor, Anne; Cornelius, Claire; Davis,Todd
A/Mallard/Interior_Alaska/8BM1966R1/2008_H8N4	EPI299970		USA	8-Aug-2008	-	Other database import	The NIAID Influenza Genome Sequencing Consortium
A/Mallard/Interior_Alaska/8MP0547/2008_H8N4	EPI299411		USA	11-Aug-2008	-	Other database import	The NIAID Influenza Genome Sequencing Consortium
A/Mallard/Interior_Alaska/9BM10537R0/2009_H8N4	EPI436880		USA	2009*	-	Other database import	Wentworth,D.E.; Dugan,V.; Halpin,R.; Lin,X.; Bera,J.; Ghedin,E.; Fedorova,N.; Overton,L.; Tsitrin,T.; Stockwell,T.; Amedeo,P.; Bishop,B.; Chen,H.;
A/Mallard/Interior_Alaska/9BM8389R0/2009_H8N4	EPI452280	EPI452282	USA	2009*	-	Other database import	-
A/Mallard/Minnesota/Sg-00675/2008_H8N4	EPI188649		USA	3-Aug-2008	-	Other database import	Sreevatsan,S.; Ramakrishnan,M.A.; Wang,P.; Anderson,T.L.; Jindal,N.; Chander,Y.; Goyal,S.M.; Osterholm,M.T.
A/Northern_pintail/Interior_Alaska/8BM2011R1/2008_H8N4	EPI299978		USA	9-Aug-2008	-	Other database import	The NIAID Influenza Genome Sequencing Consortium
A/Northern_pintail/Interior_Alaska/8BM2046R1/2008_H8N4	EPI299419		USA	9-Aug-2008	-	Other database import	The NIAID Influenza Genome Sequencing Consortium
A/Northern_pintail/Interior_Alaska/8BM2621R1/2008_H8N4	EPI299686		USA	14-Aug-2008	-	Other database import	The NIAID Influenza Genome Sequencing Consortium
A/Northern_pintail/Interior_Alaska/8BM2987/2008_H8N4	EPI299122		USA	17-Aug-2008	-	Other database import	The NIAID Influenza Genome Sequencing Consortium
A/Northern_pintail/Interior_Alaska/8BM3088/2008_H8N4	EPI299146		USA	18-Aug-2008	-	Other database import	The NIAID Influenza Genome Sequencing Consortium
A/Northern_pintail/Interior_Alaska/8BM3091/2008_H8N4	EPI299694		USA	18-Aug-2008	-	Other database import	The NIAID Influenza Genome Sequencing Consortium
A/Northern_pintail/Interior_Alaska/8BM3137/2008_H8N4	EPI299702		USA	18-Aug-2008	-	Other database import	The NIAID Influenza Genome Sequencing Consortium
A/Northern_pintail/Interior_Alaska/8MP0689/2008_H8N4	EPI299090		USA	13-Aug-2008	-	Other database import	The NIAID Influenza Genome Sequencing Consortium
A/Northern_pintail/Interior_Alaska/9BM11556R0/2009_H8N4	EPI452128		USA	2009*	-	Other database import	Wentworth,D.E.; Dugan,V.; Halpin,R.; Lin,X.; Bera,J.; Ghedin,E.; Fedorova,N.; Overton,L.; Tsitrin,T.; Stockwell,T.; Amedeo,P.; Bishop,B.; Chen,H.;
A/Northern_pintail/Interior_Alaska/9BM11643R0/2009_H8N4	EPI452142	EPI452144	USA	2009*	-	Other database import	Wentworth,D.E.; Dugan,V.; Halpin,R.; Lin,X.; Bera,J.; Ghedin,E.; Fedorova,N.; Overton,L.; Tsitrin,T.; Stockwell,T.; Amedeo,P.; Bishop,B.; Chen,H.;
A/Northern_pintail/Interior_Alaska/9BM6510R0/2009_H8N4	EPI452001	EPI452003	USA	2009*	-	Other database import	Wentworth,D.E.; Dugan,V.; Halpin,R.; Lin,X.; Bera,J.; Ghedin,E.; Fedorova,N.; Overton,L.; Tsitrin,T.; Stockwell,T.; Amedeo,P.; Bishop,B.; Chen,H.; Edworthy,P.; Gupta,N.; Katzel,D.; Li,K.; Schobel,S.; Shrivastava,S.; Thovarai,V.; Wang,S.; Runstadler,J.; Lindberg,M.; Huettmann,F.; Petrula,M.; Meixell,B.; Gingrich,J.P.; Gildehaus,L.A.; Vick,L.; Kokx,K.; Dillon,D.; Aldehoff,F.; Felker,E.P.; Marcotte,R.W.; Schmidt,J.C.; Moore,J.R.; Gerdes,K.E.; Bao,Y.; Sanders,R.; Dernovoy,D.; Kiryutin,B.; Lipman,D.J.; Tatusova,T.
A/Northern_pintail/Interior_Alaska/9BM7152R2/2009_H8N4	EPI452015	EPI452017	USA	2009*	-	Other database import	-
A/Northern_pintail/Interior_Alaska/9BM7240R0/2009_H8N4	EPI452050		USA	2009*	-	Other database import	Wentworth,D.E.; Dugan,V.; Halpin,R.; Lin,X.; Bera,J.; Ghedin,E.; Fedorova,N.; Overton,L.; Tsitrin,T.; Stockwell,T.; Amedeo,P.; Bishop,B.; Chen,H.;
A/Northern_pintail/Interior_Alaska/9BM7882R0/2009_H8N4	EPI452219		USA	2009*	-	Other database import	Wentworth,D.E.; Dugan,V.; Halpin,R.; Lin,X.; Bera,J.; Ghedin,E.; Fedorova,N.; Overton,L.; Tsitrin,T.; Stockwell,T.; Amedeo,P.; Bishop,B.; Chen,H.;
A/Northern_pintail/Interior_Alaska/9BM8109R0/2009_H8N4	EPI436940		USA	2009*	-	Other database import	Wentworth,D.E.; Dugan,V.; Halpin,R.; Lin,X.; Bera,J.; Ghedin,E.; Fedorova,N.; Overton,L.; Tsitrin,T.; Stockwell,T.; Amedeo,P.; Bishop,B.; Chen,H.;
A/Northern_pintail/Interior_Alaska/9BM8237R0/2009_H8N4	EPI452226		USA	2009*	-	Other database import	Wentworth,D.E.; Dugan,V.; Halpin,R.; Lin,X.; Bera,J.; Ghedin,E.; Fedorova,N.; Overton,L.; Tsitrin,T.; Stockwell,T.; Amedeo,P.; Bishop,B.; Chen,H.;
A/Northern_pintail/Interior_Alaska/9BM8967R0/2009_H8N4	EPI436976		USA	2009*	-	Other database import	Wentworth,D.E.; Dugan,V.; Halpin,R.; Lin,X.; Bera,J.; Ghedin,E.; Fedorova,N.; Overton,L.; Tsitrin,T.; Stockwell,T.; Amedeo,P.; Bishop,B.; Chen,H.;
A/Northern_shoveler/California/AKS273/2007_H8N4	EPI178742		USA	1-Dec-2007	-	Other database import	-

Isolate name	Accession number HA segment	Accession number NA segment	Country	Collection Date	Originating Laboratory	Submitting Laboratory	Authors
A/Northern_shoveler/California/HKWF1203/2007_H8N4	EPI222411		USA	5-Dec-2007	-	Other database import	Cardona,C.; Boyce,W.M.; Nelson,J.; Anchell,N.; Dao,N.; Anunciacion,J.; Cai,H.; Green,L.; Detter,C.; Kiss,H.; Dannen,M.
A/Northern_shoveler/California/HKWF1204/2007_H8N4	EPI178782		USA	5-Dec-2007	-	Other database import	-
A/Northern_shoveler/California/HKWF1325/2007_H8N4	EPI160322		USA	9-Dec-2007	-	Other database import	-
A/Northern_shoveler/Interior_Alaska/9BM2925R0/2009_H8N4	EPI432837		USA	16-Jul-2009	-	Other database import	Wentworth,D.E.; Dugan,V.; Halpin,R.; Lin,X.; Bera,J.; Ghedin,E.; Fedorova,N.; Overton,L.; Tsitrin,T.; Stockwell,T.; Amedeo,P.; Bishop,B.; Chen,H.;
A/Northern_shoveler/Minnesota/Sg-00648/2008_H8N4	EPI449391		USA	1-Aug-2008	-	Other database import	Wentworth,D.E.; Dugan,V.; Halpin,R.; Lin,X.; Bera,J.; Wester,E.; Ghedin,E.; Fedorova,N.; Tsitrin,T.; Stockwell,T.; Amedeo,P.; Bishop,B.; Edworthy,P.; Gupta,N.; Katzel,D.; Li,K.; Schobel,S.; Shrivastava,S.; Thovarai,V.; Wang,S.; Lebarbenchon,C.; Sreevatsan,S.; Poulson,B.; Yang,M.; Stallknecht,D.; Bao,Y.; Sanders,R.; Dernovoy,D.; Kiryutin,B.; Lipman,D.J.; Tatusova,T.
A/Teal/Northern_Ireland/14567-10-5257/2007_H9N1	EPI383878		Ireland	2007*	-	Other database import	Slomka,M.J.; Hanna,A.; Mahmood,S.; Govil,J.; Krill,D.; Manvell,R.J.; Shell,W.; Arnold,M.E.; Banks,J.; Brown,I.H.
A/Common_coot/Poland/88/13_H9N2	EPI505111		Poland	22-Sep-2013	-	National Veterinary Research Institut Poland, PIWet-PIB	-
A/Mallard/Iran/C364/2007_H9N2	EPI302559		Iran	2007*	-	Other database import	Fereidouni,SR.
A/Turkey/England/13437/2013_H9N2	EPI585514		England	14-Apr-2013	Animal and Plant Health Agency (APHA)	Animal and Plant Health Agency (APHA)	Reid, SM; Banks, J; Ceeraz, V; Cox, WJ; Howard, WA; Puranik, A; Collins, S; Seekings, A; Manvell, R; Irvine, RM; Brown, IH
A/Turkey/England/13538/2013_H9N2	EPI585519		England	14-Apr-2013	Animal and Plant Health Agency (APHA)	Animal and Plant Health Agency (APHA)	Reid, SM; Banks, J; Ceeraz, V; Cox, WJ; Howard, WA; Puranik, A; Collins, S; Seekings, A; Manvell, R; Irvine, RM; Brown, IH
A/Turkey/Poland/14/13_H9N2	EPI500817		Poland	23-Apr-2013	-	National Veterinary Research Institut Poland, PIWet-PIB	-
A/Turkey/Poland/20/13_H9N2	EPI505105		Poland	15-May-2013	-	National Veterinary Research Institut Poland, PIWet-PIB	-
A/Ruddy_turnstone/New_Jersey/AI03-128/2003_H9N7	EPI454831		USA	21-May-2003	-	Other database import	Wentworth,D.E.; Dugan,V.; Halpin,R.; Lin,X.; Bera,J.; Wester,E.; Ghedin,E.; Fedorova,N.; Tsitrin,T.; Stockwell,T.; Amedeo,P.; Bishop,B.; Edworthy,P.; Gupta,N.; Katzel,D.; Li,K.; Schobel,S.; Shrivastava,S.; Thovarai,V.; Wang,S.; Lebarbenchon,C.; Sreevatsan,S.; Poulson,B.; Yang,M.; Stallknecht,D.; Bao,Y.; Sanders,R.; Dernovoy,D.; Kiryutin,B.; Lipman,D.J.; Tatusova,T.
A/Ruddy_turnstone/New_Jersey/AI03-444/2003_H9N9	EPI454810		USA	20-May-2003	-	Other database import	Wentworth,D.E.; Dugan,V.; Halpin,R.; Lin,X.; Bera,J.; Wester,E.; Ghedin,E.; Fedorova,N.; Tsitrin,T.; Stockwell,T.; Amedeo,P.; Bishop,B.; Edworthy,P.; Gupta,N.; Katzel,D.; Li,K.; Schobel,S.; Shrivastava,S.; Thovarai,V.; Wang,S.; Lebarbenchon,C.; Sreevatsan,S.; Poulson,B.; Yang,M.; Stallknecht,D.; Bao,Y.; Sanders,R.; Dernovoy,D.; Kiryutin,B.; Lipman,D.J.; Tatusova,T.
A/Duck/Tsukuba/574/2006_H10N1	EPI356629		Japan	2006*	-	Other database import	Tsukamoto,K.; Javier,P.; Shishido,M.; Noguchi,D.; Pearce,J.; Kang,H.; Jeong,O.; Lee,Y.; Nakanishi,K.; Ashizawa,T.
A/Shoveler/Egypt/00006-NAMRU3/2007_H10N1	EPI372442		Egypt	29-Dec-2006	U.S. Naval Medical Research Unit No.3	Centers for Disease Control and Prevention	Gerloff, Nancy; Simpson, Natosha; Jones, Joyce; Kis, Zoltan; Bahgat, Verina; Soliman,Atef; Elassal, Emad; Ahmed, Lu'ay; Gaynor, Anne; Cornelius, Claire; Davis,Todd
A/Teal/Egypt/12908-NAMRU3/2005_H10N1	EPI372481	EPI372480	Egypt	21-Nov-2005	U.S. Naval Medical Research Unit No.3	Centers for Disease Control and Prevention	Gerloff, Nancy; Simpson, Natosha; Jones, Joyce; Kis, Zoltan; Bahgat, Verina; Soliman,Atef; Elassal, Emad; Ahmed, Lu'ay; Gaynor, Anne; Cornelius, Claire; Davis,Todd
A/Wild_bird/Korea/A323/2009_H10N1	EPI387876		Korea	Nov-2009*	-	Other database import	Kim,H.R.; Lee,Y.J.; Oem,J.K.; Bae,Y.C.; Kang,M.S.; Kang,H.M.; Choi,J.G.; Park,C.K.; Kwon,Y.K.
A/Duck/Hokkaido/W87/2007_H10N2	EPI161527		Japan	2007*	-	Other database import	-
A/Duck/Hunan/S11205/2012_H10N3	EPI461563		China	25-Mar-2012	-	Other database import	Deng,G.; Tan,D.; Shi,J.; Cui,P.; Jiang,Y.; Liu,L.; Tian,G.; Kawaoka,Y.; Li,C.; Chen,H.
A/Duck/Thailand/LM-CU4747/2009_H10N3	EPI314742		Thailand	11-2009*	-	Other database import	Amonsin,A.; Lapkuntod,J.; Suradhat,S.; Tantilertcharoen,R.; Bunpapong,N.; Boonyapisitsopa,S.; Wongphatcharachai,M.; Wisedchanwet,T.; Poovorawan,Y.; Sasipreeyajan,J.; Thanawongnuwech,R.
A/Duck/Thailand/LM-CU4753/2009_H10N3	EPI314746		Thailand	11-2009*	-	Other database import	Amonsin,A.; Lapkuntod,J.; Suradhat,S.; Tantilertcharoen,R.; Bunpapong,N.; Boonyapisitsopa,S.; Wongphatcharachai,M.; Wisedchanwet,T.; Poovorawan,Y.; Sasipreeyajan,J.; Thanawongnuwech,R.
A/Muscovy_duck/Thailand/CU-LM4754/2009_H10N3	EPI256770		Thailand	11-2009*	-	Other database import	Amonsin,A.
A/Duck/Italy/268302/2004_H10N4	EPI178493	EPI178495	Italy	2004*	-	Istituto Zooprofilattico Sperimentale della Lombardia e dell'Emi	-
A/Pied_avocet/Ukraine/05848-NAMRU3/2006_H10N4	EPI372496	EPI372495	Ukraine	1-May-2006	U.S. Naval Medical Research Unit No.3	Centers for Disease Control and Prevention	Gerloff, Nancy; Simpson, Natosha; Jones, Joyce; Kis, Zoltan; Bahgat, Verina; Soliman,Atef; Elassal, Emad; Ahmed, Lu'ay; Gaynor, Anne; Cornelius, Claire; Davis,Todd
A/Shoveler/Egypt/01574-NAMRU3/2007_H10N4	EPI372458	EPI372457	Egypt	9-Feb-2007	U.S. Naval Medical Research Unit No.3	Centers for Disease Control and Prevention	Gerloff, Nancy; Simpson, Natosha; Jones, Joyce; Kis, Zoltan; Bahgat, Verina; Soliman,Atef; Elassal, Emad; Ahmed, Lu'ay; Gaynor, Anne; Cornelius, Claire; Davis,Todd
A/Long-tailed_duck/Wisconsin/10OS3919/2010_H10N6	EPI419336		USA	16-Nov-2010	-	Other database import	Wentworth,D.E.; Dugan,V.; Halpin,R.; Lin,X.; Bera,J.; Wester,E.; Ghedin,E.; Fedorova,N.; Tsitrin,T.; Stockwell,T.; Amedeo,P.; Bishop,B.; Edworthy,P.; Gupta,N.; Katzel,D.; Li,K.; Schobel,S.; Shrivastava,S.; Thovarai,V.; Wang,S.; Lebarbenchon,C.; Sreevatsan,S.; Poulson,B.; Yang,M.; Stallknecht,D.; Bao,Y.; Sanders,R.; Dernovoy,D.; Kiryutin,B.; Lipman,D.J.; Tatusova,T.
A/Mallard/Denmark/16109-4/2011-11-14_H10N6	EPI541472		Denmark	14-Nov-2011	Technical University of Denmark	Technical University of Denmark	Hjulsager, Charlotte; Breum, Solvej; Trebbien, Ramona; Larsen, Lars E
A/Anas_platyrhynchos/Camargue/091863/09_H10N7	EPI332944	EPI332955	France	20-May-2009	Station Biologique "Tour du Valat"	Institut Pasteur	VITTECOQ Marion, GRANDHOMME Viviane
A/Avian/Israel/232/2001_H10N7	EPI456969		Israel	Feb-2001*	-	Other database import	Shkoda,I.; Panshin,A.; Shihmanter,E.; Lapin,K.; Lipkind,M.
A/Duck/Italy/62330/2006_H10N7	EPI178528		Italy	2006*	-	Istituto Zooprofilattico Sperimentale Delle Venezie	-
A/Duck/Italy/73383/2006_H10N7	EPI174777		Italy	2006*	-	Istituto Zooprofilattico Sperimentale Delle Venezie	-
A/Harbor_seal/Denmark/14-5061-1lu/2014-07_H10N7	EPI541474		Denmark	Jul-2014*	Technical University of Denmark	Technical University of Denmark	Krog, Jesper Schak; Hjulsager, Charlotte; Larsen, Lars E
A/Mallard/Egypt/EMC-4/2012_H10N7	EPI552755		Egypt	9-Mar-2012	Erasmus Medical Center	Erasmus Medical Center	Bodewes, Rogier; Bestebroer, Theo M.; Van der Vries, Erhard; Verhagen, Josanne H.; Koopmans, Marion P.; Fouchier, Ron A.M.; Wohlsein, Peter; Siebert, Ursula; Baumgärtner, Wolfgang; Osterhaus, Albert D.M.E.

Isolate name	Accession number HA segment	Accession number NA segment	Country	Collection Date	Originating Laboratory	Submitting Laboratory	Authors
A/Mallard/Netherlands/1/2012_H10N7	EPI552756		Netherlands	14-Jan-2012	Erasmus Medical Center	Erasmus Medical Center	Bodewes, Rogier; Bestebroer, Theo M.; Van der Vries, Erhard; Verhagen, Josanne H.; Koopmans, Marion P.; Fouchier, Ron A.M.; Wohlsein, Peter; Siebert, Ursula; Baumgärtner, Wolfgang; Osterhaus, Albert D.M.E.
A/Mallard/Netherlands/1/2014_H10N7	EPI552751		Netherlands	17-Feb-2014	Erasmus Medical Center	Erasmus Medical Center	Bodewes, Rogier; Bestebroer, Theo M.; Van der Vries, Erhard; Verhagen, Josanne H.; Koopmans, Marion P.; Fouchier, Ron A.M.; Wohlsein, Peter; Siebert, Ursula; Baumgärtner, Wolfgang; Osterhaus, Albert D.M.E.
A/Northern_pintail/Egypt/EMC-1/2012_H10N7	EPI552754		Egypt	20-Jan-2012	Erasmus Medical Center	Erasmus Medical Center	Bodewes, Rogier; Bestebroer, Theo M.; Van der Vries, Erhard; Verhagen, Josanne H.; Koopmans, Marion P.; Fouchier, Ron A.M.; Wohlsein, Peter; Siebert, Ursula; Baumgärtner, Wolfgang; Osterhaus, Albert D.M.E.
A/Seal/Sweden/SVA0546/2014_H10N7	EPI545212		Sweden	16-Apr-2014	Swedish Museum of Natural History	National Veterinary Institute	Siamak,Zohari; Aleksija,Neimanis; Tero,Härkönen; Charlotta,Moraeus; Jean-Francois,Vallachare
A/Shoveler/Egypt/00600-NAMRU3/2004_H10N7	EPI372291		Egypt	27-Jan-2004	U.S. Naval Medical Research Unit No.3	Centers for Disease Control and Prevention	Gerloff, Nancy; Simpson, Natosha; Jones, Joyce; Kis, Zoltan; Bahgat, Verina; Soliman,Atef; Elassal, Emad; Ahmed, Lu'ay; Gaynor, Anne; Cornelius, Claire; Davis,Todd
A/Shoveler/Egypt/01198-NAMRU3/2007_H10N7	EPI372402		Egypt	19-Jan-2007	U.S. Naval Medical Research Unit No.3	Centers for Disease Control and Prevention	Gerloff, Nancy; Simpson, Natosha; Jones, Joyce; Kis, Zoltan; Bahgat, Verina; Soliman,Atef; Elassal, Emad; Ahmed, Lu'ay; Gaynor, Anne; Cornelius, Claire; Davis,Todd
A/Shoveler/Egypt/09781-NAMRU3/2004_H10N7	EPI372339	EPI372338	Egypt	18-Dec-2004	U.S. Naval Medical Research Unit No.3	Centers for Disease Control and Prevention	Gerloff, Nancy; Simpson, Natosha; Jones, Joyce; Kis, Zoltan; Bahgat, Verina; Soliman,Atef; Elassal, Emad; Ahmed, Lu'ay; Gaynor, Anne; Cornelius, Claire; Davis,Todd
A/Teal/Egypt/01207-NAMRU3/2007_H10N7	EPI372426	EPI372425	Egypt	19-Jan-2007	U.S. Naval Medical Research Unit No.3	Centers for Disease Control and Prevention	Gerloff, Nancy; Simpson, Natosha; Jones, Joyce; Kis, Zoltan; Bahgat, Verina; Soliman,Atef; Elassal, Emad; Ahmed, Lu'ay; Gaynor, Anne; Cornelius, Claire; Davis,Todd
A/Seal/Sweden/SVA0824/2014_/H10N7_H10N7	EPI547696		Sweden	29-Aug-2014	Swedish Museum of Natural History	National Veterinary Institute	Siamak,Zohari; Aleksija,Neimanis; Tero,Härkönen; Charlotta,Moraeus; Jean-Francois,Vallachare
A/Chicken/77/Jiangxi/2014_H10N8	EPI537463		China	9-Jan-2014	-	Tsinghua University	-
A/Chicken/Jiangxi/102/2013_H10N8	EPI530542		China	8-Dec-2013	South China Agricultural University	South China Agricultural University	Wenbao,Qi; Xianfeng,Zhou; Lihong,Huang; Huanan,Li; Qian,Li; Ming,Liao; Mingbin,Liu
A/Environment/Dongting_Lake/Hunan/3-9/2007_H10N8	EPI221966		China	2007*	-	Other database import	Zhang,H.; Chen, Z.
A/Environment/Jiangxi/03366/2013_H10N8	EPI530386		China	8-Dec-2013	WHO Chinese National Influenza Center	WHO Chinese National Influenza Center	Wang,Dayan; Gao,Rongbao; Yang,Lei; Zhou,Shumei; Li,Xiyan; Zhou,Jianfang; Zhu,Wenfei; Guo,Junfeng; Li,Xiaodan; Dong,Jie; Huang,Weijuan; Zhang,Ye; Dong,Libo; Zhao,Xiang; Lu,Jian; Lan,Yu; Shu,Yuelong
A/Environment/Jiangxi/03367/2013_H10N8	EPI530394		China	8-Dec-2013	WHO Chinese National Influenza Center	WHO Chinese National Influenza Center	Wang,Dayan; Gao,Rongbao; Yang,Lei; Zhou,Shumei; Li,Xiyan; Zhou,Jianfang; Zhu,Wenfei; Guo,Junfeng; Li,Xiaodan; Dong,Jie; Huang,Weijuan; Zhang,Ye; Dong,Libo; Zhao,Xiang; Lu,Jian; Lan,Yu; Shu,Yuelong
A/Environment/Jiangxi/03413/2013_H10N8	EPI530402		China	11-Dec-2013	WHO Chinese National Influenza Center	WHO Chinese National Influenza Center	Wang,Dayan; Gao,Rongbao; Yang,Lei; Zhou,Shumei; Li,Xiyan; Zhou,Jianfang; Zhu,Wenfei; Guo,Junfeng; Li,Xiaodan; Dong,Jie; Huang,Weijuan; Zhang,Ye; Dong,Libo; Zhao,Xiang; Lu,Jian; Lan,Yu; Shu,Yuelong
A/Environment/Jiangxi/03489/2013_H10N8	EPI530410		China	27-Dec-2013	WHO Chinese National Influenza Center	WHO Chinese National Influenza Center	Wang,Dayan; Gao,Rongbao; Yang,Lei; Zhou,Shumei; Li,Xiyan; Zhou,Jianfang; Zhu,Wenfei; Guo,Junfeng; Li,Xiaodan; Dong,Jie; Huang,Weijuan; Zhang,Ye; Dong,Libo; Zhao,Xiang; Lu,Jian; Lan,Yu; Shu,Yuelong
A/Environment/Jiangxi/10615/2014_H10N8	EPI530418		China	10-Jan-2014	WHO Chinese National Influenza Center	WHO Chinese National Influenza Center	Wang,Dayan; Gao,Rongbao; Yang,Lei; Zhou,Shumei; Li,Xiyan; Zhou,Jianfang; Zhu,Wenfei; Guo,Junfeng; Li,Xiaodan; Dong,Jie; Huang,Weijuan; Zhang,Ye; Dong,Libo; Zhao,Xiang; Lu,Jian; Lan,Yu; Shu,Yuelong
A/Environment/Jiangxi/10721/2014_H10N8	EPI530426		China	15-Jan-2014	WHO Chinese National Influenza Center	WHO Chinese National Influenza Center	Wang,Dayan; Gao,Rongbao; Yang,Lei; Zhou,Shumei; Li,Xiyan; Zhou,Jianfang; Zhu,Wenfei; Guo,Junfeng; Li,Xiaodan; Dong,Jie; Huang,Weijuan; Zhang,Ye; Dong,Libo; Zhao,Xiang; Lu,Jian; Lan,Yu; Shu,Yuelong
A/Environment/Jiangxi/10738/2014_H10N8	EPI530434		China	14-Jan-2014	WHO Chinese National Influenza Center	WHO Chinese National Influenza Center	Wang,Dayan; Gao,Rongbao; Yang,Lei; Zhou,Shumei; Li,Xiyan; Zhou,Jianfang; Zhu,Wenfei; Guo,Junfeng; Li,Xiaodan; Dong,Jie; Huang,Weijuan; Zhang,Ye; Dong,Libo; Zhao,Xiang; Lu,Jian; Lan,Yu; Shu,Yuelong
A/Jiangxi-Donghu/346/2013_H10N8	EPI497477		China	4-Dec-2013	-	WHO Chinese National Influenza Center	Rongbao,Gao; Shumei,Zou; Xiang,Zhao; Lei,Yang; Dayan,Wang; Yuelong,Shu
A/Jiangxi/09037/2014_H10N8	EPI530450		China	8-Jan-2014	WHO Chinese National Influenza Center	WHO Chinese National Influenza Center	Wang,Dayan; Gao,Rongbao; Yang,Lei; Zhou,Shumei; Li,Xiyan; Zhou,Jianfang; Zhu,Wenfei; Guo,Junfeng; Li,Xiaodan; Dong,Jie; Huang,Weijuan; Zhang,Ye; Dong,Libo; Zhao,Xiang; Lu,Jian; Lan,Yu; Shu,Yuelong
A/Mallard/Sweden/7/2003_H10N8	EPI251793		Sweden	24-May-2003	-	Other database import	Fouchier, R.
A/Northern_shoveler/Hong_Kong/MPC657/2006_H10N9	EPI469806		Hong Kong (SAR)	2006*	-	Other database import	Lam,T.T.Y.; Wang,J.; Shen,Y.; Zhou,B.; Duan,L.; Cheung,C.L.; Ma,C.; Lycett,S.J.; Leung,C.Y.H.; Chen,X.; Li,L.; Hong,W.; Chai,Y.; Zhou,L.; Liang,H.; Ou,Z.; Liu,Y.; Farooqui,A.; Kelvin,D.J.; Poon,L.L.M.; Smith,D.K.; Pybus,O.G.; Leung,G.M.; Shu,Y.; Webster,R.G.; Webby,R.J.; Peiris,J.S.M.; Rambaut,A.; Zhu,H.; Guan,Y.
A/Northern_shoveler/Hong_Kong/MPE2531/2008_H10N9	EPI469805		Hong Kong (SAR)	2008*	-	Other database import	Lam,T.T.Y.; Wang,J.; Shen,Y.; Zhou,B.; Duan,L.; Cheung,C.L.; Ma,C.; Lycett,S.J.; Leung,C.Y.H.; Chen,X.; Li,L.; Hong,W.; Chai,Y.; Zhou,L.; Liang,H.; Ou,Z.; Liu,Y.; Farooqui,A.; Kelvin,D.J.; Poon,L.L.M.; Smith,D.K.; Pybus,O.G.; Leung,G.M.; Shu,Y.; Webster,R.G.; Webby,R.J.; Peiris,J.S.M.; Rambaut,A.; Zhu,H.; Guan,Y.
A/Northern_shoveler/Hong_Kong/MPE2984/2008_H10N9	EPI469808		Hong Kong (SAR)	2008*	-	Other database import	Lam,T.T.Y.; Wang,J.; Shen,Y.; Zhou,B.; Duan,L.; Cheung,C.L.; Ma,C.; Lycett,S.J.; Leung,C.Y.H.; Chen,X.; Li,L.; Hong,W.; Chai,Y.; Zhou,L.; Liang,H.; Ou,Z.; Liu,Y.; Farooqui,A.; Kelvin,D.J.; Poon,L.L.M.; Smith,D.K.; Pybus,O.G.; Leung,G.M.; Shu,Y.; Webster,R.G.; Webby,R.J.; Peiris,J.S.M.; Rambaut,A.; Zhu,H.; Guan,Y.
A/Shoveler/Egypt/00004-NAMRU3/2007_H10N9	EPI372434		Egypt	29-Dec-2006	U.S. Naval Medical Research Unit No.3	Centers for Disease Control and Prevention	Gerloff, Nancy; Simpson, Natosha; Jones, Joyce; Kis, Zoltan; Bahgat, Verina; Soliman,Atef; Elassal, Emad; Ahmed, Lu'ay; Gaynor, Anne; Cornelius, Claire; Davis,Todd
A/White-fronted_goose/Germany-NI/R482/09_H1N1	EPI248525	EPI248524	Germany	2009*	-	Friedrich-Loeffler-Institut	-
A/Domestic_goose/Germany-MV/R3298/2009_H6N1		EPI339177	Germany	2009*	-	Friedrich-Loeffler-Institut	-
A/Mallard/Italy/4518/2007_H10N1		EPI511812	Italy	2007*	Istituto Zooprofilattico Sperimentale Delle Venezie	Istituto Zooprofilattico Sperimentale Delle Venezie	-
A/Wild_duck/Germany/WV2555/2006_H3N2		EPI185342	Germany	2006*	-	Friedrich-Loeffler-Institut	-
A/Pheasant/Ireland/PV12-010728/12_H5N2		EPI375596	Ireland	24-Mar-2012	Central Veterinary Research Laboratory	Animal and Plant Health Agency (APHA)	Puranik, A; Thomas, S; Hanna, A; Essen, S; Focosi-Snyman, R; Manvell, R.J; Raleigh, P; Flynn, O; Reid, S

Isolate name	Accession number HA segment	Accession number NA segment	Country	Collection Date	Originating Laboratory	Submitting Laboratory	Authors
A/Chicken/Italy/11VIR-7548/2011_H5N2		EPI464929	Italy	28-Dec-2012	Istituto Zooprofilattico Sperimentale Delle Venezie	Istituto Zooprofilattico Sperimentale Delle Venezie	Monne, I.; Salviato, A.; Tassoni, L.; Cattoli, G.
A/Turkey/Italy/12VIR-6607-5/2012_H5N2		EPI464937	Italy	31-Aug-2012	Istituto Zooprofilattico Sperimentale Delle Venezie	Istituto Zooprofilattico Sperimentale Delle Venezie	Monne, I.; Salviato, A.; Tassoni, L.; Cattoli, G.
A/Chicken/Italy/12VIR-7785-67/2012_H5N2		EPI464945	Italy	28-Sep-2012	Istituto Zooprofilattico Sperimentale Delle Venezie	Istituto Zooprofilattico Sperimentale Delle Venezie	Monne, I.; Salviato, A.; Tassoni, L.; Cattoli, G.
A/Turkey/Italy/12VIR-8036-2/2012_H5N2		EPI464953	Italy	28-Jun-2012	Istituto Zooprofilattico Sperimentale Delle Venezie	Istituto Zooprofilattico Sperimentale Delle Venezie	Monne, I.; Salviato, A.; Tassoni, L.; Cattoli, G.
A/Mule_duck/Bulgaria/61/2010_mixed		EPI574173	Bulgaria	10-Jan-2010	-	Other database import	Marinove-Petkova,A.; Georgiev,G.; Petkov,T.; Darnell,D.; Franks,J.; Walker,D.; Seiler,P.; Danner,A.; Graham,A.; McKenzie,P.; Krauss,S.; Webby,R.J.; Webster,R.G.
A/Mule_duck/Bulgaria/64/2010_mixed		EPI574211	Bulgaria	9-Jan-2010	-	Other database import	Marinove-Petkova,A.; Georgiev,G.; Petkov,T.; Darnell,D.; Franks,J.; Walker,D.; Seiler,P.; Danner,A.; Graham,A.; McKenzie,P.; Krauss,S.; Webby,R.J.; Webster,R.G.
A/Mule_duck/Bulgaria/369/2009_H4N2		EPI574213	Bulgaria	16-Mar-2009	-	Other database import	Marinove-Petkova,A.; Georgiev,G.; Petkov,T.; Darnell,D.; Franks,J.; Walker,D.; Seiler,P.; Danner,A.; Graham,A.; McKenzie,P.; Krauss,S.; Webby,R.J.; Webster,R.G.
A/Mule_duck/Bulgaria/105/2008_mixed		EPI574258	Bulgaria	16-Dec-2008	-	Other database import	Marinove-Petkova,A.; Georgiev,G.; Petkov,T.; Darnell,D.; Franks,J.; Walker,D.; Seiler,P.; Danner,A.; Graham,A.; McKenzie,P.; Krauss,S.; Webby,R.J.; Webster,R.G.
A/Mule_duck/Bulgaria/174/2009_H6N2		EPI574265	Bulgaria	12-Jan-2009	-	Other database import	Marinove-Petkova,A.; Georgiev,G.; Petkov,T.; Darnell,D.; Franks,J.; Walker,D.; Seiler,P.; Danner,A.; Graham,A.; McKenzie,P.; Krauss,S.; Webby,R.J.; Webster,R.G.
A/Mule_duck/Bulgaria/596/2010_H4N2		EPI574276	Bulgaria	29-Mar-2010	-	Other database import	Marinove-Petkova,A.; Georgiev,G.; Petkov,T.; Darnell,D.; Franks,J.; Walker,D.; Seiler,P.; Danner,A.; Graham,A.; McKenzie,P.; Krauss,S.; Webby,R.J.; Webster,R.G.
A/Ruddy_Turnstone/Delaware/67/98_H12N4		EPI16616	USA	1998*	-	Other database import	-
A/Pintail/Alaska/314/2005_H12N4		EPI307548	USA	11-Aug-2005	-	Other database import	The NIAID Influenza Genome Sequencing Consortium
A/Red_knot/Delaware_Bay/227/1994_mixed		EPI345275	USA	23-May-1994	-	Other database import	The NIAID Influenza Genome Sequencing Consortium
A/Shorebird/Delaware_Bay/215/1994_mixed		EPI437174	USA	23-May-1994	-	Other database import	Wentworth,D.E.; Dugan,V.; Halpin,R.; Lin,X.; Bera,J.; Ghedin,E.; Fedorova,N.; Overton,L.; Tsitrin,T.; Stockwell,T.; Amedeo,P.; Bishop,B.; Chen,H.; Edworthy,P.; Gupta,N.; Katzel,D.; Li,K.; Schobel,S.; Shrivastava,S.; Thovarai,V.; Wang,S.; Runstadler,J.; Lindberg,M.; Huettmann,F.; Petrula,M.; Meixell,B.; Gingrich,J.P.; Gildehaus,L.A.; Vick,L.; Kokx,K.; Dillon,D.; Aldehoff,F.; Felker,E.P.; Marcotte,R.W.; Schmidt,J.C.; Moore,J.R.; Gerdes,K.E.; Bao,Y.; Sanders,R.; Dernovoy,D.; Kiryutin,B.; Lipman,D.J.; Tatusova,T.
A/Ruddy_turnstone/Delaware_Bay/124/1994_mixed		EPI437442	USA	22-May-1994	-	Other database import	Wentworth,D.E.; Dugan,V.; Halpin,R.; Lin,X.; Bera,J.; Ghedin,E.; Fedorova,N.; Overton,L.; Tsitrin,T.; Stockwell,T.; Amedeo,P.; Bishop,B.; Chen,H.; Edworthy,P.; Gupta,N.; Katzel,D.; Li,K.; Schobel,S.; Shrivastava,S.; Thovarai,V.; Wang,S.; Runstadler,J.; Lindberg,M.; Huettmann,F.; Petrula,M.; Meixell,B.; Gingrich,J.P.; Gildehaus,L.A.; Vick,L.; Kokx,K.; Dillon,D.; Aldehoff,F.; Felker,E.P.; Marcotte,R.W.; Schmidt,J.C.; Moore,J.R.; Gerdes,K.E.; Bao,Y.; Sanders,R.; Dernovoy,D.; Kiryutin,B.; Lipman,D.J.; Tatusova,T.
A/Ruddy_turnstone/Delaware_Bay/150/1994_H1N4		EPI437470	USA	22-May-1994	-	Other database import	Wentworth,D.E.; Dugan,V.; Halpin,R.; Lin,X.; Bera,J.; Ghedin,E.; Fedorova,N.; Overton,L.; Tsitrin,T.; Stockwell,T.; Amedeo,P.; Bishop,B.; Chen,H.; Edworthy,P.; Gupta,N.; Katzel,D.; Li,K.; Schobel,S.; Shrivastava,S.; Thovarai,V.; Wang,S.; Runstadler,J.; Lindberg,M.; Huettmann,F.; Petrula,M.; Meixell,B.; Gingrich,J.P.; Gildehaus,L.A.; Vick,L.; Kokx,K.; Dillon,D.; Aldehoff,F.; Felker,E.P.; Marcotte,R.W.; Schmidt,J.C.; Moore,J.R.; Gerdes,K.E.; Bao,Y.; Sanders,R.; Dernovoy,D.; Kiryutin,B.; Lipman,D.J.; Tatusova,T.
A/Blue-winged_teal/ALB/685/1982_H6N4		EPI85929	Canada	20-Aug-1982	-	Other database import	-
A/Mallard_duck/Alberta/299/1977_H4N4		EPI87231	Canada	10-Aug-1977	-	Other database import	-
A/Mallard/Alberta/194/1992_H8N4		EPI87925	Canada	1-Dec-1992	-	Other database import	-
A/Duck/Hokkaido/24/04_H10N5		EPI160652	Japan	2004*	-	Other database import	-
A/Mallard/Denmark/77-64590-5/2005_H7N5		EPI174859	Denmark	19-Sep-2005	Technical University of Denmark	National Veterinary Institute	-
A/Mallard/California/6524/2008_H12N5		EPI328292	USA	5-Nov-2008	-	Other database import	The NIAID Influenza Genome Sequencing Consortium
A/Mallard/Alberta/220/2006_		EPI343416	Canada	9-Aug-2006	-	Other database import	The NIAID Influenza Genome Sequencing Consortium
A/Mallard/Alberta/12/1993_		EPI344668	Canada	20-Aug-1993	-	Other database import	The NIAID Influenza Genome Sequencing Consortium
A/Arenaria_interpres/Belgium/02936pcs1/2010_H12N5		EPI345387	Belgium	27-Feb-2010	Veterinary and Agrochemical Research Institute	Veterinary and Agrochemical Research Institute	Van Borm,S.; Rosseel,T.;Lambrecht,B.; Vangeluwe,D.; Vandenbussche,F.; van den Berg,T.
A/Mallard/Ohio/170/1999_H6N5		EPI44086	USA	23-Oct-1999	-	Other database import	-
A/Emperor_goose/Alaska/44064-075/2006_H2N5		EPI442443	USA	25-May-2006	-	Other database import	Reeves,A.B.; Pearce,J.M.; Ramey,A.M.; Ely,C.R.; Schmutz,J.A.; Flint,P.L.; Derksen,D.V.; Ip,H.S.; Trust,K.A.
A/Mallard/Minnesota/182729/1998_H6N5		EPI448392	USA	1-Sep-1998	-	Other database import	Wentworth,D.E.; Dugan,V.; Halpin,R.; Lin,X.; Bera,J.; Wester,E.; Ghedin,E.; Fedorova,N.; Tsitrin,T.; Stockwell,T.; Amedeo,P.; Bishop,B.; Edworthy,P.; Gupta,N.; Katzel,D.; Li,K.; Schobel,S.; Shrivastava,S.; Thovarai,V.; Wang,S.; Lebarbenchon,C.; Sreevatsan,S.; Poulson,B.; Yang,M.; Stallknecht,D.; Bao,Y.; Sanders,R.; Dernovoy,D.; Kiryutin,B.; Lipman,D.J.; Tatusova,T.
A/Green-winged_teal/Minnesota/Sg-00820/2008_H4N5		EPI449492	USA	3-Sep-2008	-	Other database import	Wentworth,D.E.; Dugan,V.; Halpin,R.; Lin,X.; Bera,J.; Wester,E.; Ghedin,E.; Fedorova,N.; Tsitrin,T.; Stockwell,T.; Amedeo,P.; Bishop,B.; Edworthy,P.; Gupta,N.; Katzel,D.; Li,K.; Schobel,S.; Shrivastava,S.; Thovarai,V.; Wang,S.; Lebarbenchon,C.; Sreevatsan,S.; Poulson,B.; Yang,M.; Stallknecht,D.; Bao,Y.; Sanders,R.; Dernovoy,D.; Kiryutin,B.; Lipman,D.J.; Tatusova,T.
A/Ruddy_turnstone/New_Jersey/AI07-697/2007_H12N5		EPI454987	USA	10-Aug-2007	-	Other database import	Wentworth,D.E.; Dugan,V.; Halpin,R.; Lin,X.; Bera,J.; Wester,E.; Ghedin,E.; Fedorova,N.; Tsitrin,T.; Stockwell,T.; Amedeo,P.; Bishop,B.; Edworthy,P.; Gupta,N.; Katzel,D.; Li,K.; Schobel,S.; Shrivastava,S.; Thovarai,V.; Wang,S.; Lebarbenchon,C.; Sreevatsan,S.; Poulson,B.; Yang,M.; Stallknecht,D.; Bao,Y.; Sanders,R.; Dernovoy,D.; Kiryutin,B.; Lipman,D.J.; Tatusova,T.

Isolate name	Accession number HA segment	Accession number NA segment	Country	Collection Date	Originating Laboratory	Submitting Laboratory	Authors
A/Ruddy_turnstone/New_Jersey/AI07-803/2007_H12N5		EPI455001	USA	16-Aug-2007	-	Other database import	Wentworth,D.E.; Dugan,V.; Halpin,R.; Lin,X.; Bera,J.; Wester,E.; Ghedin,E.; Fedorova,N.; Tsitrin,T.; Stockwell,T.; Amedeo,P.; Bishop,B.; Edworthy,P.; Gupta,N.; Katzel,D.; Li,K.; Schobel,S.; Shrivastava,S.; Thovarai,V.; Wang,S.; Lebarbenchon,C.; Sreevatsan,S.; Poulson,B.; Yang,M.; Stallknecht,D.; Bao,Y.; Sanders,R.; Dernovoy,D.; Kiryutin,B.; Lipman,D.J.; Tatusova,T.
A/Mule_duck/Bulgaria/674/2010_H6N5		EPI574266	Bulgaria	12-Apr-2010	-	Other database import	Marinove-Petkova,A.; Georgiev,G.; Petkov,T.; Darnell,D.; Franks,J.; Walker,D.; Seiler,P.; Danner,A.; Graham,A.; McKenzie,P.; Krauss,S.; Webby,R.J.; Webster,R.G.
A/Mallard/Alberta/202/1996_H2N5		EPI85385	Canada	1996*	-	Other database import	-
A/Pintail/Alberta/49/2003_H9N5		EPI86317	Canada	2003*	-	Other database import	-
A/Mallard/Alberta/52/1997_H12N5		EPI86417	Canada	31-Oct-1997	-	Other database import	-
A/Green-winged_teal/ALB/199/1991_H12N5		EPI88775	Canada	26-Aug-1991	-	Other database import	-
A/Mallard/Poland/16/09_H7N7		EPI254380	Poland	12-Jan-2009	National Veterinary Research Institute	National Veterinary Research Institute	-
A/Turkey/Netherlands/03003568/03_H7N7		EPI290239	Netherlands	9-Mar-2003	-	Central Veterinary Institute	-
A/Chicken/Germany/R1801/2011_H7N7		EPI356304	Germany	2011*	-	Friedrich-Loeffler-Institut	-
A/Turkey/Germany/R1775/2011_H7N7		EPI356305	Germany	2011*	-	Friedrich-Loeffler-Institut	-
A/Turkey/Germany-NI/R534/2013_H7N7		EPI470367	Germany	11-Apr-2013	-	Friedrich-Loeffler-Institut	-
