# Supplementary material for: Discordant detection of avian influenza virus subtypes in time and space between poultry and wild birds; Towards improvement of surveillance programs
Source: PLoS One. 2017 Mar 9;12(3):e0173470. doi: 10.1371/journal.pone.0173470 (PMC5344487; doi:10.1371/journal.pone.0173470)
Supplement: S3 Table — Number and percentage (between brackets) of hemagglutinin (A) and neuraminidase (B) subtypes are shown for poultry and wild bird species. Poultry subtypes are shown for primary and secondary cases (i.e. all combined) and separate for primary cases only (i.e. primary cases). Subtypes indicated with an asterisk were significantly more or less frequently detected in the corresponding group than in all wild birds combined, with * = P <0.05 and ** = P <0.01 (Fisher’s exact test). (PDF) [file pone.0173470.s005.pdf]

Supporting Information

S3 Table. Avian influenza virus hemagglutinin and neuraminidase subtype distribution among poultry and wild bird species, the Netherlands, 2006 - 2011. Number and percentage (between brackets) of hemagglutinin (A) and neuraminidase (B) subtypes are shown for poultry and wild bird species. Poultry subtypes are shown for primary and secondary cases (i.e. all combined) and separate for primary cases only (i.e. primary cases). Subtypes indicated with an asterisk were significantly more or less frequently detected in the corresponding group than in all wild birds combined, with * = P <0.05 and ** = P <0.01 (Fisher's exact test).

A

Subtype	Poultry		Wild birds						
	All combined	Primary cases	All combined	Anseriformes				Charadriiformes	
				Mallard	Other duck species	Goose species	Swan species	Gull species	Wader species
	70	20	542	250 (46)	20 (4)	40 (7)	16 (3)	201 (37)	15 (3)
H1	11 (16)**	2 (10)	30 (6)	18 (7)	2 (10)	5 (13)	4 (25)	1 (<1)	-
H2	2 (3)	1 (5)	11 (2)	10 (4)	1 (5)	-	-	-	-
H3	-	-	89 (16)	75 (30)**	3 (15)	-	1 (6)	-	10 (67)**
H4	-	-	58 (11)	49 (20)**	1 (5)	-	5 (31)*	3 (1)	-
H5	9 (13)**	1 (5)	25 (5)	15 (6)	2 (10)	7 (18)**	1 (6)	-	-
H6	10 (14)	3 (15)	53 (10)	23 (9)	4 (20)	25 (63)**	1 (6)	-	-
H7	15 (21)**	6 (30)	26 (5)	22 (9)*	1 (5)	2 (5)	1 (6)	-	-
H8	15 (21)**	1 (5)	5 (1)	4 (2)	1 (5)	-	-	-	-
H9	5 (7)**	4 (20)	5 (1)	-	2 (10)*	1 (3)	2 (13)*	-	-
H10	3 (4)	2 (10)	34 (6)	25 (10)	2 (10)	-	1 (6)	1 (<1)	5 (33)**
H11	-	-	9 (2)	7 (3)	-	-	-	2 (1)	-
H12	-	-	3 (1)	2 (1)	1 (5)	-	-	-	-
H13	-	-	111 (20)	-	-	-	-	111 (55)**	-
H16	-	-	83 (15)	-	-	-	-	83 (41)**	-

B

Subtype	Poultry		Wild birds						
	All combined	Primary cases	All combined	Anseriformes				Charadriiformes	
				Mallard	Other duck species	Goose species	Swan species	Gull species	Wader species
	32	11	542	250 (46)	20 (4)	40 (7)	16 (3)	201 (37%)	15 (3)
N1	5 (16)	2 (18)	57 (11)	35 (14)	8 (40)**	9 (23)*	4 (25)	1 (<1)**	-
N2	2 (6)	2 (18)	79 (15)	37 (15)	5 (25)	13 (33)**	3 (19)	21 (10)	-
N3	2 (6)	2 (18)	105 (19)	19 (8)**	1 (5)	1 (3)**	-	84 (42)**	-
N4	6 (19)**	1 (9)	12 (2)	5 (2)	1 (5)	-	-	1 (<1)	5 (33)**
N5	8 (25)**	1 (9)	14 (3)	10 (4)	1 (5)	1 (3)	1 (6)	1 (<1)	-
N6	-	-	62 (11)	49 (20)**	-	-	6 (38)**	7 (3)**	-
N7	8 (25)**	3 (27)	27 (5)	26 (10)**	-	-	1 (6)	-	-
N8	1 (3)**	-	179 (33)	63 (25)*	4 (20)	16 (40)	1 (6)*	85 (42)*	10 (67)*
N9	-	-	7 (1)	6 (2)	-	-	-	1 (<1)	-
